# The Activation of ARF1 Is Dynamically Regulated by its Palmitoylation

**DOI:** 10.1016/j.mcpro.2026.101586

**Published:** 2026-05-14

**Authors:** Jiangli Zhu, Wenchao Li, Mingyang Ji, Xue Chang, Yang Cao, Jun Gao, Shiqian Qi, Eryan Kong

**Affiliations:** 1 Department of Ophthalmology, The First Affiliated Hospital of Henan Medical University, Xinxiang, China; 2 Department of Urology, Institute of Urology, State Key Laboratory of Biotherapy, West China Hospital, Sichuan University, and National Collaborative Innovation Center, Chengdu, China; 3 Xinxiang Key Laboratory of Protein Palmitoylation and Major Human Diseases, Institute of Psychiatry and Neuroscience, Henan Medical University, Xinxiang, China; 4 School of Stomatology, The Third Affiliated Hospital of Henan Medical University, Xinxiang, China; 5 Center of Growth, Metabolism, and Aging, Key Laboratory of Bio-Resource and Eco-Environment of Ministry of Education, College of Life Sciences, Sichuan University, Chengdu, China

**Keywords:** ARF1, ARF1 activation, dynamic palmitoylation, PPT2, ZDHHC8

## Abstract

ARF1 plays a crucial role in maintaining the structure and function of the Golgi apparatus, yet the precise on-site regulatory mechanism governing ARF1 activation remains incompletely understood. Here, we have uncovered a novel regulatory mechanism involving the palmitoylation of ARF1 at Cys159. Our findings in HEK293T cells demonstrate that ZDHHC8 is responsible for promoting ARF1 palmitoylation, while PPT2 is involved in the depalmitoylation of ARF1. Furthermore, our results indicate that mutation of Cys159 to Alanine abolishes ARF1 palmitoylation, leading to increased localization of ARF1 at the Golgi apparatus. Interestingly, we observed that upregulation of ARF1 palmitoylation by ZDHHC8 inhibits ARF1 activation, while downregulation of ARF1 palmitoylation through the expression of PPT2 or Cys159 to Alanine enhances ARF1 activation. Mechanistically, we propose that ARF1 palmitoylation plays a crucial role in regulating retrograde transport by interacting with key proteins such as β-COP and β-actin. Our study presents a model in which the activation of ARF1 is dynamically regulated by its palmitoylation status, shedding light on a previously unrecognized mechanism for controlling ARF1 function at the Golgi apparatus. These findings provide valuable insights into the regulatory pathways governing intracellular protein transport and cellular function.

## Highlights

• ARF1 is identified as a novel palmitoylated protein, with the modification occurring at a single conserved cysteine residue (Cys159).
• The reversible palmitoylation of ARF1 is dynamically regulated by the palmitoyltransferase ZDHHC8 and the thioesterase PPT2.
• Palmitoylation negatively regulates ARF1 activation; blocking this modification increases the GTP-bound, active form of ARF1.
• The C159A mutation (blocking palmitoylation) enhances ARF1 localization at the Golgi apparatus and strengthens its interaction with retrograde transport machinery components β-COP and β-actin.
• Molecular dynamics simulations suggest a structural mechanism wherein palmitoylation at Cys159 sterically hinders the guanine nucleotide-binding pocket, potentially impeding GDP-to-GTP exchange.

## Graphical Abstract

**Figure:**
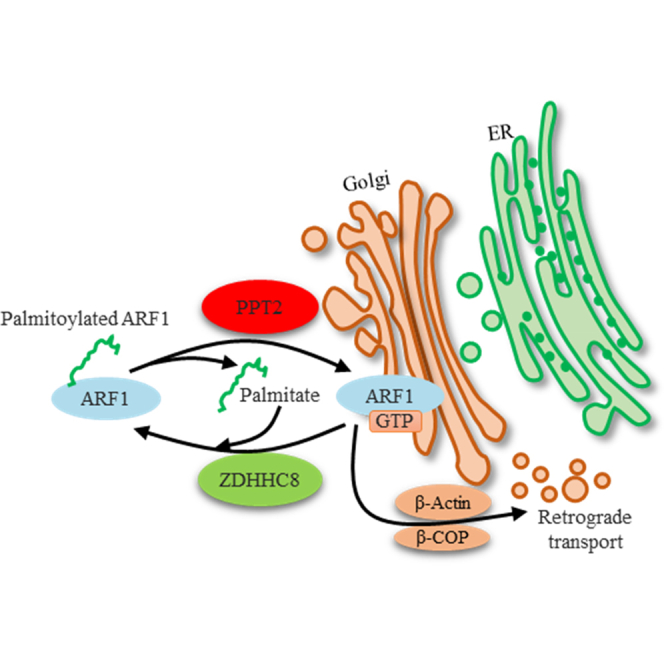

## In Brief

This study identifies reversible S-palmitoylation of ARF1 at Cys159 by ZDHHC8 and PPT2. Contrary to typical roles of palmitoylation in promoting membrane association, ARF1 palmitoylation functions as a negative regulator of its activation. Inhibition of palmitoylation increases GTP-bound ARF1, enhances its Golgi localization, and promotes its effector interaction of retrograde transport. Structural modeling suggests palmitoylation may sterically interfere with the nucleotide-binding pocket. These findings establish dynamic palmitoylation as a critical switch controlling ARF1 activity and its role in Golgi function.

Protein S-acylation, hereafter named palmitoylation, is one of the most common and reversible lipid modifications in proteins, which specifically occurs on cysteine residue through thioester linkage (1, 2). The dynamics of this modification is accomplished by palmitoylation, catalyzed by ZDHHC1-25 (ZDHHCs 1–9 and 11–25, there is no known isoform 10), and depalmitoylation, catalyzed by protein thioesterases (APT1/2, PPT1/2, and ABHD17a/b/c) (3, 4, 5, 6, 7). Due to the hydrophobic nature of palmitic acid (a 16-carbon saturated fatty acid), palmitoylation is involved in modulating candidate proteins in various ways e.g., membrane localization, protein-protein interaction, and signaling transduction (8, 9, 10).

The ADP-ribosylation factor (ARF) proteins are small, ubiquitously expressed guanosine triphosphate (GTP)-binding proteins best known for their role in membrane trafficking by recruiting coat proteins (11, 12, 13). ARFs are also involved in lipid metabolism by recruiting and activating critical enzymes, such as the phosphatidyl-inositol (PtdIns) kinases (14, 15), and modulating the structure of organelles by regulating cytoskeletal proteins (16, 17, 18). Moreover, ARF1 is one of the necessary host factors during HIV-1 infection (19, 20, 21). Typically, ARFs localize to the plasma membrane (PM) and the membranes of the ER, Golgi, and lysosome, which relies on the myristoylation at the N-terminus and the N-terminal amphipathic helix (2–13 peptide) (22, 23).

In mammals, six ARFs can be divided into three classes based on sequence similarities, with class I containing ARF1-3, class II containing ARF4-5, and class III containing only ARF6 (17). Class I and II mainly localize at the Golgi apparatus, although they also function at endosomes. By contrast, ARF6 is localized primarily at the PM and a subset of endosomes (24, 25). In addition, there are over 20 ARF-like proteins, which seem to have broader roles than ARFs (26, 27, 28, 29). Studies of ARF1 at the Golgi and ARF6 at the PM provide substantial insight into their function and regulatory mechanism (11, 14, 16, 18, 30, 31, 32). The Golgi plays important role in the post-translation modification of proteins and intracellular cargo transportation (33, 34). ARF1 localizes to the early cis-Golgi, where it activates PtdIns4K to generate PtdIns4P for maintenance of Golgi integrity/function, and engages in the recruitment of COPs and APs to facilitate the transportation of cargos, *e.g.*, lipids, TMP21, and BIP, from the trans-Golgi to the cis-Golgi and also at the TGN (12, 14, 23, 35, 36, 37). Under such a scenario, the activation of ARF1 is strictly regulated, where a coincidence of PtdInsP_2_ and/or PtdInsP_3_, acidic phospholipids, and an active ARF1 (an exchange of GDP-bound to GTP-bound mediated by guanine nucleotide exchange factor (GEF)) must occur simultaneously (15, 38). The dysregulation of ARF1 activation might cause a devastating outcome: mutations in ARF1 GEF BIG2 are linked to autosomal recessive periventricular heterotopia, a disease in which the cerebral cortex is severely underdeveloped owing to the failure of neuronal migration (39).

Besides myristoylation (22), whether another form of post-translation modification e.g., palmitoylation, also occurs on ARFs has not been explored. Interestingly, carrying out a pilot study to identify potential palmitoylated proteins by palm-proteomics, ARF1 was spotted, indicating that ARF1 is possibly modified with palmitoylation (Supplemental data S1). Since palmitoylation regulates protein subcellular localization, protein-protein interaction, and activity/signaling (4, 10, 40), we wondered whether ARF1 palmitoylation is also involved in these cellular processes.

## Experimental Procedures

### Mice

Fertilized B6 eggs were harvested and injected with a microinjection system. In brief, Cas9 mRNA and sgRNA (AGGGTACTGCTCCCCGCACGGGG, GGCCCCTAATAGGCCAGGTTTGG) were generated by using *in vitro* transcript (IVT) kits, all components were mixed well and injected into the cytoplasm of fertilized eggs. Handled eggs were cultured to a two-cell stage and transferred into ICR foster mice. Mice were identified by genotyping with the following primer pairs: *PPT2*-F-AGCCTTCCCTCATACGGACCAG and *PPT2*-R-CAAGTGCTAGGATTAACTATTGCTGGC or *PPT2*-He/Wt-R-GCCTTAGATAAGGGTCAAGCTCAGAC. Sequences for the primer pairs were derived from GenBank (NM_001302393.1). Primer *PPT2*-F and primer *PPT2*-He/Wt-R amplify the endogenous mouse sequence and give a PCR product of 709 bp. Primer *PPT2*-F and primer *PPT2*-R amplify the KO cassette insertion site and give a PCR product of 840 bp. Mice were housed in a temperature-controlled facility under pathogen-free conditions and a 12-h light/dark cycle at 23 to 25 °C with free access to food and water. All animal procedures were performed according to guidelines approved by the ethics committee on animal care at Xinxiang Medical University (XYLL-20220128).

### Cell Culture and Transfection

The HEK-293T (CRL-11268), A-549 lung cancer cell line (CCL-185) and HCT-116 colorectal CCL-247 were purchased from ATCC. The HeLa cervical adenocarcinoma was purchased from Cell Bank/Stem Cell Bank, Chinese Academy of Sciences. HEK-293T and HCT-116 were cultured in Dulbecco’s MEM (DMEM) high glucose medium (SH30022.01, HyClone) supplemented with 10% fetal bovine serum (10099141, Gibco) and 1% penicillin-streptomycin (15070063, Gibco). HeLa was cultured in Minimum Essential Medium (MEM) (SH30024.01B, HyClone) supplemented with 10% fetal bovine serum and 1% penicillin-streptomycin. A-549 was cultured in basic RPMI 1640 Medium 1640 (C11875500BT, Gibco) supplemented with 10% fetal bovine serum and 1% penicillin-streptomycin. Transient transfection was performed using Lipofectamine 3000 (L3000-015, Invitrogen) with the reduced serum medium Opti-MEM (51985091, Gibco) according to the manufacturer’s instructions. The following reagents were used for cell treatments: 2-bromopalmitate (2-BP) (238422, Sigma-Aldrich), PalmB (178501, Merck Millipore), and DMSO (D8418, Sigma Aldrich). All drug-treatment experiments were started at a cell confluence of 70 to 80% in 10-cm dishes.

### General Reagents and Antibodies

Primary antibodies used in this study: Flag (AE024, ABclonal), ARF1 (sc-53168, Santa Cruz), β-actin (AC026, ABclonal), β-COP (sc-393615, Santa Cruz), Calnexin (sc-46669, Santa Cruz), β-adaptin (sc-74423, Santa Cruz), γ-adaptin (sc-398867, Santa Cruz), TMP21 (sc-137003, Santa Cruz), Bip (BF8024, Affinity BioReagents), Na/K ATPase (A11683, Abclonal). Secondary antibodies used in this study: Goat Anti-Mouse IgG (H+L), HRP Conjugate (PMS301, Protein Biotechnologies), Goat Anti-Rabbit IgG (H+L), HRP Conjugate (PMS302, Protein Biotechnologies) and Goat-anti-Mouse IgG (H+L), Fluor 594 (A-21235, Invitrogen). LysoView633 (#70058, Biotium) was used for live cell imaging. See details of reagents in Supplemental Table S1.

### Plasmids

The plasmids expressing mouse-origin pCMV3-ARF1-Flag were constructed, as well as pCMV3-APT1-Flag, pCMV3-APT2-Flag, pCMV3-PPT1-Flag, pCMV3-PPT1- Flag and pCMV3-ABHD17a-Flag. HA-tagged ZDHHC-PATs were gifts from Dr Fukata’s lab. ARF1^C159A^-Flag point-mutation construct was generated from murine ARF1-Flag in pCMV3-ARF1-Flag vector by PCR-based site-directed mutagenesis. ARF1-GFP-Flag and ARF1-C159A-GFP-Flag were generated from murine ARF1-Flag in pLV-EF1α-MCS-IRES-GFP. PPT2-RFP-Flag and RFP-PPT2-Flag were generated from murine PPT2-Flag in pLV-EF1α-MCS-IRES-RFP vector. All constructs were verified by sequence analysis.

### Generation of ARF1-KO, PPT2-KO or ZDHHC8-KO HEK-293T cell

For deleting ARF1 in HEK-293T cells, two guide RNA (gRNA) against the second exon in the human *ARF1* genome were designed, using an online tool (http://crispor.tefor.net/) (41). The forward gRNA sequence (CCCGCACCCAGGCTTCAACG) and the reverse gRNA sequence (ATAGATGGGGCATCGATGCC) are against the upstream and downstream of exon 2 respectively. The double-stranded oligos for each gRNA were synthesized by Shanghai Bioligo Biotechnology and cloned into the pX458 vector (48138, Addgene). The plasmids were co-transfected into HEK-293T cells by Lipofectamine 3000 transfection kit (L3000-015, Invitrogen). Single fluorescence cell was sorted by fluorescence-activated cell sorting (BD Biosciences) to pick the single cell clone. KO cells were confirmed by genotyping, sequencing of PCR-amplified genomic DNA, and immunoblot. The pair of primers: Forward-5′-GGGAACATCTTCGCCAACCTCT-3′ and Reverse-5′-AGCCTCCAGTAACCCCTACCAC-3′ were used for genotyping. Genomic DNA was extracted using a universal DNA purification kit (TIANGEN, 4992197).

For deleting PPT2 in HEK-293T cells, two gRNA against the second exon in the human *PPT2* genome were designed. The forward gRNA sequence (GAAGCAGGACCCACGCCGCGGGG) and the reverse gRNA sequence (GTCCTACAAGCCGGTCATCGTGG) are against the upstream and downstream of exon 2 respectively. KO cells were confirmed by genotyping, sequencing of PCR-amplified genomic DNA. The pair of primers (Forword-5′- TGTTGCCATCTCCCTCACAT -3′ and Reverse-5′- CCCTGCCAGACCTCATTGAT -3′) were used for genotyping.

For deleting ZDHHC8 in HEK-293T cells, two gRNA against the second exon in the human *ZDHHC8* genome were designed. The forward gRNA sequence (TGGTGTTTTCCCCCGAGGTAGGG) and the reverse gRNA sequence (GACACAGCTCGTGTCAACCACGG) are against the upstream and downstream of exon 2 respectively. KO cells were confirmed by genotyping, sequencing of PCR-amplified genomic DNA. The pair of primers (Forword-5′- GCACCACATAGCTCTGCG-3′ and Reverse-5′- GGAATCCCTAAGGCTGTCTCA -3′) were used for genotyping.

### Western Blotting and Antibodies

Lysates of cells or mouse tissues were prepared by a standard procedure using 1% Triton X-100 supplemented with protease inhibitors. All samples were adjusted to 1.0 μg/μl protein with PBS, added with 5× SDS loading buffer, and boiled at 100 °C for 5 min. An equal volume of protein samples was separated in 12% SDS-PAGE gels and electro-transferred in PVDF membrane (ISEQ00010, Merck Millipore,). The membrane was then blocked in 5% (w/v) skimmed milk in TBS containing 0.1% (v/v) Tween-20 for 90 min. The membranes were then incubated with appropriate primary antibodies at 4 °C overnight with shaking. After washing three times with Tween-20, the membrane was incubated with a suitable HRP labeled secondary antibody in 5% bovine serum albumin for 2 h at room temperature. The signal of the blots was detected with ECL substrates (180-501, Tanon) and the Tanon image system (Tanon 520, Tanon).

### Immunofluorescence Staining and Live Cell Imaging

Cells were plated onto poly-D-lysine-coated coverslips and transfected with the indicated plasmid, the cells were fixed with 4% (W/V) paraformaldehyde (1510, Electron Microscopy Sciences) at 24 h post-transfection, perforated with 0.1% (V/V) Triton X-100 in PBS, and blocked with 3% bovine serum albumin in PBS. Cells were incubated with primary antibodies against GM130 (1:1000) overnight at 4 °C. After washing three times with PBS, the cells were incubated with Alexa Fluor conjugated secondary antibodies. After washing and mounting onto slides with Dapi-Fluoromount-G (17984-24, Electron Microscopy Sciences), fluorescence images were acquired with a Stimulated Emission Depletion microscopy (Leica TCS SP8 STED). For living cell images, HEK-293T cells expressing ARF1-GFP and PPT2-RFP or RFP-PPT2 were seeded into glass bottom cell culture dishes (#801001, Nest), LysoView633 with a dilution of 1:1000 was added into medium and incubated for 30 min for viewing lysosome. Targeted cell was imaged per minute for 1 h at 37 °C, 5% CO2, and humidity.

### Acyl Resin-Assisted Capture (Acyl-Rac) Assay

Mouse tissues after homogenization and cells were sonicated in RIPA Lysis Buffer (P0013K, Beyotime) containing Protease inhibitor cocktail (P1006, Beyotime) and then lysed for 30 min on ice. After centrifugation at 12,000 *g* for 10 min at 4 °C, supernatants were collected for Acyl Resin-assisted Capture (Acyl-Rac). Total protein was quantified with BCA Protein Assay Kit (P0009, Beyotime). Acyl-Rac assay was performed essentially as described previously (42). 1 mg protein lysate was diluted to a concentration of 1 mg/ml in blocking buffer (100 mM Hepes pH 7.5, 1 mM EDTA, 2.5% SDS, 50 mM NEM at 50 °C for 60 min with shaking. NEM was then removed by adding three volumes of cold acetone and four sequential 70% acetone precipitations. Pellets were resuspended in 650 μl binding buffer (100 mM Hepes pH 7.5, 1 mM EDTA, 1% SDS). After taking 50 μl as the ‘input’, the binding buffer was equally divided into two parts, and added 50 μl prewashed Thiopropyl-Sepharose 6B (17-0420-01, GE Healthcare), one part was added to 40 μl 2 M Hydroxylamine pH 7.0 (431362, Sigma Aldrich) to cleave the Cys-palmitoyl thioester linkages and the other was added 40 μl 2 M NaCl as the negative control. Cleavage and capture were carried out on a rotator at room temperature for 4 h. Resins were washed five times with binding buffer. 50 μl Leammli loading buffer (2.1% SDS, 66 mM Tris-HCl pH7.5, 26% (W/V) glycerol, 50 mM DTT) was used for protein elute.

### Acyl-Biotin Exchange (ABE) Assay

Acyl-Biotin Exchange (ABE) assay was performed essentially as previously described (43). After protein concentration measurement, 1 mg of protein, diluted with LB buffer (150 mM NaCl, 50 mM Tris-HCl pH 7.4, 5 mM EDTA) to a final volume of 1 ml, were alkylated with 50 mM NEM for 90 min at room temperature to blockade of free thiols. The excess NEM was removed by chloroform-methanol precipitation and methanol wash. The samples were incubated in the presence or absence of 2 M HA and 4 mM biotin-HPDP to biotinylate the proteins at room temperature for 120 min. After chloroform-methanol precipitation and methanol wash, biotinylated proteins can be purified with Streptavidin Agarose (Merck Millipore, 69203) overnight at 4 °C. 50 μl Elute buffer (LB buffer supplied with 1% β-ME and 1×SDS-PAGE loading buffer) was used for protein elute. Each protein aliquot of ABE supernatants was analyzed by Western blotting.

### Mass Spectrometric Analysis of Palmitoylation in ARF1

30 μg purified ARF1-Flag were digested using FASP (44). Briefly, disulfide bonds were broken and blocked using 2 mM Tris (2-carboxyethyl) phosphine and 10 mM iodoacetamide, then proteins were transferred to 10 K filter, and cleaned sequentially using 8 M urea and 50 mM Tris-Hcl pH 6.8 at 13,000 *g*, 20 °C. Proteins were firstly digested with trypsin (V5113, Promega) at 1:50 (mass/mass) in 1x GluC reaction buffer at 37 °C for 4 h and then same amount of GluC was added and incubated at 37 °C for 12 h. Peptides were collected at 13000 *g*, 20 °C, lyophilized and stored at −80 °C until use. Raw files were acquired with data dependent acquisition mode using Q Exactive (Bremen, Thermo Fisher Scientific). Peptide mixture were separated on Dionex U3000 system (San Jose, Thermo Fisher Scientific) using C18 (3 μm, 75 μm × 15 cm, homemade) at a flowrate of 600 nl/min. For peptide separation, a 60-min linear gradient was set as follows: 5% B (0.1% FA in ACN)/95% A (0.1% FA in H_2_O) to 8% B in 8 min, 8% B to 13% B in 8 min, 13% B to 28% B in 23 min, 28% B to 40% B in 11 min, 40% B to 95% B in 1 min and stayed 4 min for 95% B, and column was re-equilibrated at 6% B for 4 min. For the data acquisition a top 20 scan mode with MS1 scan range m/z 300 to 1400 was used and other parameters were set as below: MS1 and MS2 resolution was set to 70K and 17.5K; AGC for MS1 and MS2 was 3e6 and 5e4; isolation window was 3.0 Th, dynamic exclusion time was 15 s. Each precursor ion was fragmented with HCD (https://pubmed.ncbi.nlm.nih.gov/32938753/) using normalized collision energy of 27. Raw file was searched against target protein sequence (https://www.uniprot.org/uniprotkb/P84078/entry) using Byonic v2.16.11 (Protein Metrics). Searching parameter was set as follows: cleavage sites of D, E, K, R with maximum number of 3 missed cleavages; precursor and fragment ion mass tolerance was set to 20 ppm and 50 ppm; variable modification was set to oxidation of M, deamidation of N, Q, carbamidomethylation of C, acetylation of protein N-term, palmitoylation of C, Y, S, T, W. For better identification of MS2 spectrums, a wildcard search function with ± 500 Da mass range was applied to all identified peptides. A target-decoy searching algorithm was applied and an automatic score cut was used to remove low score peptides. A manual check was applied to further filter high confident palmitoylated cysteine sites, palmitoylated peptides identifications with consecutive fragment ions were kept.

### LC-MS Analysis for ARF1 and ARF1-C159A Interactomes

ARF1-Flag or ARF1-C159A-Flag were expressed in HEK-293T ARF1-KO cells and purified using the Anti-DYKDDDDK Affinity resin (Sino Biological, Cat. No. 101274-MM13-RN). Next, disulfide bonds were broken and blocked using 2 mM Tris (2-carboxyethyl) phosphine and 10 mM iodoacetamide. Then proteins were digested with 10 ng/μl trypsin at 37 °C for 16 h. Post-digestion, peptides were desalted by C18 Tips, dried, redissolved in 0.1% formic acid (FA) solution, and subjected to LC-MS analysis using data-independent acquisition (DIA) acquisition method (Supplemental Table S2 and Supplemental Data S2). Equal amount of iRT calibration peptides were spiked into each sample. The peptides from each sample were analyzed by timsTOF Pro2 mass spectrometer (Bruker) connected to an Evosep One system liquid chromatography (Denmark) in the DIA mode. The mass spectrometer collected ion mobility MS spectra over a mass range of m/z 100 to 1700. Up to 4 windows were defined across the m/z-ion mobility plane for individual TIMS scans with a 100 ms accumulation time. During PASEF MSMS scanning, the mass range of m/z 400 to 1200 was divided into 32 acquisition windows with an isolation width of 26 Da and 1 Da overlap between adjacent windows. Cycle time was set to 1.8s. The collision energy was ramped linearly as a function of the mobility from 20 eV at 1/K0 = 0.85 Vs/cm2 to 59 eV at 1/K0 = 1.30 Vs/cm2. DIA data was analyzed with DIA-NN 1.9.1 searching UniProt human proteome database (downloaded on Nov, 2025, protein entries 205345) added with mouse ARF1 protein sequence (https://www.uniprot.org/uniprotkb/P84078/entry). Main software parameters were set as follows: mass tolerances for precursor and fragment ions were determined automatically by DIA-NN; retention time prediction type is dynamic iRT, interference on MS2 level correction is enabled and cross run normalization is enabled, max miss cleavage site:2. Fixed modification is carbamidomethyl(C), dynamic modification is oxidation(M) and acetyl (Protein N-term). All reported data were based on 99% confidence for protein identification as determined by false discovery rate ≤ 1%. Proteins exhibiting higher abundance in flag empty vector control sample were excluded. Differentially expressed proteins were defined as those with ARF1-C159A/ARF1 fold change ≥1.2 or ≤0.83 and *p*-value ≤0.05. R software (version 4.4.3) was used to perform hierarchical clustering analysis and generate volcano plots for differentially expressed proteins, also potential biological functions were annotated by Gene Ontology.

### ARF1 GTPase Activation Assay

The assay to evaluate the level of GTP-ARF1 was performed as described previously (45). The ARF1 activity (GTP-ARF1) in cells and tissues was determined using the ARF1 activation assay kit (ab211170, Abcam) following the manufacturer's instruction. GGA3 Protein-binding domain Agarose beads (GGA3 Protein-binding domain) were used to pull-down the active form of ARF1 from cell and tissue lysates, and GTP-ARF1 in the immunoprecipitate was analyzed by Western blotting.

### Co-immunoprecipitation

HEK-293T cells plated in 10-cm dishes were transfected with the indicated Flag-tagged plasmid for 36 to 48 h, the cells were washed with PBS, collected in RIPA Lysis Buffer (P0013k, Beyotime) with Protease inhibitor cocktail (P1006, Beyotime). Following 30 min on ice, lysates were spun at 14,000 rpm for 10 min at 4 °C. 1 ml aliquots at 1 mg/ml of lysate were incubated with Anti-Flag Affinity Resin (101274-MM13-RN, Sino Biological) overnight at 4 °C. Beads were subsequently washed three times with PBS and processed for SDS-PAGE and immunoblot.

### Plasma Membrane Protein Isolation and Cell Fractionation

HEK-293T cells cultured in 10-cm dishes were transfected with ARF1-Flag and then collected for plasma membrane protein isolation (Invent Biotechnologies, SM-005) according to the manufacturer's instructions. Briefly, cells were first sensitized by lysis buffer and then pass through the filter with high-speed centrifugation (16,000 *g*, 30 s), resulting in a cell lysate containing ruptured cell membranes and intact nuclei. After removing the nuclear pellet, Cytosol fraction, PM and organelle fraction were sequentially separated from the cell lysate by differential and density centrifugation (46).

### OptiPrep Gradient Fractionation

OptiPrep gradient fractionation was performed as described previously (47). Briefly, seven gradient iodixanol solutions (5%, 10%, 15%, 20%, 25%, 30%, and 35%) were prepared by mixing dilution buffer with 40% (wt/vol) iodixanol. Then, the gradient solution was slowly added to the centrifuge tube in descending order of concentration. Cultured cells were rinsed with PBS twice and Solution A (0.25 M sucrose, 140 mM NaCl, 20 mM Tris·HCl pH 8.0) once. Solution B (0.25 M sucrose, 140 mM NaCl, 1 mM EDTA, 20 mM Tris·HCl pH 8.0) was added to collect the cells, which were then treated by freeze/thaw cycle three times in liquid nitrogen. Cell nuclei were pelleted by centrifugation at 800 *g*, 4 °C for 5 min, and the post-nuclear supernatant was retained. The post-nuclear supernatant sample was placed on top of the iodixanol gradient and centrifuged at 100,000 *g* for 18 h without acceleration/deacceleration (Thermo Fisher Scientific Sorvall WX + series, swinging bucket Rotor TH-641). After centrifugation, 14 fractions were collected for Western blotting.

### Cycloheximide Treatment

Cycloheximide (100 μM; Sigma, #C7698) was incubated with cultured cells for treatment and for various periods of time (*e.g.,* 0–10 h), then the treated cells were harvested and processed for Western blotting analysis (5).

### Cell Counting Kit-8 Assay

Cells were plated in a 96-well plate at a density of 10,000 cells per well and cell proliferation was assessed with a Cell Counting Kit-8 (IV08-1000T, Invigentech) at indicated time point post transfection following the manufacturer’s instruction.

### Molecular Dynamic Simulation

To evaluate the effect of palmitoylation of ARF1 C159, three structure models of palmitoylated ARF1 (apo, GDP-bound, GTP-bound) were generated based on the crystal structure of ARF1(PDB ID: 7DN8 and 2J59), and a 15-ns molecular dynamic simulation was performed on these structures using GROMACS (48) to access whether the palmitoylation of ARF1 C159 affects the binding of GTP/GDP with ARF1. The ARF1 structures were optimized by molecular dynamics simulations using GROMACS. The molecules were solvated with TIP3P water in a cubic box, where the minimal distance between the atoms in the complex and the edges of the box was set to 12 Å. A switch distance of 10 Å and a cutoff distance of 12 Å were used for nonbonded interactions. Particle mesh Ewald was chosen to calculate the long-range electronic interactions. The simulations were conducted at 310 K and a constant pressure of 1 bar. They were first minimized for 10,000 steps and then equilibrated in the isothermal-isobaric ensemble for 15 ns, with an integration time step of 1 fs.

### Experimental Design and Statistical Rationale

This study utilized MS-based proteomics to identify the specific cysteine residue within ARF1 that undergoes palmitoylation. This modification was initially detected *via* MS (Supplemental Table S3) and subsequently validated through complementary biochemical assays. Additionally, MS-based proteomics was employed to characterize proteins interacting with both WT ARF1 and the ARF1-C159A mutant. To ensure reproducibility and statistical reliability, this experiment was performed with three biological replicates, derived from independent immunoprecipitation samples. Basic descriptive data are presented as means standard errors of means. For statistical analyses of differences between two groups, paired or unpaired two-tailed Student’s t-tests were used where appropriate. For experiments involving more than two groups, the one-way ANOVA analysis was carried out. Post-hoc pairwise comparisons, with Bonferroni correction for multiple comparisons, were conducted where appropriate. An α-level of 0.05 was adopted in all instances. All analyses were carried out using SPSS 19 professional software (IBM). Graphs were created using GraphPad Prism (Windows, version 7). None of the samples were excluded from the statistical analysis. Sample sizes referred to the general application of the field and were not statistically predicted.

## Results

### ARF1 is a Palmitoylated Protein

**Fig. 1:**
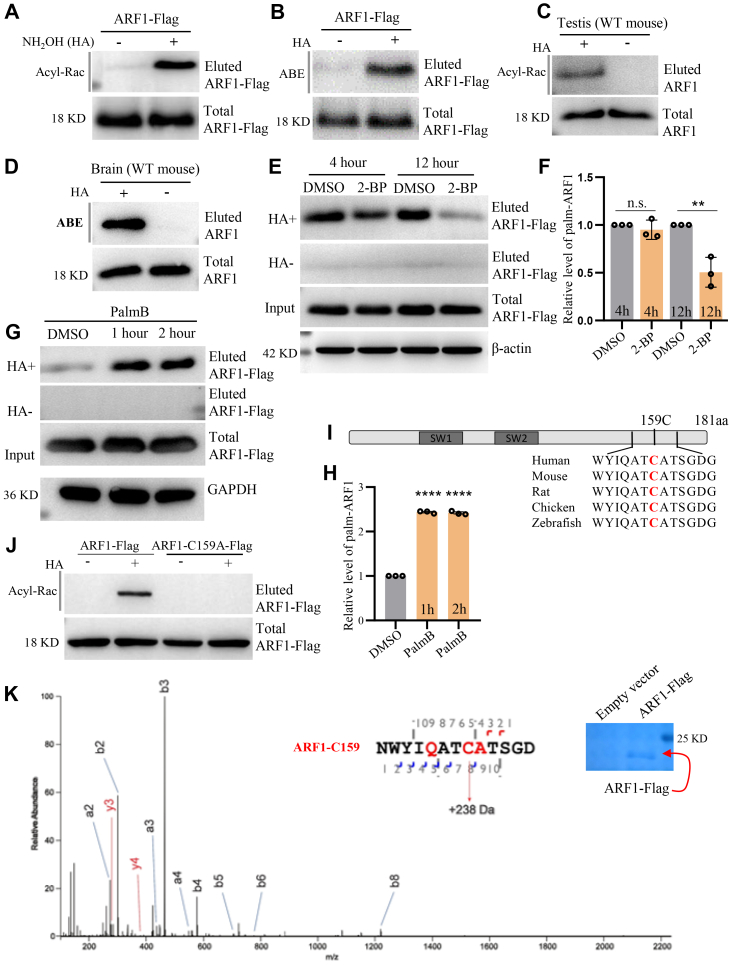
**ARF1 is a palmitoylated protein.***A* and *B*, ARF1-Flag was expressed in HEK-293T cells and the lysate was subjected to Acyl-Rac and ABE assays to detect ARF1 palmitoylation. *C* and *D*, testis or brain lysate from WT mouse was subjected to Acyl-Rac or ABE assay to detect ARF1 palmitoylation. *E* and *F*, HEK-293T cells expressing ARF1-Flag were treated with DMSO or 2-BP (50 μM) for varied periods and examined by Acyl-Rac to detect levels of ARF1 palmitoylation, the results were quantified. Treatment of 4 h, *p* = 0.4399; Treatment of 12 h, ∗∗*p* = 0.0053, unpaired *t* test (2-tailed, n = 3 repeats). *G* and *H*, HEK-293T cells expressing ARF1-Flag were treated with DMSO or palmostatin B (1 μM) for a varied period and examined by Acyl-Rac to detect levels of ARF1 palmitoylation, the results were quantified. ∗∗∗∗*p* < 0.0001, one-way ANOVA; Bonferroni post hoc test comparing DMSO and palmostatin B (1 h or 2 h), n = 3 repeats. *I*, partial protein sequences of ARF1 from different species was aligned to assess cysteine conservation. *J*, WT ARF1 and its mutant ARF1-C159A were expressed in HEK-293T cells and examined by Acyl-Rac to detect ARF1 palmitoylation. *K*, purified ARF1 was probed by mass-spectrometry, a mass-shift of 238 Da linked to cysteine is a hallmark for palmitoylation. Data are mean ± s.e.m. ABE, Acyl-Biotin Exchange.

Initially, to test if ARF1 is truly palmitoylated, HEK-293T cells expressing ARF1-Flag or endogenously expressed ARF1 in testis/brain were subjected to ABE and Acyl-Rac assays (Supplemental Fig. S1*A*), which evaluate the status of palmitoylation/S-acylation of target proteins. The results showed that ARF1 is readily palmitoylated (Fig. 1, *A*–*D*, and Supplemental Fig. S8). For confirmation, 2-BP, a non-specific inhibitor of protein palmitoylation (49, 50), and palmostatin B (PalmB), an inhibitor of depalmitoylation (51, 52), were used to incubate with HEK-293T cells expressing ARF1-Flag. The results of Acyl-Rac showed that 2-BP treatment for 12 h could significantly decrease the level of palmitoylated ARF1 (palm-ARF1) (Fig. 1, *E* and *F* and Supplemental Fig. S8), while the treatment of PalmB dramatically elevates the level of palm-ARF1 (Fig. 1, *G* and *H* and Supplemental Fig. S8), indicating that ARF1 palmitoylation can be dynamically modulated. To determine the palmitoylated cysteine residue in ARF1, sequence alignment of ARF1 proteins from different species was carried out. The analysis revealed that ARF1 has only one cysteine residue, which is likely the site where palmitoylation occurs (Fig. 1*I*). Therefore, an ARF1 mutant (Cys159 to Alanine, ARF1-C159A) was generated for the Acyl-Rac analysis. The result showed that C159A completely abolishes the palmitoylation of ARF1(Fig. 1*J* and Supplemental Fig. S8), verifying that C159 is the site of palmitoylation in ARF1. For direct validation, ectopically-expressed ARF1-Flag was purified and subjected to mass-spectrometry analysis, and the result showed that C159 in ARF1 is modified by palmitoylation (with 238 Da of mass alteration (5)) (Fig. 1*K*).

### Blocking ARF1 Palmitoylation Enhances its Localization at Golgi

**Fig. 2:**
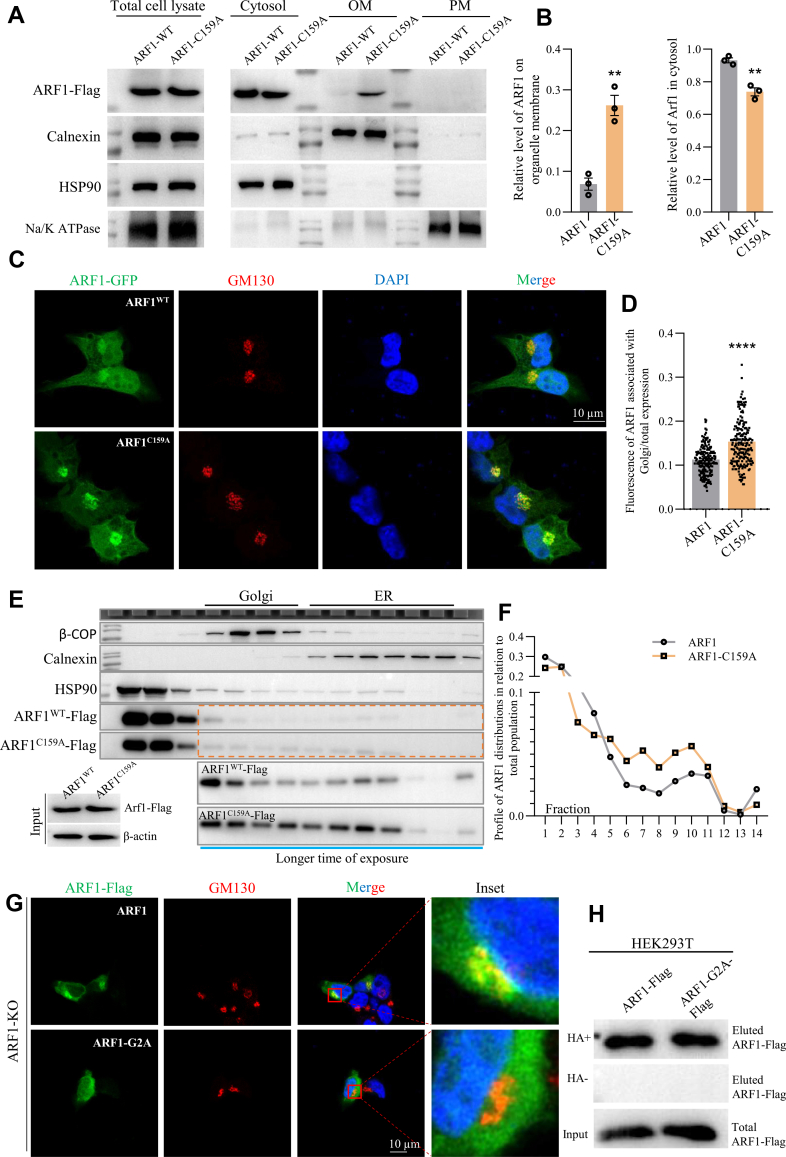
**Blocking ARF1 Palmitoylation enhances its localization at Golgi.***A,* HEK-293T cells expressing WT ARF1 or ARF1-C159A were subjected to subcellular compartment (*i.e.,* cytosol and organelle membrane) fractionation and examined by Western blots. The levels of ARF1 and ARF1-C159A in different fractions were quantified. *B*, ∗∗*p* = 0.0051 (organelle membrane), ∗∗*p* = 0.0055 (Cytosol), unpaired *t* test, 2-tailed, n = 3 repeats. *C* and *D*, ARF1-GFP or ARF1-C159A-GFP were cotransfected in HEK-293T cells for the measurement of their subcellular localizations (*C*), and ARF1 fluorescence associated with Golgi in relation to total expression was quantified (*D*). ∗∗∗∗*p* < 0.0001, unpaired *t* test, 2-tailed, n = 201 to 202 cells from 3 repeats. *E* and *F*, ARF1 or ARF1-C159A were expressed in HEK-293T cells, the post-nuclear homogenates were subjected to fractionation in 5 to 35% OptiPrep gradients (*E*), and ARF1 distribution in ER and Golgi fractions in relation to total ARF1 was profiled (*F*). *G*, ARF1 or ARF1-G2A were expressed in ARF1-KO HEK-293T cells for immunofluorescence imaging. *H*,ARF1 or ARF1-G2A were expressed in HEK-293T cells and the lysate was subjected to ABE assay to detect ARF1 palmitoylation. Data are mean ± s.e.m. ABE, Acyl-Biotin Exchange.

To explore whether palmitoylation might be involved in regulating ARF1 subcellular localization, HEK-293T cells expressing ARF1 or ARF1-C159A were subjected to cytosol/plasma membrane and cytosol/organelle membrane fractionations. The results showed that the localization of ARF1 at the plasma membrane is minimal for both ARF1 and ARF1-C159A, while the level of ARF1-C159A localized to the organelle membrane is significantly upregulated as compared with that of WT ARF1, and the levels of total proteins are almost equal (Fig. 2, *A* and *B* and Supplemental Fig. S9). To identify the localization that ARF1-C159A might retain in cells, ARF1-GFP or ARF1-C159A-GFP were expressed. The results showed that both proteins localize not only in the cytosol but also at the Golgi apparatus (GM130), interestingly, ARF1-C159A-GFP localizes more abundantly at Golgi than that of the ARF1-GFP (Fig. 2, *C* and *D*), which is consistent with the observation in Figure 2, *A* and *B*. Moreover, the subcellular components isolated from the post-nuclear homogenates using OptiPrep gradients showed that more ARF1-C159A localized to the Golgi and part of the ER as compared with that of the WT ARF1 (Fig. 2, *E* and *F* and Supplemental Fig. S9). Together, these experiments indicated that blocking ARF1 palmitoylation promotes its localization at Golgi. Additionally, an estimate of the stoichiometry of ARF1 palmitoylation revealed that about 10% of ARF1 is palmitoylated (Supplemental Fig. S1, *D* and *E*), and palmitoylation is not required for ARF1 protein stability (Supplemental Fig. S1, *B* and *C*).

Previous studies have identified N-terminal myristoylation at Gly2 as the primary determinant for Golgi targeting of ARF1(22). To investigate whether crosstalk exists between myristoylation and palmitoylation, we constructed an ARF1 G2A mutant. Our results demonstrate that the G2A mutation completely abolishes ARF1 Golgi localization (Fig. 2*G*). However, this mutation does not interfere with its palmitoylation status (Fig. 2*H*), suggesting that these two lipid modifications likely regulate distinct molecular processes.

### Identifying the Enzymes that May Catalyze Dynamic ARF1 Palmitoylation

**Fig. 3:**
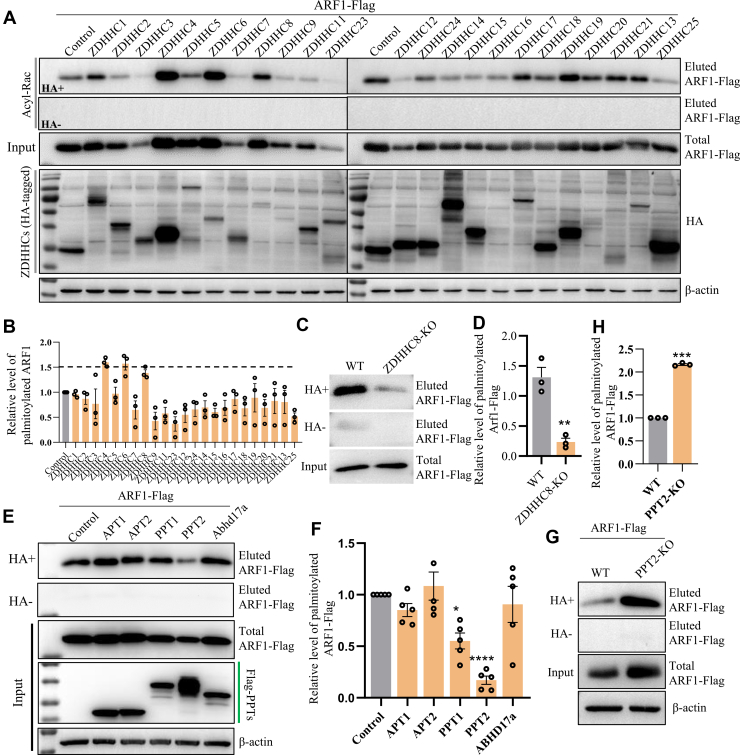
**Identifying the enzymes that regulate dynamic ARF1 palmitoylation.***A* and *B*, different ZDHHCs were coexpressed with ARF1-Flag in HEK-293T cells and processed with Acyl-Rac assay for the detection of the level of ARF1 palmitoylation, the results were quantified. *p* > 0.05, one-way ANOVA; Bonferroni post hoc test between Control and ZDHHCs, n = 3 repeats. *C* and *D*, ARF1-Flag was expressed in either WT or ZDHHC8-KO HEK-293T cells, and subjected to Acyl-Rac assay, the results were quantified, ∗∗*p* = 0.0036, unpaired *t* test, 2-tailed, n = 3). *E* and *F*, varied PPTs were coexpressed with ARF1-Flag in HEK-293T cells and processed with Acyl-Rac assay for the detection of the level of ARF1 palmitoylation, and quantified. One-way ANOVA; Bonferroni post hoc test comparing control and PPT2, ∗∗∗∗*p* < 0.0001; Control *versus* PPT1, ∗*p* = 0.0216, n = 5 repeats. *G* and *H*, ARF1-Flag was expressed in either WT or PPT2-KO HEK-293T cells, and subjected to Acyl-Rac assay (*G*), the relative level of ARF1 palmitoylation was quantified (*H*). ∗∗∗*p* = 0.0003, unpaired *t* test, 2-tailed, n = 3 repeats. Data are mean ± s.e.m.

As shown above, ARF1 palmitoylation can be dynamically modulated by 2-BP or PalmB (Fig. 1, *C* and *E*), we then thought to identify the enzymes catalyzing ARF1 palmitoylation and depalmitoylation. On one hand, a study involved co-expressing a panel of mouse ZDHHCs with ARF1-Flag, and the Acyl-Rac results revealed that none of the ZDHHCs significantly increased palm-ARF1 levels statistically. However, ZDHHC-4, -6, and -8 were able to moderately enhance the level of palm-ARF1 compared to the control (Fig. 3, *A* and *B* and Supplemental Fig. S10). For confirmation, ZDHHC8 was depleted in HEK-293T cells (Supplemental Fig. S4, *A*–*C*) and the Acyl-Rac results showed that the level of ARF1 palmitoylation is significantly downregulated in ZDHHC8-KO cells as compared with that of the WT cells (Fig. 3, *C* and *D* and Supplemental Fig. S10). On the other hand, different PPTs were co-expressed with ARF1-Flag, and the experiments showed that both PPT1 and PPT2 could significantly reduce the level of palm-ARF1, but PPT2 is more efficient (Fig. 3, *E* and *F*, Supplemental Figs. S1*F*, and S10). For verification, ARF1-Flag was expressd in WT or PPT2-KO HEK-293T cells (Supplemental Fig. S5, *A*–*C*), and the data illustrated that the level of palm-ARF1 is significantly augmented in PPT2-KO as compared with that in the WT cells (Fig. 3, *G* and *H* and Supplemental Fig. S10).

To investigate the potential interaction between PPT2 and ARF1, ARF1-GFP and RFP-PPT2/PPT2-RFP were co-expressed in HEK-293T cells for live cell imaging, with LysoViewTM633 added to visualize lysosomes. The imaging data revealed two populations of PPT2, one colocalizing with lysosomes and the other lysosome-independent (Supplemental Fig. S1, *A* and *B*). Interestingly, both populations were found to colocalize with ARF1 (Supplemental Fig. S2*A*). Additionally, the cycloheximide treatment assay demonstrated that the ARF1-C159A mutant did not accelerate the protein degradation of ARF1 (Supplemental Fig. S1, *B* and *C*), suggesting that PPT2-mediated ARF1 depalmitoylation is not targeted for protein degradation.

### ARF1 Activation is Reversibly Regulated by its Palmitoylation

**Fig. 4:**
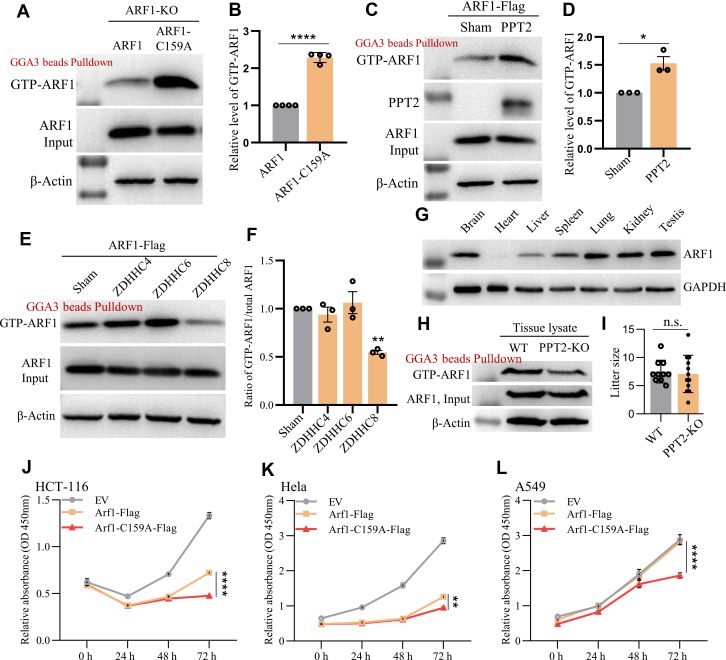
**The active state of ARF1 is modulated by its palmitoylation.***A* and *B*, WT ARF1 or its mutant ARF1-C159A were expressed in ARF1-KO HEK-293T cells respectively, the lysate of which was pulled down by GGA3 beads to detect GTP-ARF1 (*A*) and quantified (*B*). ∗∗∗∗*p* < 0.0001, unpaired *t* test, 2-tailed, n = 4 repeats. *C* and *D*, ARF1-Flag was coexpressed with either empty vector (sham) or PPT2 in ARF1-KO HEK-293T cells, their lysates were pulled down by GGA3 beads to detect GTP-ARF1 (*C*) and quantified (*D*). ∗*p* = 0.0119, unpaired *t* test, 2-tailed, n = 3 repeats. *E* and *F*, ARF1-Flag was coexpressed with either empty vector or ZDHHCs (−4, −6, −8) in ARF1-KO HEK-293T cells and their lysates were pulled down by GGA3 beads to detect GTP-ARF1 (*E*), the results were quantified (*F*). ∗∗*p* = 0.0045, one-way ANOVA; Bonferroni post hoc test comparing Sham and ZDHHC8, n = 3 repeats. *G*, the protein level of ARF1 was detected in different organs of WT mice. *H*, the testis lysate of WT or PPT2-KO mice was pulled down by GGA3 beads to detect GTP-ARF1. *I*, the litter size of WT and PPT2-KO mice was quantified by homozygotes mating. *p* = 0.7211, unpaired *t* test, 2-tailed, n = 10 to 12. *J* and *L*, HCT-116, HeLa, and A549 cells expressing ARF1 or ARF1-C159A were subjected for Cell Counting Kit-8 assay, and quantified. n = 3 repeats, one-way ANOVA; ∗∗*p* < 0.01, ∗∗∗∗*p* < 0.0001. Data are mean ± s.e.m.

Since ARF1 is a small GTPase, cycling between inactive (GDP-bound) and active (GTP-bound) states, we thought to investigate whether palmitoylation might interfere with its activation. To such end, ARF1 or ARF1-C159A was expressed in ARF1-KO cells (Supplemental Fig. S6, *A*–*C*) and their lysates were pulled down by GGA3 beads, which bind only to ARF1 in the active state (GTP-ARF1) (45). Surprisingly, the experiment presented that the level of active ARF1 was higher in cells expressing ARF1-C159A as compared with that of WT ARF1 (Fig. 4, *A* and *B* and Supplemental Fig. S11), indicating that blocking palmitoylation somehow impacted ARF1 GTPase activity. To confirm this observation, ARF1-Flag was co-expressed with PPT2 in HEK-293 cells. The results showed that decreasing palm-ARF1 (Fig. 3, *E* and *F*) leads to a higher level of active ARF1 as compared with the control (Fig. 4, *C* and *D* and Supplemental Fig. S11). Also, we investigated whether the augmented level of palm-ARF1 by the expression of ZDHHCs could reduce the level of GTP-ARF1. Interestingly, only co-expression with ZDHHC8, but not ZDHHC4/6, reduced the level of GTP-ARF1 (Fig. 4, *E* and *F* and Supplemental Fig. S11), supporting the notion that the dynamics of palm-ARF1 could alter the activation of ARF1 bidirectionally. Physiologically, ARF1 is abundantly expressed in many tissues in WT mice, including the testis (Fig. 4*G* and Supplemental Fig. S11). Therefore, the testis lysate of WT or PPT2-KO mice (Supplemental Fig. S7, *A*–*C*) was collected for the quantitation of GTP-ARF1, and the data showed that deletion of PPT2 increases palm-ARF1 (Supplemental Fig. S7*D*) whereas decreasing the level of GTP-ARF1 (Fig. 4*H* and Supplemental Fig. S11), which has no obvious effect on the fertility of PPT2-KO (Fig. 4*I*). As PPT2-KO causes severe neurodegenerative diseases (53, 54), the potential pathological roles of ARF1 palmitoylation in CNS have not been explored here, which invites further investigations.

To further confirm the role of palmitoylation in regulating ARF1 activation, ARF1 and ARF1-C159A were expressed in various CCL, taking into account previous findings implicating ARF1 in the control of cell proliferation (55). The results showed that ARF1 expression decreased cell proliferation in HCT-116 and HeLa cells, but not in A-549 cells. Interestingly, the expression of ARF1-C159A significantly enhanced (at 72 h time points) this effect in all cell types compared to the WT control, indicating that blocking ARF1 palmitoylation by the C159A mutation increases its activation (Fig. 4, *J*–*L*). This provides additional evidence supporting the role of palmitoylation in the regulation of ARF1 activity.

### ARF1 Palmitoylation is Involved in the Retrograde Transport via Interacting with β-COP and β-actin

**Fig. 5:**
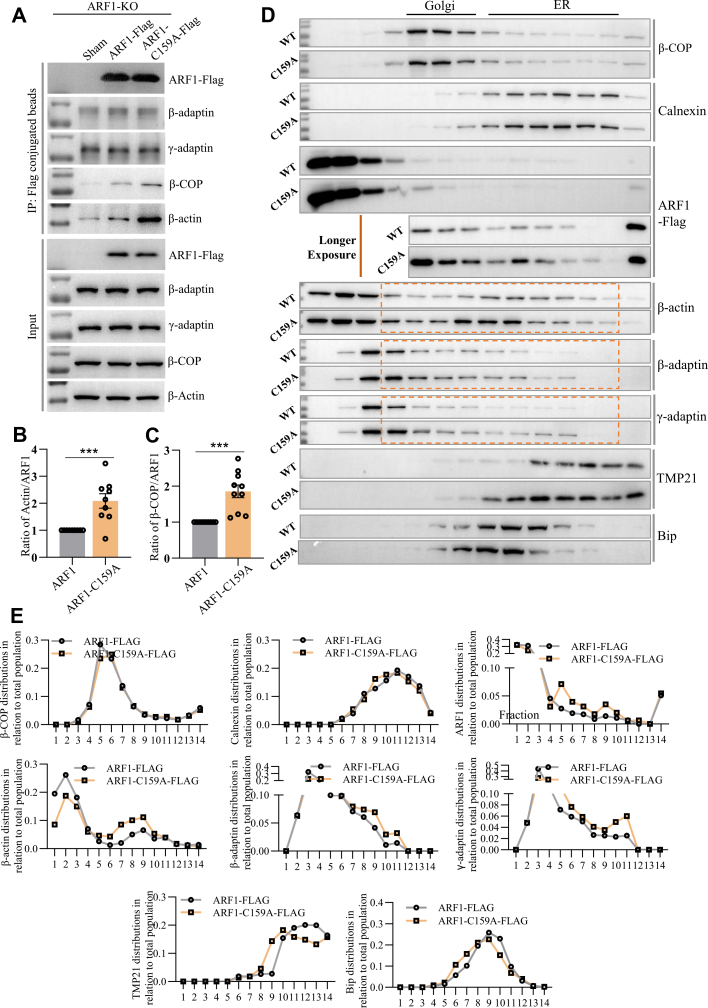
**ARF1 palmitoylation might be involved in modulating the retrograde transport *via* interacting with β-COP and β;-actin.***A*, Empty vector (Sham), ARF1-Flag and ARF1-C159A-Flag were expressed in ARF1-KO HEK-293T cells, lysates of which were pulled down by Flag antibody conjugated beads to examine potential binding partners. *B* and *C*, The levels of β-actin (*B*) and β-COP (*C*) associated with ARF1 were quantified. ∗∗∗*p* = 0.0009, ∗∗∗∗*p* < 0.0001, unpaired *t* test, 2-tailed, n = 9 repeats. *D* and *E*, ARF1 and ARF1-C159A were expressed in ARF1-KO HEK-293T cells, and the post-nuclear homogenates were fractionated in 5 to 35% OptiPrep gradients and analyzed by Western blots to detect protein distributions (*D*), and protein distributions in relation to total population were profiled (*E*). Data are mean ± s.e.m.

Considering that ARF1 palmitoylation negatively regulates its activation, we wondered if the non-palmitoylated ARF1 (ARF1-C159A), with increased GTP-ARF1 level (Fig. 4, *A* and *B*), might manipulate the retrograde transport, *e.g.,* from Golgi to ER. To test this assumption, we examined the binding affinity of ARF1 or ARF1-C159A with its known interactors including β-adaptin, γ-adaptin, β-actin, and β-COP that are responsible for retrograde transportation (11, 14, 56, 57). The results showed that the association of β-adaptin/γ-adaptin with ARF1/ARF1-C159A was not apparently altered, however, the association of β-COP and β-actin with ARF1-C159A is significantly enhanced as compared with WT ARF1 (Fig. 5, *A*–*C* and Supplemental Fig. S12), indicating that enhanced activation of ARF1 promotes the recruitment of β-COP and β-actin. For confirmation, the post-nuclear homogenate was separated by OptiPrep gradients to map the distribution of subcellular components (Fig. 5, *D* and *E* and Supplemental Fig. S12), and the results showed the amount of β-actin distributed in ER/Golgi fractions increases in cells expressing ARF1-C159A, compared with WT ARF1. Similarly, the distributions of β-adaptin and γ-adaptin are also increased in the ER/Golgi fractions in ARF1-C159A expressing cells, supporting the idea that the activity of the retrograde transport machinery is augmented upon the inhibition of ARF1 palmitoylation.

Notably, it was reported that the activation of ARFs further stabilizes its association with the ER/Golgi membrane (24, 58), in agreement, here we showed that ARF1-C159A localizes more in the fractions of ER/Golgi than that of the WT ARF1 (Figs. 2, *E* and *F* and 5, *D* and *E*). Moreover, as a consequence, the distributions of TMP21 (chaperone of ARF1 to recruit vesicles) (12, 36) and Bip (the potential cargos of the retrograde transportation from Golgi to ER) (37) are also modulated. Yet, the distributions of Calnexin and β-COP are not that much affected across various fractions (Fig. 5, *D* and *E*).

Reversely, an augmented level of ARF1 palmitoylation may suppress the retrograde transport machinery. To this end, ARF1-Flag is expressed in WT and PPT2-KO cells, where the level of ARF1 palmitoylation is elevated (Fig. 3, *E* and *F*). The IP results showed that the association of β-COP or β-actin with ARF1-Flag is significantly downregulated in PPT2-KO as compared with that of WT cells (Supplemental Fig. S3, *A–C*). Accordingly, the OptiPrep gradients experiment showed that the distributions of β-actin, β-adaptin and γ-adaptin are weakened in the ER/Golgi fractions of PPT2-KO as compared with that of the WT control (Supplemental Fig. S3, *D* and *E*). Consequently, the distributions of ARF1 and Calnexin were compromised in the ER fractions (Supplemental Fig. S3, *D* and *E*). Together, this evidence strengthened the notion that reversible ARF1 palmitoylation dynamically regulates ARF1 activity, which impacts its localization at the Golgi membrane, and modulates the retrograde transport.

**Fig. 6:**
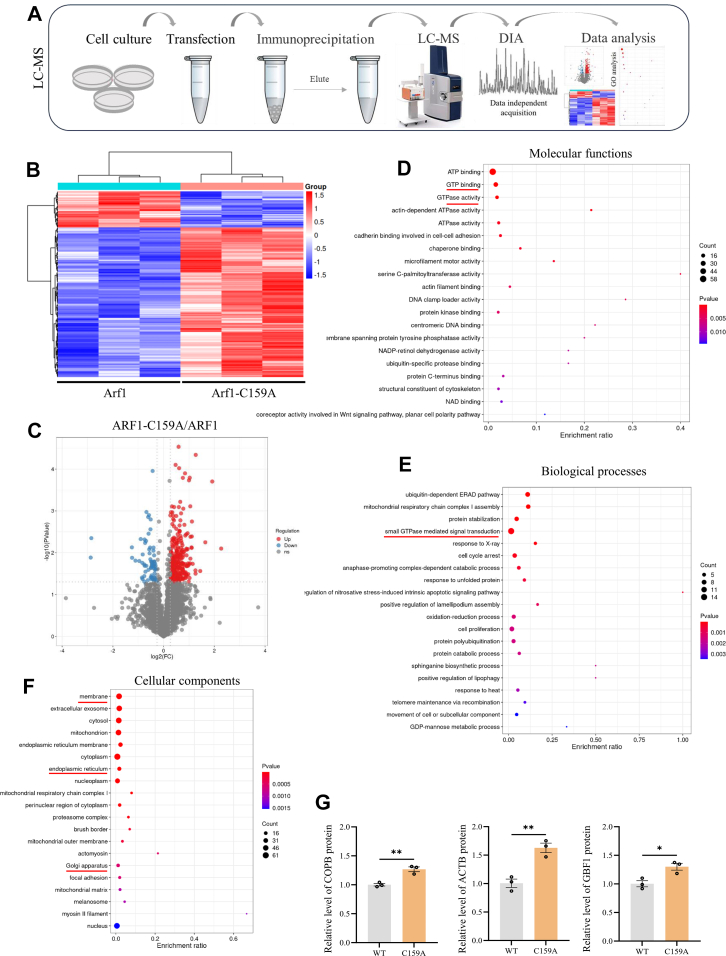
**Affinity purification–mass spectrometry analysis of ARF1 interactomes.***A*, schematic of the affinity purification–mass spectrometry experimental workflow. *B* and *C*, differentially enriched proteins identified in HEK-293T ARF1-KO cells expressing ARF1-C159A compared to WT ARF1. *D and F*, gene Ontology analysis of differentially enriched proteins: (*D*) molecular functions, (*E*) biological processes, (*F*) cellular components. *G*, quantification of expression levels for selected proteins in HEK-293T ARF1-KO cells expressing ARF1-C159A or WT ARF1. Data represent mean ± s.e.m.

For further validation, affinity purification–mass spectrometry was employed to compare the interactomes of WT and C159A ARF1 (Fig. 6, *A*–*C*). The C159A mutation significantly altered molecular processes related to GTP binding, GTPase activity (Fig. 6*D*), and GTPase-associated signaling pathways (Fig. 6*E*) within membrane components (Fig. 6*F*). Key altered interactors included COPB and ACTB, findings fully consistent with the data in Figure 5. This proteomic analysis substantiates and extends the mechanistic conclusions of our study.

### Predicted Mechanism of Increased GTP Binding to ARF1 upon Blocking its Palmitoylation Site

**Fig. 7:**
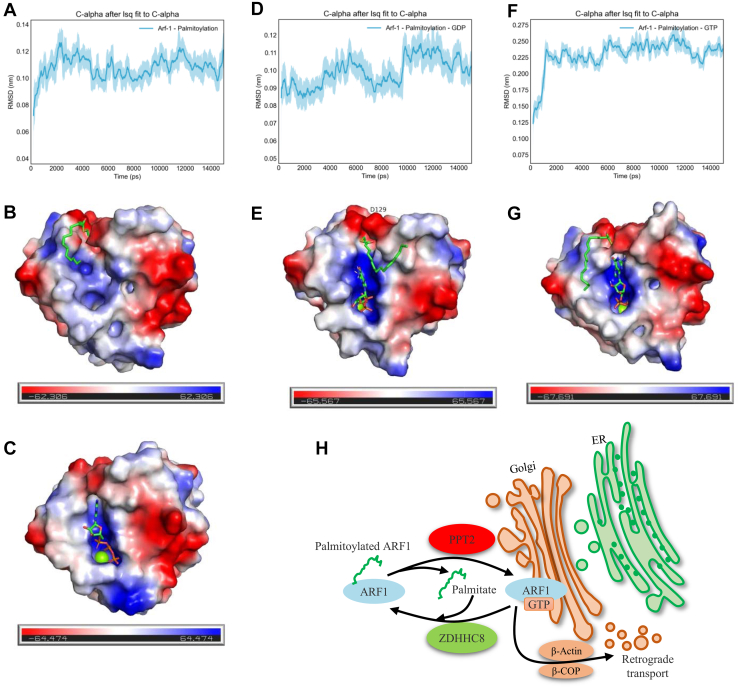
**The palmitoylation of ARF1 C159 blocks the binding of GTP/GDP with apo-ARF1.***A* and *B*, the equilibrium conformation of palmitoylated apo-ARF-1, in which the fatty acid chain of palmitic acid is likely to occupy the groove for GTP/GDP. *A*, the process of dynamic simulation. *B*, the structure model of palmitoylated apo-ARF-1. *C*, the structure model of GTP-bound ARF1. In these figures, ARF1 is shown in surface mode. The palmitic acid, GDP, and GTP are shown in stick mode. The Mg^2+^ is shown in sphere mode. *D* and *E*, the equilibrium conformation of palmitoylated GDP-bound ARF1, in which the fatty acid chain of palmitic acid is likely to insert into the groove for GTP/GDP and disturb the bind of GDP with ARF1. *D*, the process of dynamic simulation. *E*, the structure model of palmitoylated GDP-bound ARF1. *F* and *G*, the equilibrium conformation of palmitoylated GTP-bound ARF1, in which the fatty acid chain of palmitic acid is likely to swing outside of the groove for GTP/GDP. In this conformation, the palmitic acid tail does not disturb the binding of GTP with ARF1. *F*, the process of dynamic simulation. *G*, the structure model of palmitoylated GTP-bound ARF1. *H*, schematic diagram of ARF1 activation controlled by its dynamic palmitoylation, in which reversible ARF1 palmitoylation is catalyzed by ZDHHC8 and PPT2, reduced level of ARF1 palmitoylation enhances its activation and the retrograde (Golgi to ER) transport.

We then investigated how palmitoylation on C159 affects the nucleotide-binding state of ARF1. To do this, we generated three structure models of palmitoylated ARF1 (apo, GDP-bound, GTP-bound) based on the crystal structure of ARF1 (PDB ID: 7DN8 and 2J59) (13). We then conducted molecular dynamics (MD) simulations on these structures using GROMACS to determine whether palmitoylation of ARF1 C159 impacts the binding of GTP/GDP to ARF1. The results of the MD simulations revealed that the fatty acid chain of palmitic acid is likely to occupy the groove for GTP/GDP in apo-ARF1 (Fig. 7, *A*–*C*). Intriguingly, in GDP-bound ARF1, the fatty acid chain of palmitic acid still tends to reside in the groove, potentially interfering with the binding of GDP and inhibiting the exchange of GDP to GTP for ARF1 activation (Fig. 7, *D* and *E*). However, in GTP-bound ARF1, the palmitic acid tail does not disrupt the binding of GTP to ARF1 (Fig. 7, *F* and *G*). These findings suggest that palmitoylation of ARF1 on C159 may influence the binding of GDP by occupying the GTP/GDP groove with the fatty acid chain, thereby impairing the activation (exchange of GDP to GTP) of ARF1. This sheds light on a potential mechanism by which palmitoylation regulates ARF1 activity.

## Discussion

The discovery of ARF1 as a palmitoylated protein (Fig. 1) has provided insight into the involvement of ZDHHC8 and PPT1/2 in regulating the dynamic nature of ARF1 palmitoylation (Fig. 3). Further exploration has led to the understanding that ARF1 palmitoylation plays a significant role in modulating its activation (Fig. 4), thereby influencing its subcellular localization and retrograde transportation (Figs. 2 and 5). Through a combination of biochemical analysis and MD simulation, we have proposed a mechanism suggesting that ARF1 palmitoylation may serve as a dynamic regulatory switch for ARF1 activation (Fig. 7H).

An analysis of ARF1 palmitoylation estimates that approximately 10% of ARF1 is palmitoylated (Supplemental Fig. S1, *C* and *D*). Additionally, it was observed that around 10% of the total ARF1 is linked to the Golgi (Fig. 2, *C* and *D*), while the majority of ARF1 is localized in the cytosol (Fig. 2, *A* and *B*). Surprisingly, blocking ARF1 palmitoylation (ARF1-C159A) notably increases its association with the Golgi (Fig. 2, *C* and *D*), which appears contradictory to previous findings suggesting that palmitoylation generally facilitates subcellular membrane associations (3, 40, 59). However, previous studies have highlighted the significance of the N-terminal amphipathic helix and myristoylation of ARF1 for its residence at the Golgi (22, 57, 60), implying that palmitoylation may regulate ARF1 for functions such as activation. Indeed, it has been demonstrated that inhibiting ARF1 palmitoylation (ARF1-C159A) significantly elevates the level of GTP-ARF1 (Fig. 4), which aligns with previous findings indicating that the activation of ARFs involves a conformational change, bringing them into close contact with the membrane (23, 25). Taken together, these data suggest the possibility that palmitoylation may not directly regulate the membrane association of ARF1; instead, blocking ARF1 palmitoylation enhances its activation, thereby increasing its association with the Golgi.

It is important to note that cysteine 159 is located in the guanine nucleotide-binding pocket of ARF1. Mutation of cysteine 159 to alanine (C159A) may lead to alterations in nucleotide exchange rates and potentially hydrolysis. However, additional experiments have shown that the co-expression of PPT2/ZDHHC8 with WT ARF1 significantly influences the level of GTP-bound ARF1 (Fig. 4, *C*–*F*), suggesting that palmitoylation likely plays a role in regulating ARF1 activation. Notably, molecular dynamics simulations suggest that palmitoylation at cysteine 159 could impact GDP binding, potentially hindering the exchange of GDP to GTP for ARF1 activation (Fig. 7, *A*–*G*). Jointly, these evidences suggest a mechanistic mechanism that palmitoylation primarily inhibits nucleotide exchange rather than other possibilities *e.g.*, directly blocking the interacting interfaces of β-COP. Considering that the activation of ARF1 is intricately controlled by various factors such as GEFs, acidic phospholipids, and Golgi membrane localization (15, 24, 58), the modulation of ARF1 activation through palmitoylation may serve as an additional regulatory mechanism. This emphasizes the importance of finely tuning the dynamic activation of ARF1 on-site to maintain proper Golgi function and beyond. However, cysteine 159 has also been implicated in redox-based regulation (61). This dual regulatory potential complicates data interpretation as C159A mutation is not specific to palmitoylation, as it also ablates the residue's capacity for other modifications and its native functional roles.

During the screening for enzymes responsible for palmitoylating ARF1, the primary candidates identified were ZDHHC4/6/8, suggesting that multiple ZDHHCs could play a role in mediating ARF1 palmitoylation (Fig. 3, *A* and *B*). Growing evidence indicates that while the co-expression of ZDHHC8, rather than ZDHHC4/6, with ARF1 significantly inhibits ARF1 activation (Fig. 4, *E* and *F*), it raises the possibility that different pools of palmitoylated ARF1 may be subject to distinct regulatory mechanisms. Nevertheless, the deletion of ZDHHC8 results in a notable decrease in palm-ARF1 levels in HEK293T cells (Fig. 3, *C* and *D*), suggesting that ZDHHC8 plays a crucial role in catalyzing ARF1 palmitoylation. Despite the presence of a residual amount of palm-ARF1 in ZDHHC8-KO cells (Fig. 3, *C* and *D*), one should not dismiss the likelihood that other ZDHHCs may also be involved in this process.

The screening for enzymes responsible for depalmitoylating ARF1 revealed that PPT1/2 can significantly reduce palm-ARF1 levels, with PPT2 demonstrating greater efficacy in decreasing ARF1 palmitoylation compared to PPT1 (Fig. 3, *E* and *F*). It is worth noting that while PPT2 was initially thought to be catalytically inactive *in vitro* (62), subsequent studies from the same research group demonstrated that the knockout of either PPT1 or PPT2 resulted in similar phenotypes of lysosomal storage disease (53), indicating that both proteins have similar, yet not entirely redundant, molecular functions *in vivo*. Furthermore, PPTs have been reported to target lysosomes (63), and there is evidence suggesting that they can be secreted and taken up by other cells (64), potentially existing in synaptic terminals (65). This raises the intriguing possibility that cytosolic PPT2 may play a role in depalmitoylating ARF1. Supporting this notion, our data revealed that lysosome-independent PPT2 colocalizes with ARF1 (Supplemental Fig. S2, *A* and *B*), indicating that PPT2 could be involved in catalyzing ARF1 depalmitoylation.

Interestingly, β-actin is involved in ARF1-mediated intracellular protein transport, consistent with previous research indicating that ARFs play a crucial role in regulating actin cytoskeleton dynamics possibly through interactions with actin-binding proteins like Cdc42 and ARP2/3 (17, 66, 67).

Finally, it should be noted that other Class I ARFs, such as ARF2 and ARF3, may also undergo palmitoylation and could potentially compensate for ARF1 loss. Future studies should explore these directions to elucidate functional interactions among these proteins.

Our findings demonstrate that ARF1 undergoes a novel post-translational modification, palmitoylation, which forms a new regulatory pathway that negatively impacts its activation and subcellular localization. These insights may offer valuable implications for understanding the regulation of other ARFs or small GTPases, given that RABs occasionally share GAPs with ARFs (68, 69, 70, 71, 72, 73). Furthermore, our data propose a potential new approach to modulate ARF1 function, presenting a promising avenue for developing treatments targeting diseases resulting from aberrant ARF1 activation.

## Data Availability

All data generated in this study are included in the manuscript and supporting files. All raw mass spectrometry data files have been deposited to the [iProX/PRIDE] repository under the dataset identifier PXD072413 (LC-MS for ARF1 interactomes) and PXD072641 (LC-MS for ARF1 palmitoylation). Materials are available from the corresponding author upon reasonable request.

## Supplemental data

This article contains supplemental data.

## Conflict of interest

The authors declare that they have no conflicts of interest with the contents of this article.

## Supplemental Data

Table S3

Binder 2_Supplemental data

Palm-proteomics from WT mouse brain

Supplemental data S2_spectra for SUP
