# Supplementary material for: The Activation of ARF1 Is Dynamically Regulated by its Palmitoylation
Source: Mol Cell Proteomics. 2026 May 14;25(6):101586. doi: 10.1016/j.mcpro.2026.101586 (PMC13273671; doi:10.1016/j.mcpro.2026.101586)
Supplement: Table S1 [file mmc1.docx]

Table S1. Summary of antibodies and dyes in experiments

| **Antibodies** | **Sources** | **Catalogue** | **Host** | **Dilution** |
| --- | --- | --- | --- | --- |
| Anti-Flag | Sigma-Aldrich | F7425 | Rabbit | WB: 1:1000 |
| Anti-HA | Cell Signaling Technology | 2367S | Mouse | WB:1:1000 |
| Anti-ARF1 | Santa Cruz Biotechnology | sc-53168 | Mouse | WB:1:1000 |
| anti DDDDK-Tag | ABclonal | AE024 | Mouse | WB:1:5000 |
| Anti-COPB | Santa Cruz Biotechnology | sc-393615 | Mouse | WB:1:1000 |
| Anti-GM130 | BD Biosciences | 610822 | Mouse | WB:1:500  IF: 1:200 |
| Anti-Calnexin | Santa Cruz Biotechnology | sc-46669 | Mouse | WB:1:1000 |
| Anti-β-actin | ABclonal | AC026 | Rabbit | WB:1:1000 |
| Anti-HSP90 | Cell Signaling Technology | 4874s | Rabbit | WB:1:1000 |
| Anti-TMP21 | Santa Cruz Biotechnology | sc-137003 | Mouse | WB:1:1000 |
| Anti-β-adaptin | Santa Cruz Biotechnology | sc-74423 | Mouse | WB:1:1000 |
| Anti-γ1-adaptin | Santa Cruz Biotechnology | sc-398867 | Mouse | WB:1:1000 |
| Anti-GRP78 BiP | Affinity BioReagents | BF8024 | Mouse | WB:1:1000 |
| Anti-GBF1 | Santa Cruz Biotechnology | sc-136240 | Mouse | WB: 1:1000 |
| Anti-GAPDH | ABclonal | AC033 | Mouse | WB: 1:5000 |
| HRP Goat Anti-Mouse IgG (H+L) | ABclonal | AS003 | Goat | WB: 1:10000 |
| HRP Goat Anti-Rabbit IgG (H+L) | ABclonal | AS014 | Goat | WB: 1:5000 |
| Goat-anti-Rabbit IgG (H+L), Fluor 488 | Invitrogen | A-11034 | Goat | IF:1:1000 |
| LysoView^TM^633 | Biotium | 70058 |  | IF: 1:1000 |
