# Supplementary material for: The Activation of ARF1 Is Dynamically Regulated by its Palmitoylation
Source: Mol Cell Proteomics. 2026 May 14;25(6):101586. doi: 10.1016/j.mcpro.2026.101586 (PMC13273671; doi:10.1016/j.mcpro.2026.101586)
Supplement: Table S3 [file mmc3.docx]

| Peptide sequence (identified S-acylated peptides) | Modification Type(s) | | m/z | | z | | Score | |
| --- | --- | --- | --- | --- | --- | --- | --- | --- |
| R.HRNWYIQATC[+238.230]A.T | C[+238] | | 800.9406 | | 2 | | 79.9 | |
| R.HRNWYIQATC[+238.230]ATS.G | C[+238] | | 596.978 | | 3 | | 2.84 | |
| R.HRN[+0.984]WYIQATC[+238.230]ATSG.D | C[+238], N[+1] | | 923.9754 | | 2 | | 50.56 | |
| R.NWYIQATC[+238.230]ATS.G | C[+238] | | 748.4 | | 2 | | 76.98 | |
| R.N[+0.984]WYIQATC[+238.230]ATS.G | C[+238], N[+1] | | 748.8889 | | 2 | | 7.99 | |
| R.NWYIQATC[+238.230]ATSGD{+421.191}.G | C[+238], D{+421} | | 1045.0165 | | 2 | | 157.54 | |
| R.NWYIQ[+0.984]ATC[+238.230]A{+499.218}TSGD.G | A{+499}, C[+238], Q[+1] | | 1084.5221 | | 2 | | 143.84 | |
| R.NWYIQATC[+238.230]A{+316.009}TSGDGLY[+238.230]E.G | A{+316}, C[+238], Y[+238] | | 1342.6462 | | 2 | | 207.97 | |
| R.NWYIQ[+0.984]ATC[+238.230]ATSGDGLYEGL.D | C[+238], Q[+1] | | 1151.0799 | | 2 | | 28.7 | |
| R.NWYIQATC[+238.230]A{+344.197}TSGDGLYEGLDWLSNQLR.N | A{+344}, C[+238] | | 1219.6242 | | 3 | | 113.57 | |
| R.N[+0.984]WYIQATC[+238.230]ATSGDGLYEGL{+343.201}DWLSNQLR.N | C[+238], L{+343}, N[+1] | | 1219.6202 | | 3 | | 80.4 | |
| N.WYIQATC[+238.230]ATSGD.G | C[+238] | | 518.6021 | | 3 | | 29.39 | |
| N.WYIQATC[+238.230]ATSGD.G | C[+238] | | 777.397 | | 2 | | 50.94 | |
| I.QATC[+238.230]ATSGDGLYE.G | C[+238] | | 777.4002 | | 2 | | 17.72 | |
| I.Q[+0.984]ATC[+238.230]ATSGDGLYE.G | | C[+238], Q[+1] | | 777.8937 | | 2 | | 21.51 |

Table S3. The complete list of S-acylated peptides identified in ARF1 by MS
