# Supplementary material for: The Activation of ARF1 Is Dynamically Regulated by its Palmitoylation
Source: Mol Cell Proteomics. 2026 May 14;25(6):101586. doi: 10.1016/j.mcpro.2026.101586 (PMC13273671; doi:10.1016/j.mcpro.2026.101586)

Supplemental figure legends

Fig. S1. Blocking ARF1 palmitoylation does not affect protein stability. A) Schematic diagram to show the principal of the experimental procedures of Acyl-Rac assay. In brief, free sulfur group is blocked by NEM, palmitoylation site(s) is cleaved by HA (Hydroxylamine) and captured by forming new bond with thiol-reactive sepharose resin. Acyl-Biotin Exchange (ABE) is following a very similar procedure. **B-C**) HEK293T cells expressing ARF1 or ARF1-C159A were treated with CHX (100µM) for varied time periods and examined for the protein level of ARF1 by Western blots, the results were quantified. *P*=0.6704, paired t-test, 2-tailed, n=3. **D-E**) Lysates of HEK293T cells expressing ARF1-Flag (**D**) or mouse brain (**E**) were subjected for ABE assay, same proportion of samples were used for equal loading and side-by-side comparison by Western blots to assess the stoichiometry of ARF1 palmitoylation. **F**) PPT1/2 were coexpressed with ARF1-Flag in HEK293T cells and processed with Acyl-Rac assay for the detection of the level of ARF1 palmitoylation. Data are mean ± s.e.m.

Fig. S2. Lysosome-independent PPT2 may encounter ARF1 for depalmitoylation. A-B) Time-lapse confocal imaging of HEK293T cells expressing ARF1-GFP and PPT2-RFP/RFP-PPT2, LysoView^{TM}633 dye was added in culture medium to visualize live lysosome. The colocalization rate of ARF1-GFP and lysosome-independent PPT2 was measured.

Fig. S3. PPT2 regulated ARF1 depalmitoylation might be involved in modulating the Golgi/ER retrograde transport. A) ARF1-Flag or empty vector was expressed in WT or PPT2-KO cells, lysates of which were pulled down by Flag antibody conjugated beads to examine potential binding proteins. **B-C**) The levels of β-actin (***P*=0.0052, paired t-test, 2-tailed, n=3 repeats) and β-COP (****P*=0.0003, paired t-test, 2-tailed, n=3 repeats) associated with ARF1 were quantified. **D-E**) ARF1-Flag was expressed in WT or PPT2-KO HEK293T cells, and the post-nuclear homogenates were fractionated in 5-35% OptiPrep gradients and analyzed by Western blots to detect protein distributions (**D**), and protein distributions in relation to total population were profiled (**E**). Data are mean ± s.e.m.

Fig. S4. Deletion of ZDHHC8 in HEK293T cells. A) Targeting scheme of the truncation in the 2nd exon (enzymatic center) of human ZDHHC8 using CRISPR/Cas9 with two gRNA. Red lines indicate two cut sites in the introns of human ZDHHC8. **B**) The sequences of WT genomic DNA and mutation DNA. 125 bp were deleted from the mutation DNA. Additionally, representative sequencing result of ZDHHC8-KO HEK293T cells was provided. **C**) The genotyping products (WT, 275 bp, ZDHHC8-KO, 151 bp) were analyzed by electrophoresis in agarose gel.

Fig. S5. Deletion of PPT2 in HEK293T cells. A) Targeting scheme of the truncation in the 2nd exon (enzymatic center) of human PPT2 using CRISPR/Cas9 with two gRNA. Red lines indicate two cut sites in the introns of human PPT2. **B**) The sequences of WT genomic DNA and mutation DNA. 99 bp were deleted from the mutation DNA. Additionally, representative sequencing result of PPT2-KO HEK293T cells was provided. **C**) The genotyping products (WT, 244 bp, PPT2-KO, 146 bp) were analyzed by electrophoresis in agarose gel.

Fig. S6. Deletion of ARF1 in HEK293T cells. A) Targeting scheme of the truncation in the 2nd exon of human ARF1 using CRISPR/Cas9 with two gRNA. Red lines indicate two cut sites in the introns of

human ARF1. **B**) The sequences of WT genomic DNA and mutation DNA. 168 bp were deleted from the mutation DNA. Additionally, representative sequencing result of ARF1-KO HEK293T cells was provided. **C**) The deletion of ARF1 was verified at the protein level in ARF1-KO HEK293T cells.

Fig. S7. Generation of PPT2-KO mice. A) Targeting scheme of the truncation in the 2-6 exons of mouse PPT2. The deletion mutation was introduced into mouse fertilized eggs (C57/B6 background) using CRISPR/Cas9 with two gRNA. Red lines indicate two cut sites in the introns of mouse PPT2. **B**) Representative sequencing result of PPT2-KO mice and deletion of 3914 bp was created within the genomic DNA of PPT2. **C**) The evidence at the protein level was not shown because the commercially available PPT2-antibody lacks specificity. Rather, the results of the genotyping were presented to illustrate the varied genotypes of WT, heterozygotes, and homozygotes. **D**) Mice testis were collected from WT or PPT2-KO mice and used to prepare total protein lysates, which were subjected to ABE assay to evaluate the level of ARF1 expression and its palmitoylation.

Fig. S8. Uncropped blots of Fig. 1.

Fig. S9. Uncropped blots of Fig. 2.

Fig. S10. Uncropped blots of Fig. 3.

Fig. S11. Uncropped blots of Fig. 4.

Fig. S12. Uncropped blots of Fig. 5.

Fig. S1

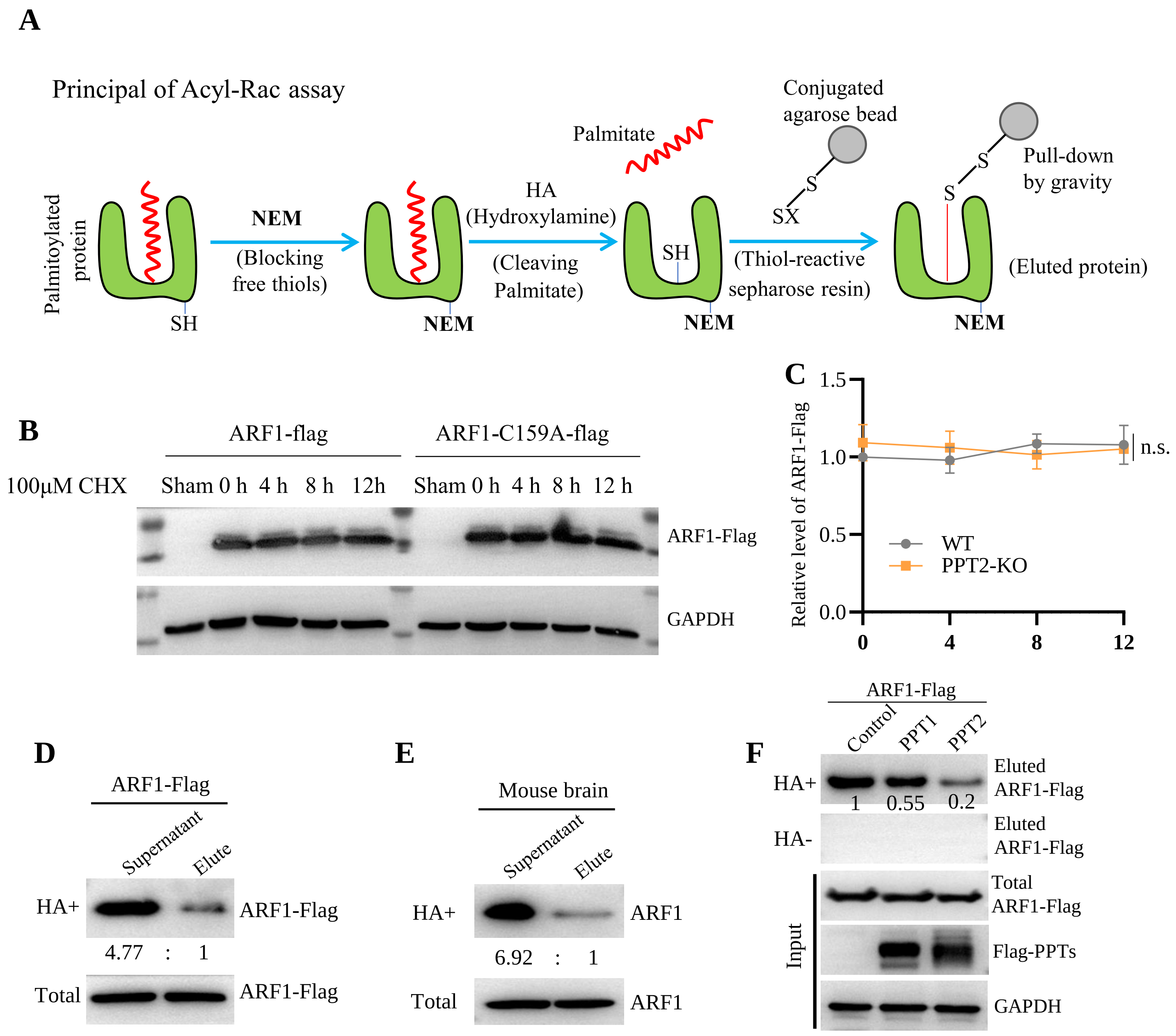
A
Principal of Acyl-Rac assay
Palmitoylated protein
SH
NEM
(Blocking free thiols)
NEM
HA (Hydroxylamine)
(Cleaving Palmitate)
Palmitate
SH
NEM
Conjugated agarose bead
S
SX
(Thiol-reactive sepharose resin)
S
S
Pull-down by gravity
(Eluted protein)
NEM
B
ARF1-flag
ARF1-C159A-flag
100μM CHX
Sham 0 h 4 h 8 h 12h
Sham 0 h 4 h 8 h 12 h
ARF1-Flag
GAPDH
C
Relative level of ARF1-Flag
1.5
1.0
0.5
0.0
0
4
8
12
n.s.
WT
PPT2-KO
D
ARF1-Flag
Supernatant
Elute
HA+
ARF1-Flag
4.77 : 1
Total
ARF1-Flag
E
Mouse brain
Supernatant
Elute
HA+
ARF1
6.92 : 1
Total
ARF1
F
ARF1-Flag
Control
PPT1
PPT2
HA+
Eluted ARF1-Flag
1
0.55
0.2
HA-
Eluted ARF1-Flag
Input
Total ARF1-Flag
Flag-PPTs
GAPDH

Fig. S2

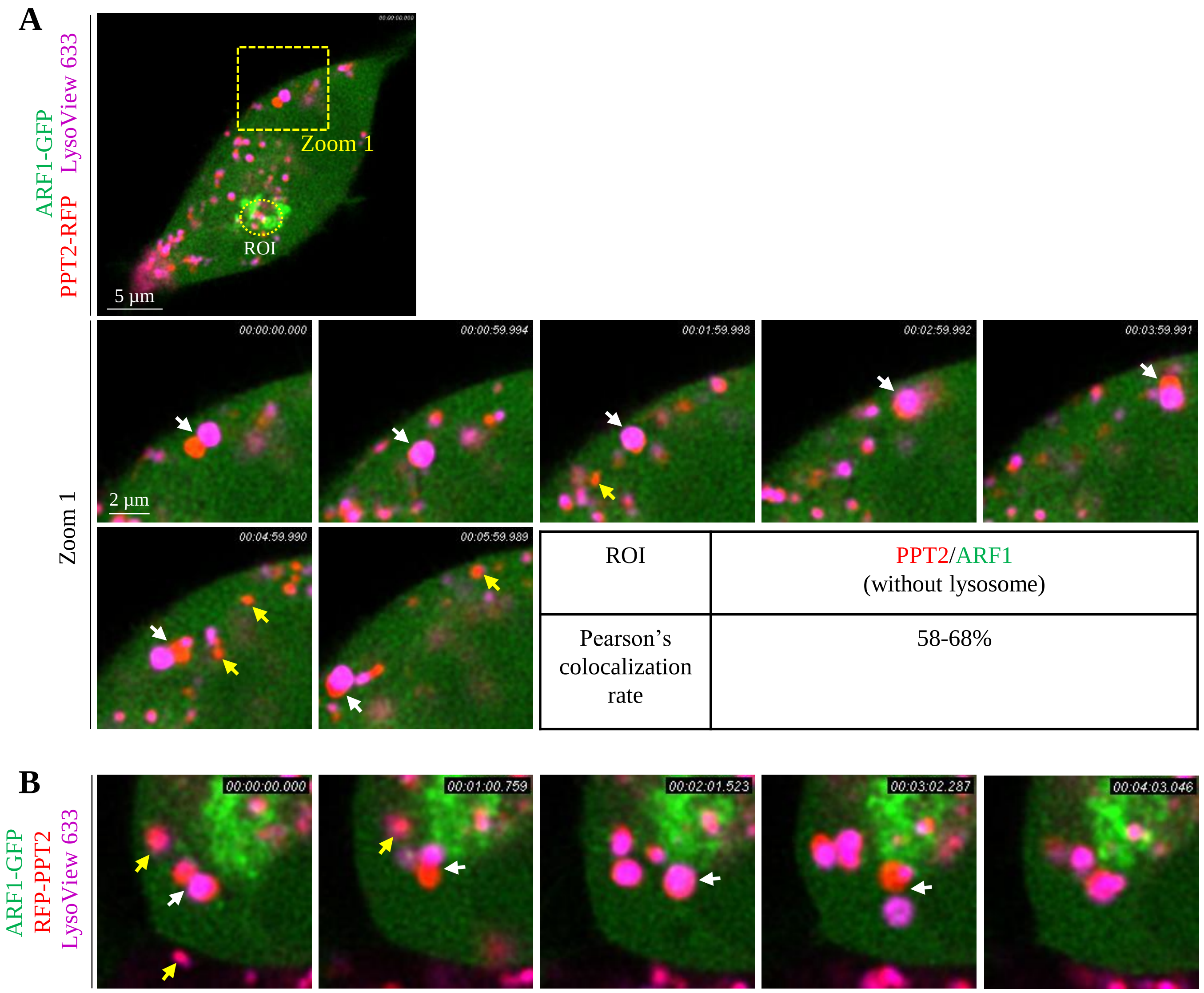

ROI	PPT2/ARF1 (without lysosome)
Pearson's colocalization rate	58-68%

Fig. S3

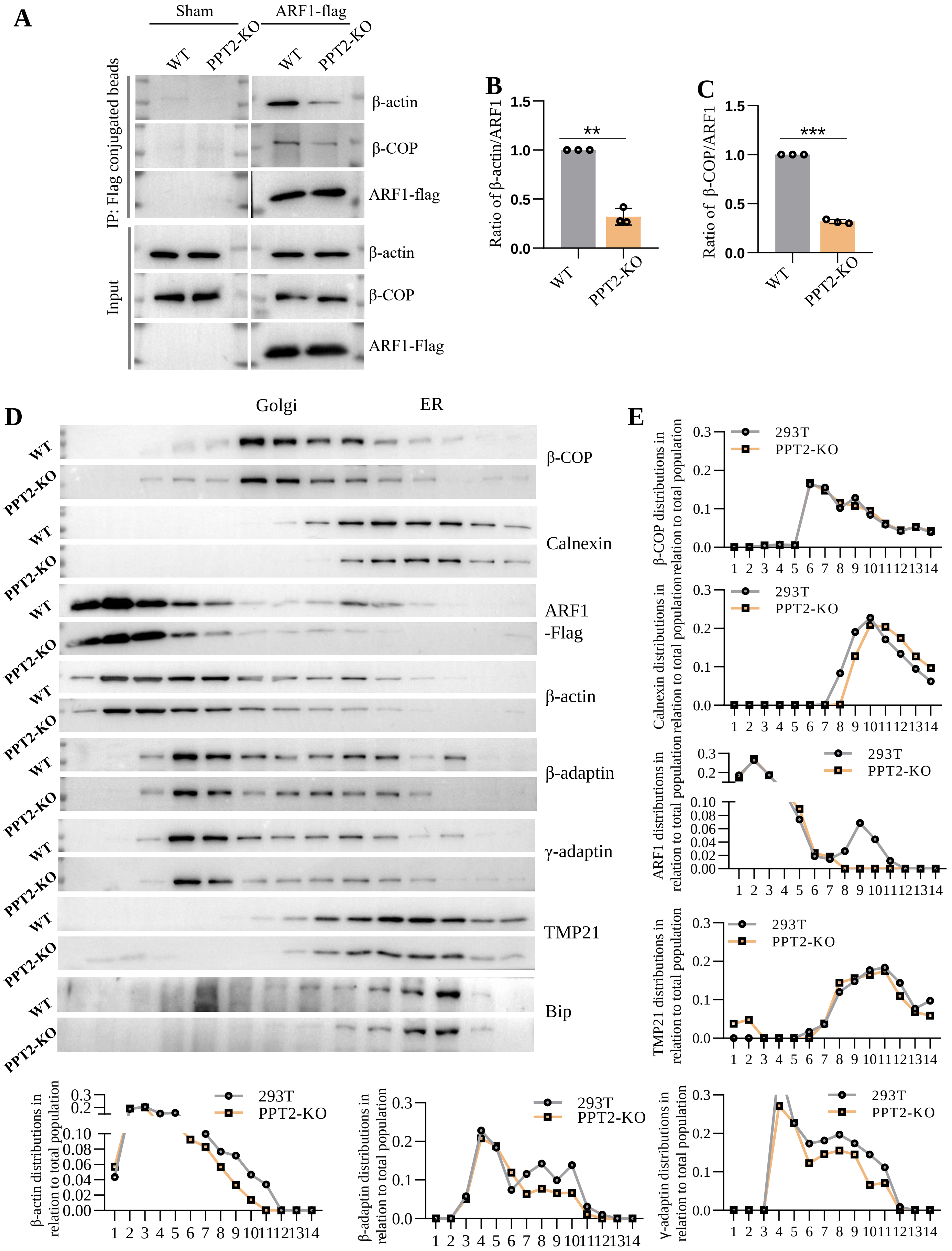
A
Sham
ARF1-flag
WT
PPT2-KO
IP: Flag conjugated beads
Input
β-actin
β-COP
ARF1-flag
ARF1-Flag
B
Ratio of β-actin/ARF1
**
C
Ratio of β-COP/ARF1
***
D
Golgi
ER
β-COP
Calnexin
ARF1
-Flag
β-actin
β-adaptin
γ-adaptin
TMP21
Bip
E
293T
PPT2-KO
β-COP distributions in relation to total population
Calnexin distributions in relation to total population
ARF1 distributions in relation to total population
TMP21 distributions in relation to total population
β-actin distributions in relation to total population
β-adaptin distributions in relation to total population
γ-adaptin distributions in relation to total population

Fig. S4

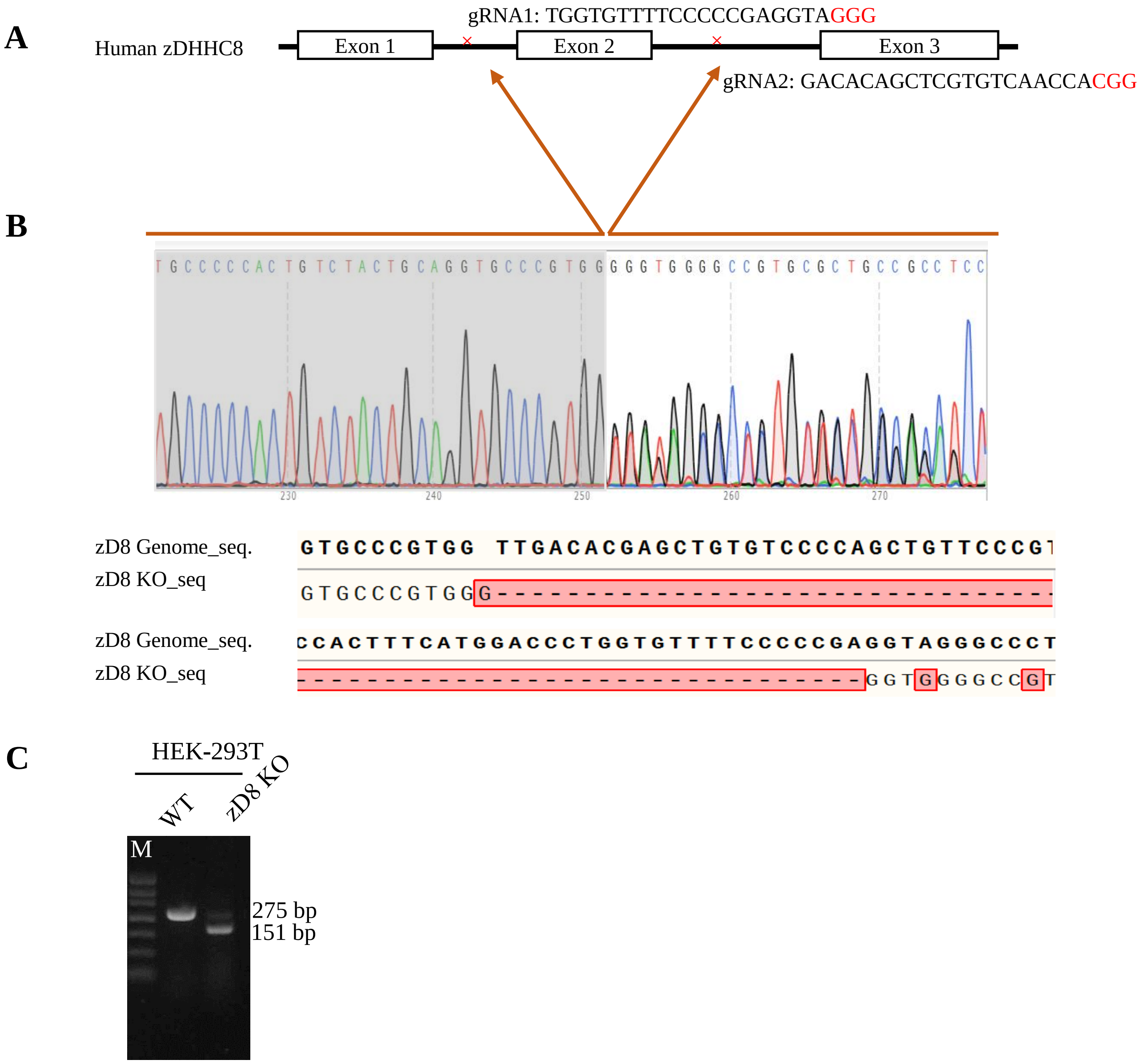
A
Human zDHHC8
Exon 1
Exon 2
Exon 3
gRNA1: TGGTGTTTTCCCCCGAGGTAGGG
gRNA2: GACACAGCTCGTGTCAACCACGG
B
230
240
250
260
270
zD8 Genome_seq.
GTGCCCGTGG TTGACACGAGCTGTGTCCCCAGCTGTTCCCGT
zD8 KO_seq
GTGCCCGTGGG
zD8 Genome_seq.
CCACTTTCATGGACCCTGGTGTTTTCCCCCGAGGTAGGGCCCT
zD8 KO_seq
GGTGGGGCCGT
C
HEK-293T
WT
zD8 KO
M
275 bp
151 bp

Fig. S5

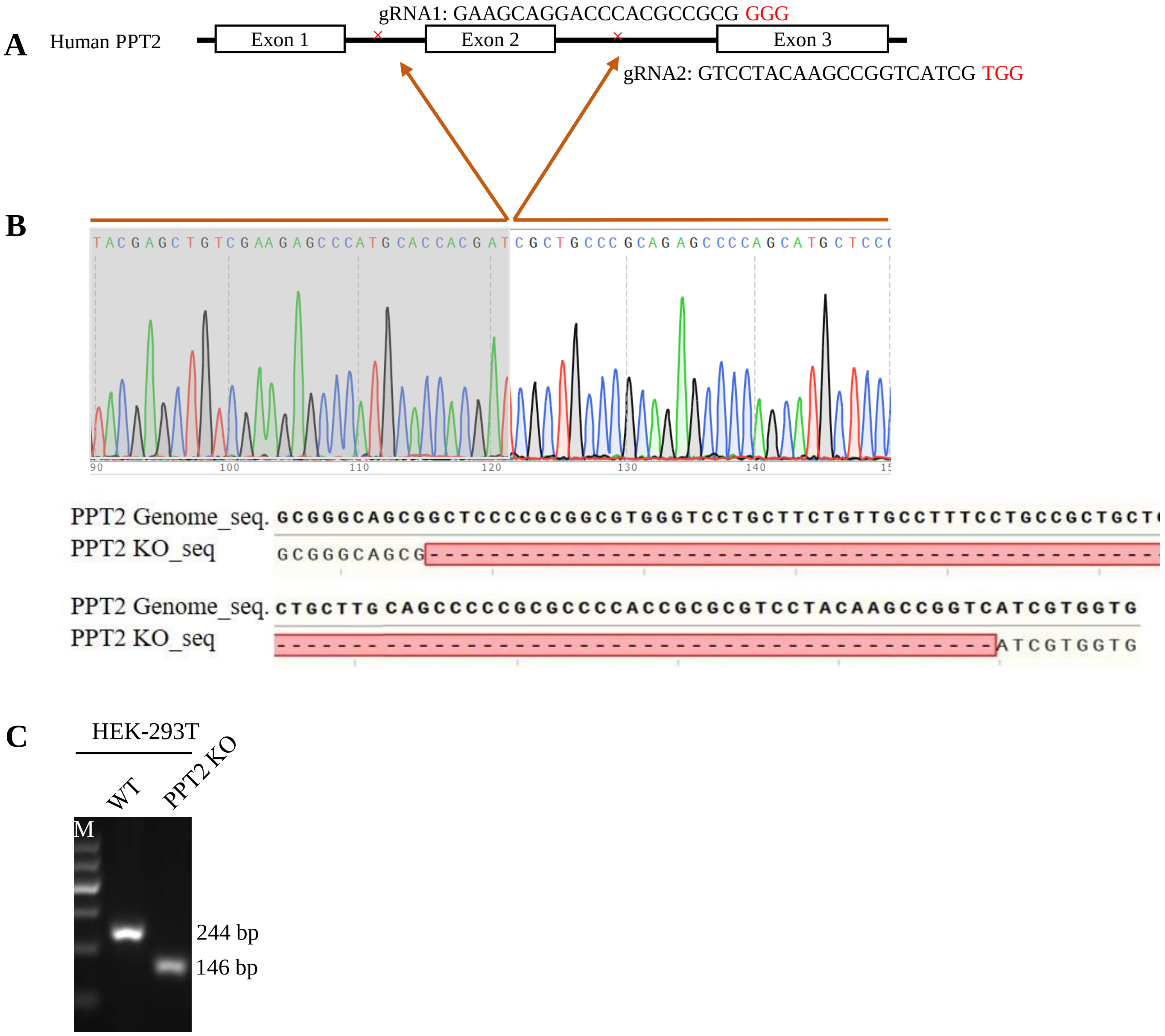
A
Human PPT2
Exon 1
Exon 2
Exon 3
gRNA1: GAAGCAGGACCCACGCCGCG GGG
gRNA2: GTCCTACAAGCCGGTCATCG TGG
B
PPT2 Genome_seq. GCGGGCAGCGGCTCCCCGCGGCGTGGGTCCTGCTTCTGTTGCCTTTCCTGCCGCTGCT
PPT2 KO_seq GCGGGCAGCG
PPT2 Genome_seq. CTGCTTG CAGCCCCCGCGCCCCACCGCGCGTCCTACAAGCCGGTCATCGTGGTG
PPT2 KO_seq ATCGTGGTG
C
HEK-293T
WT
PPT2 KO
M
244 bp
146 bp

Fig. S6

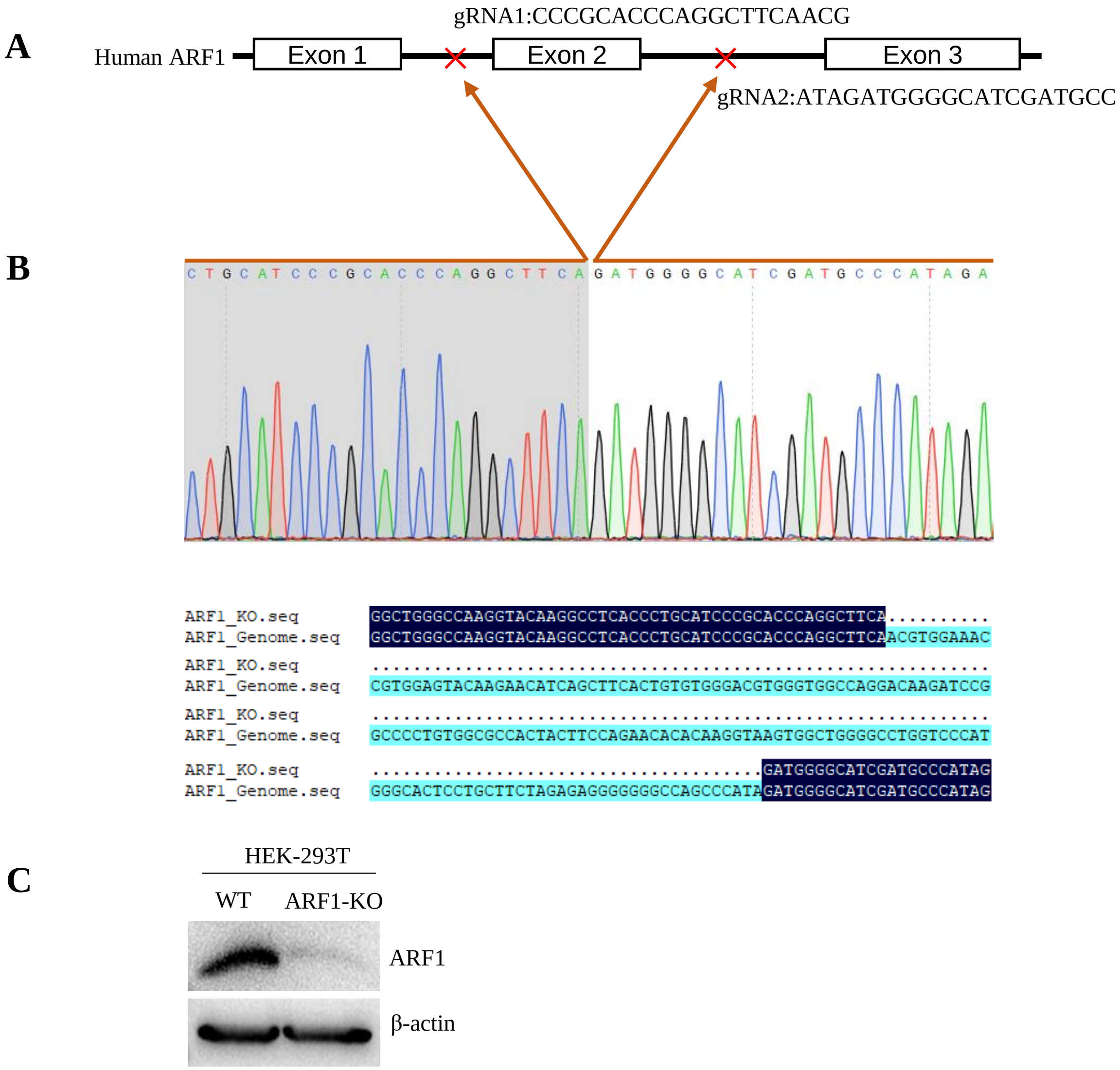
A
gRNA1:CCCGCACCCAGGCTTCAACG
Human ARF1
Exon 1
Exon 2
Exon 3
gRNA2:ATAGATGGGGCATCGATGCC
B
CTGCATCCCGCACCCAGGCTTCAGATGGGGCATCGATGCCCATAGA
ARF1_KO.seq GGCTGGGCCAAGGTACAAGGCCTCACCCTGCATCCCGCACCCAGGCTTCA
ARF1_Genome.seq GGCTGGGCCAAGGTACAAGGCCTCACCCTGCATCCCGCACCCAGGCTTCAACGTGGAAAC
ARF1_KO.seq
ARF1_Genome.seq CGTGGAGTACAAGAACATCAGCTTCACTGTGTGGGACGTGGGTGGCCAGGACAAGATCCG
ARF1_KO.seq
ARF1_Genome.seq GCCCCTGTGGCGCCACTACTTCCAGAACACACAAGGTAAGTGGCTGGGGCCTGGTCCCAT
ARF1_KO.seq GATGGGGCATCGATGCCCATAG
ARF1_Genome.seq GGGCACTCCTGCTTCTAGAGAGGGGGGGCCAGCCCATAGATGGGGCATCGATGCCCATAG
C
HEK-293T
WT
ARF1-KO
ARF1
β-actin

Fig. S7

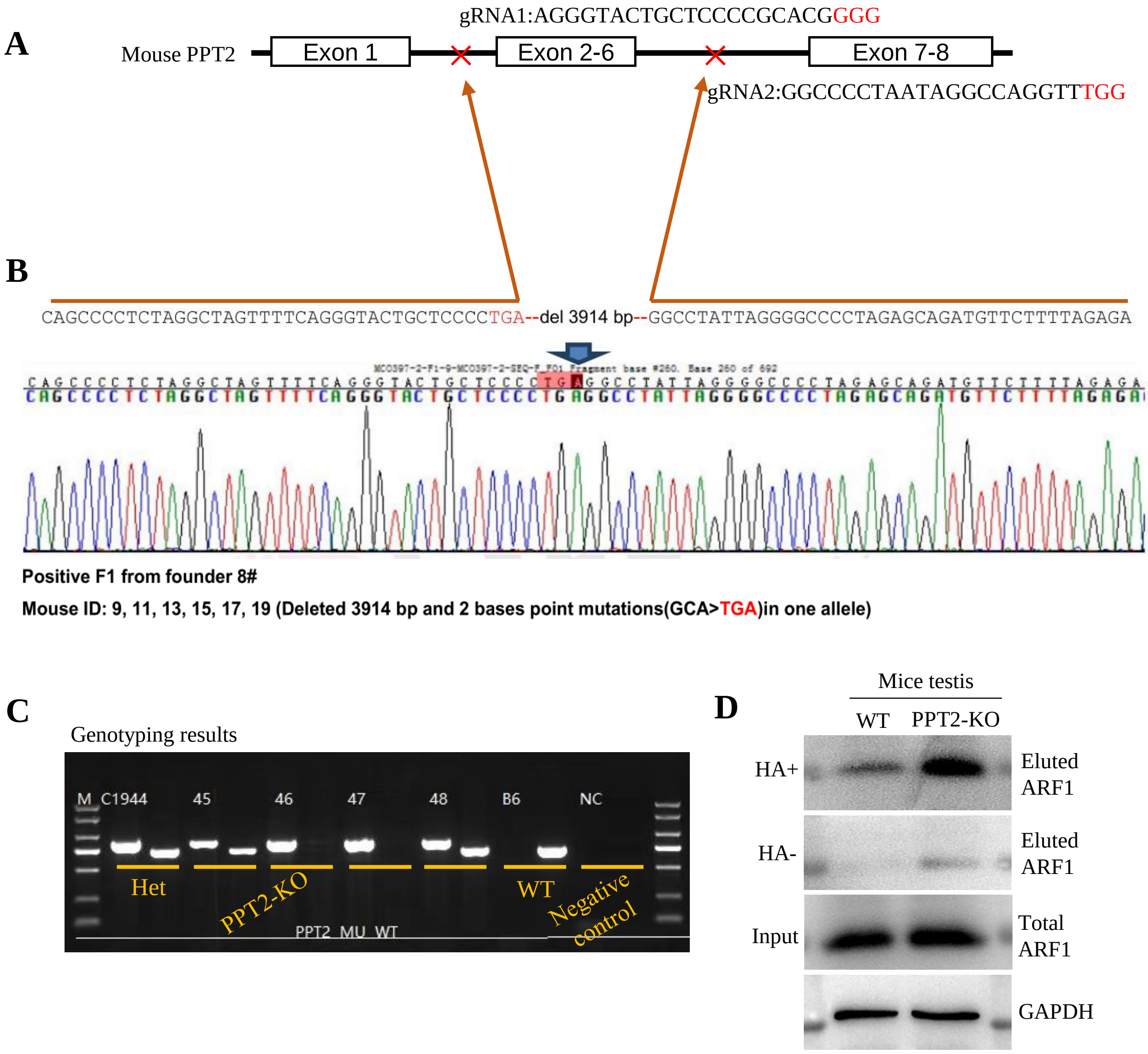
A
gRNA1:AGGGTACTGCTCCCCGCACGGGG
Mouse PPT2
Exon 1
Exon 2-6
Exon 7-8
gRNA2:GGCCCCTAATAGGCCAGGTTTGG
B
CAGCCCCTCTAGGCTAGTTTTCAGGGTACTGCTCCCCTGA--del 3914 bp--GGCCTATTAGGGGCCCCTAGAGCAGATGTTCTTTTAGAGA
Positive F1 from founder 8#
Mouse ID: 9, 11, 13, 15, 17, 19 (Deleted 3914 bp and 2 bases point mutations(GCA>TGA)in one allele)
C
Genotyping results
M C1944 45 46 47 48 B6 NC
Het
PPT2-KO
WT
Negative control
PPT2 MU WT
D
Mice testis
WT PPT2-KO
HA+
Eluted ARF1
HA-
Eluted ARF1
Input
Total ARF1
GAPDH

Fig. S8

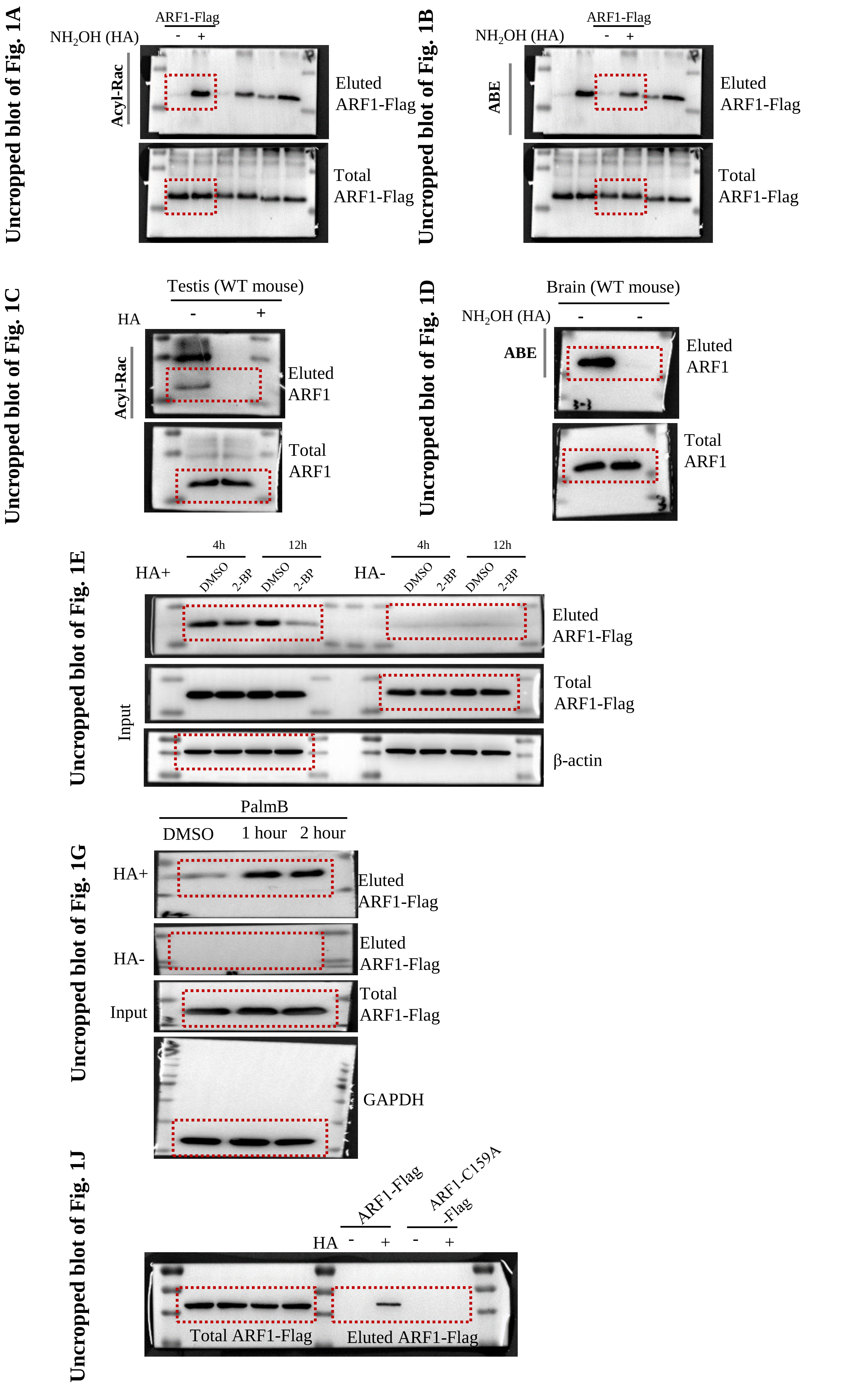

Uncropped blot of Fig. 1A
ARF1-Flag
NH2OH (HA)
- +
Acyl-Rac
Eluted ARF1-Flag
Total ARF1-Flag
Uncropped blot of Fig. 1B
ARF1-Flag
NH2OH (HA)
- +
ABE
Eluted ARF1-Flag
Total ARF1-Flag
Uncropped blot of Fig. 1C
Testis (WT mouse)
HA
- +
Acyl-Rac
Eluted ARF1
Total ARF1
Uncropped blot of Fig. 1D
Brain (WT mouse)
NH2OH (HA)
- -
ABE
Eluted ARF1
Total ARF1
Uncropped blot of Fig. 1E
4h
12h
HA+
DMSO
2-BP
HA-
Eluted ARF1-Flag
Input
Total ARF1-Flag
β-actin
Uncropped blot of Fig. 1G
PalmB
DMSO
1 hour
2 hour
HA+
Eluted ARF1-Flag
HA-
Eluted ARF1-Flag
Input
Total ARF1-Flag
GAPDH
Uncropped blot of Fig. 1J
ARF1-Flag
ARF1-C159A -Flag
HA
- + - +
Total ARF1-Flag
Eluted ARF1-Flag

Fig. S9

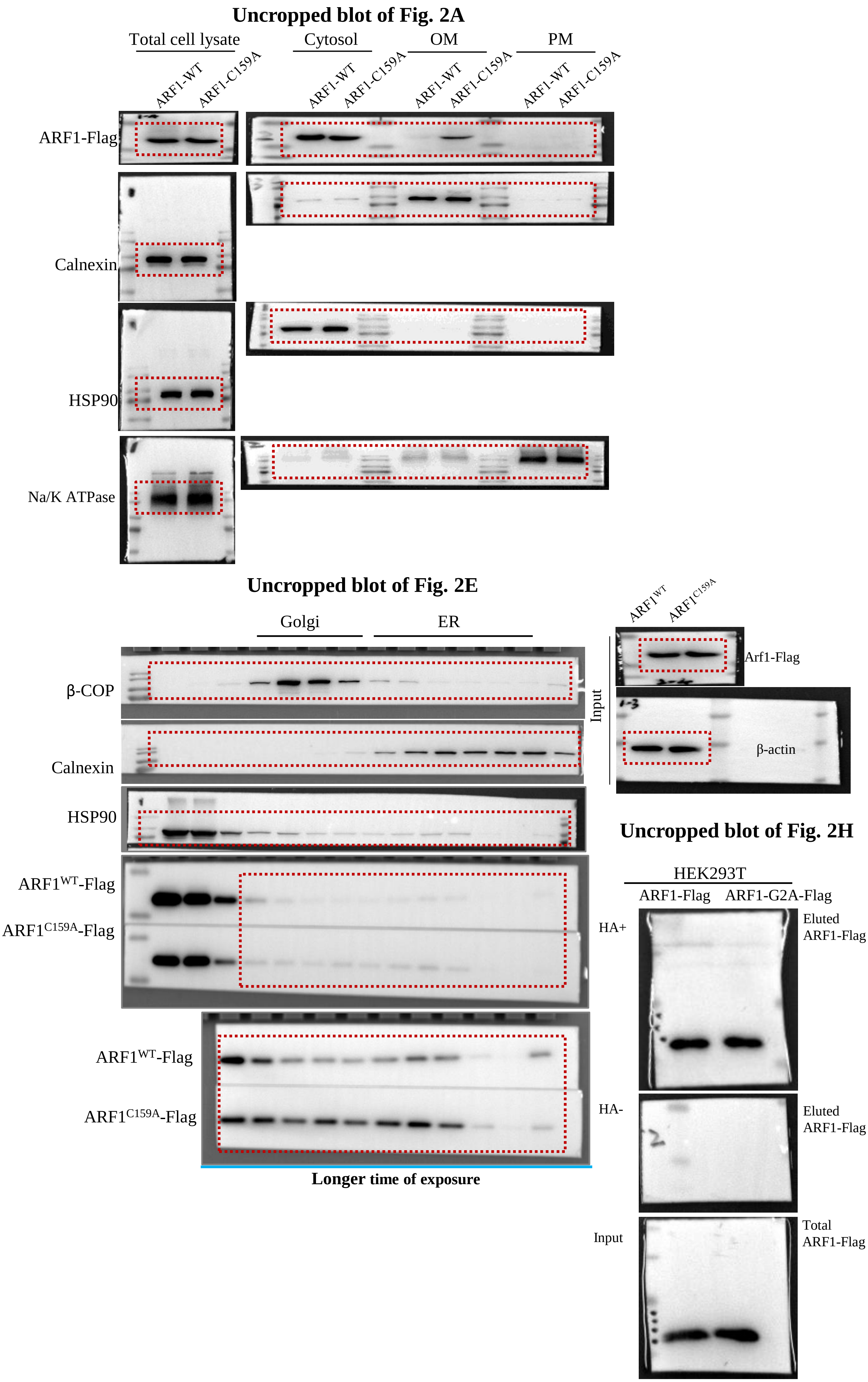

Uncropped blot of Fig. 2A
Total cell lysate
Cytosol
OM
PM
ARF1-WT
ARF1-C159A
ARF1-Flag
Calnexin
HSP90
Na/K ATPase
Uncropped blot of Fig. 2E
Golgi
ER
β-COP
Calnexin
HSP90
ARF1WT-Flag
ARF1C159A-Flag
Longer time of exposure
ARF1WT
ARF1C159A
Input
Arf1-Flag
β-actin
Uncropped blot of Fig. 2H
HEK293T
ARF1-Flag
ARF1-G2A-Flag
HA+
HA-
Input
Eluted ARF1-Flag
Total ARF1-Flag

Fig. S10

Uncropped blot of Fig. 3A

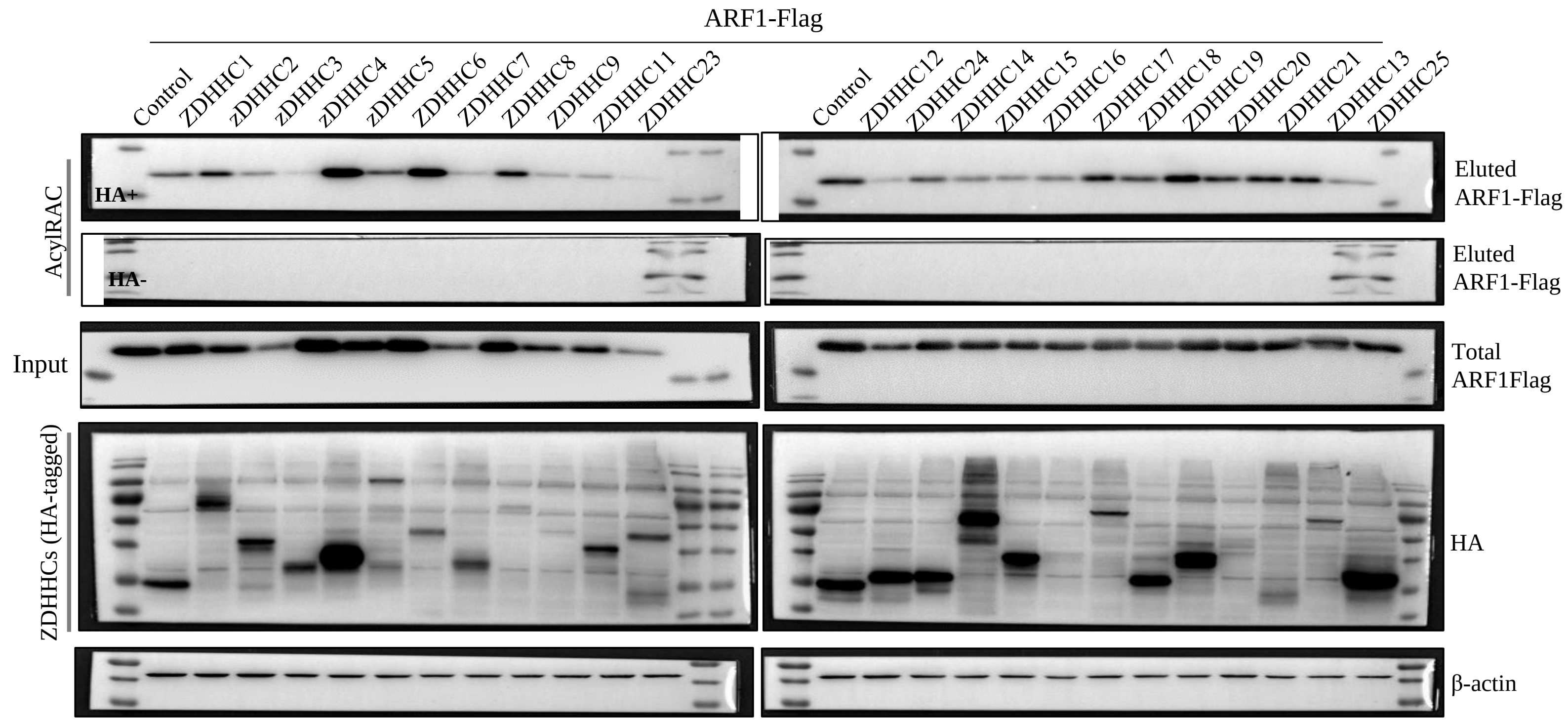

Uncropped blot of Fig. 3E

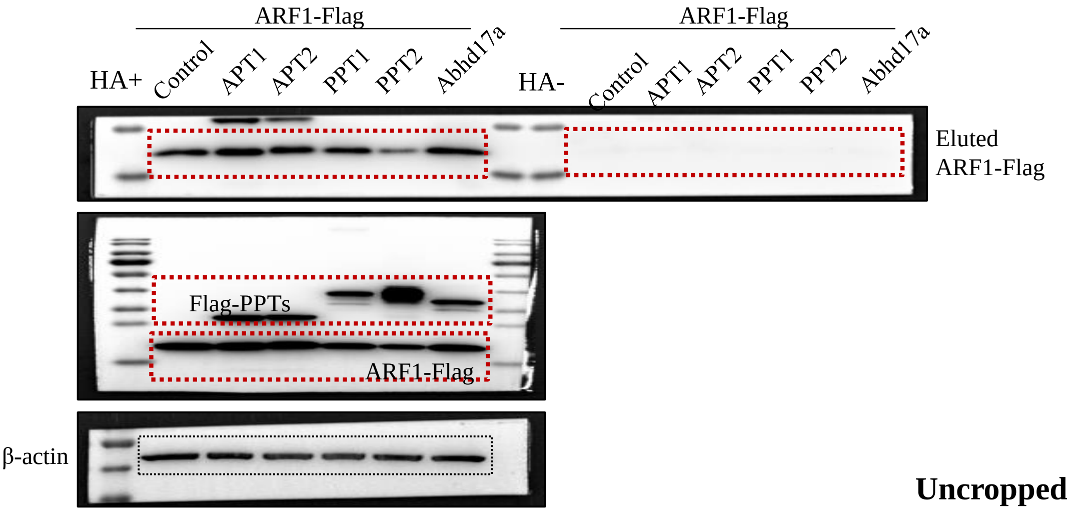

Uncropped blot of Fig. 3C

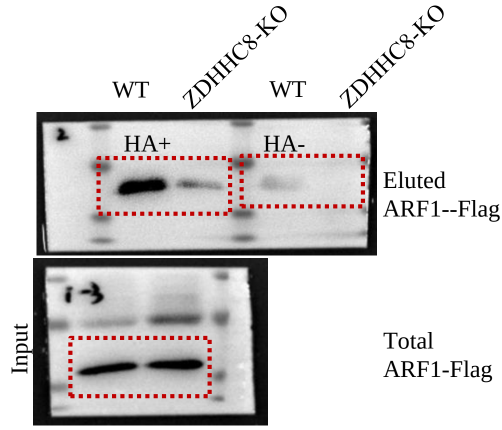

Uncropped blot of Fig. 3G

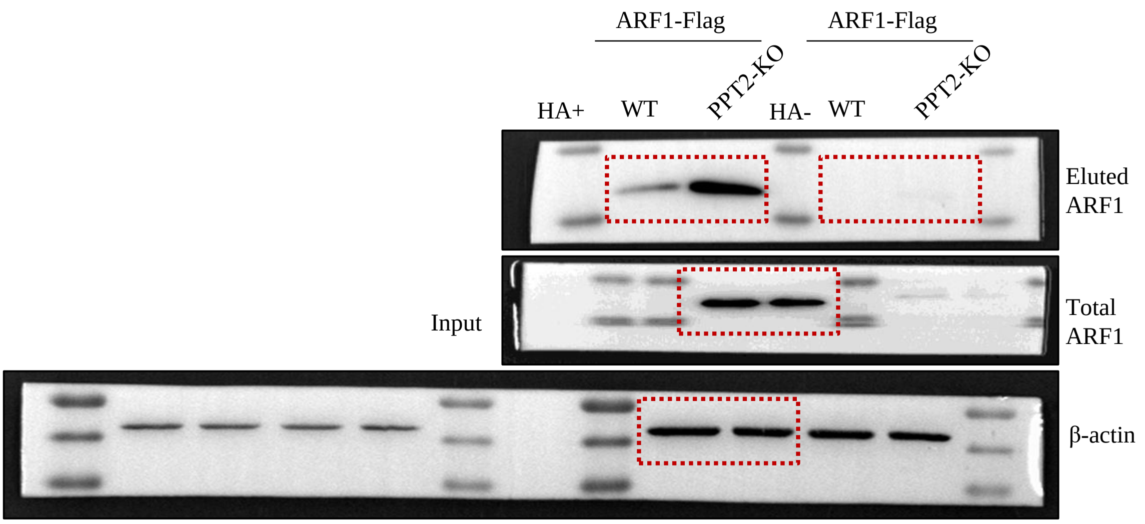

Fig. S11

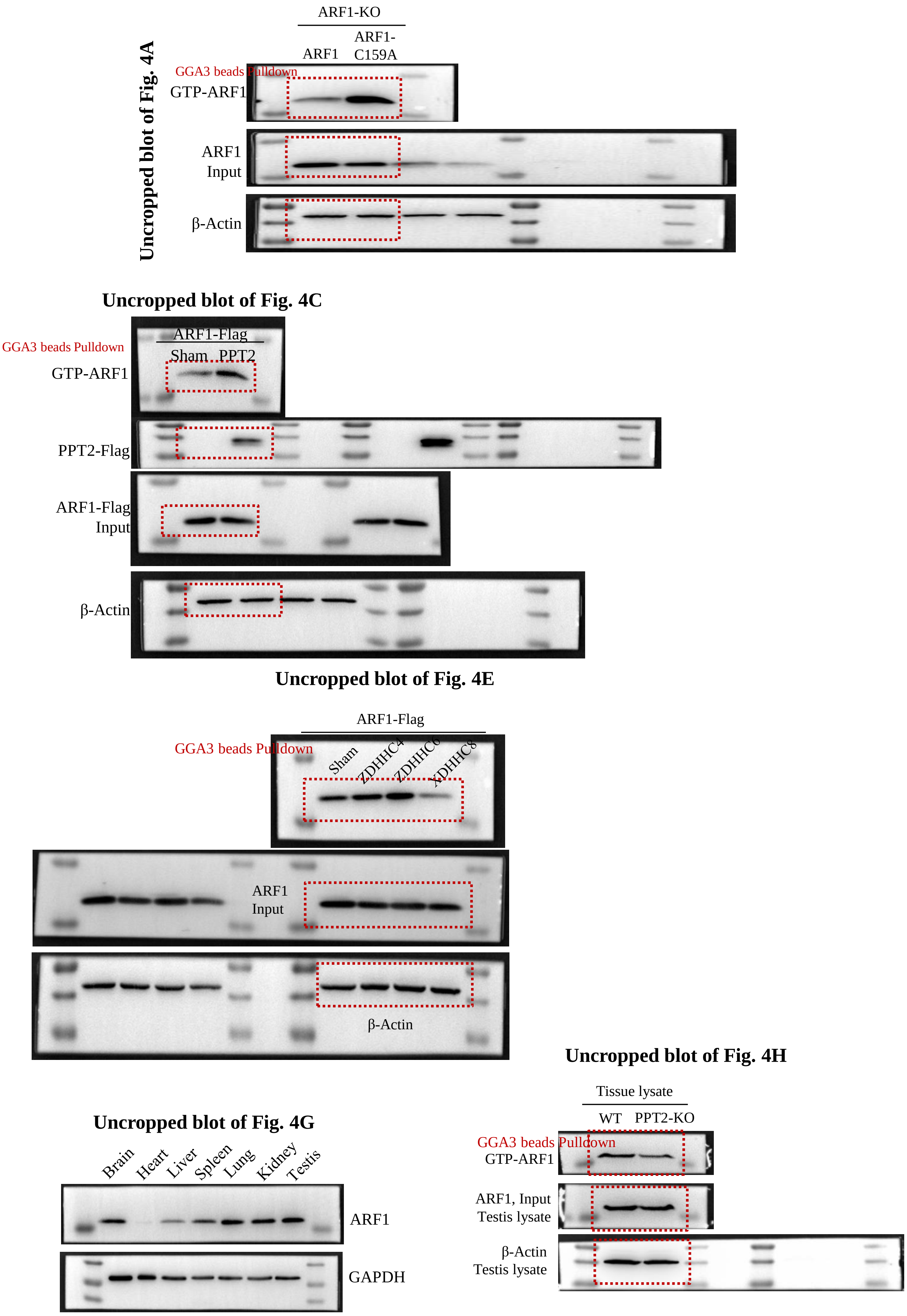

Uncropped blot of Fig. 4A
ARF1-KO
ARF1
ARF1-C159A
GGA3 beads Pulldown
GTP-ARF1
ARF1
Input
β-Actin
Uncropped blot of Fig. 4C
GGA3 beads Pulldown
ARF1-Flag
Sham
PPT2
GTP-ARF1
PPT2-Flag
ARF1-Flag
Input
β-Actin
Uncropped blot of Fig. 4E
ARF1-Flag
GGA3 beads Pulldown
Sham
ZDHHC4
ZDHHC6
ZDHHC8
ARF1
Input
β-Actin
Uncropped blot of Fig. 4H
Tissue lysate
WT
PPT2-KO
GGA3 beads Pulldown
GTP-ARF1
ARF1, Input
Testis lysate
β-Actin
Testis lysate
Uncropped blot of Fig. 4G
Brain
Heart
Liver
Spleen
Lung
Kidney
Testis
ARF1
GAPDH

Fig. S12

Uncropped blot of Fig. 5A

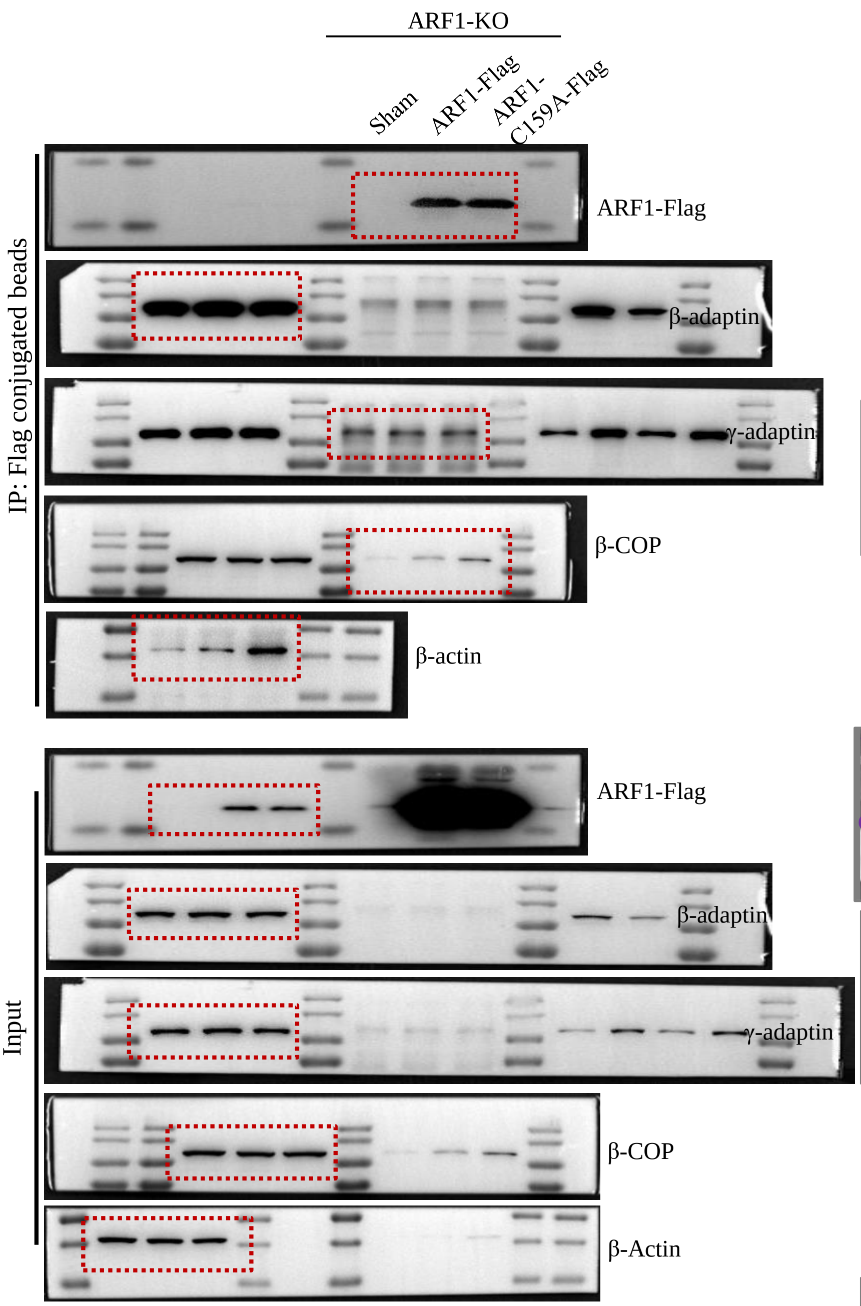

Uncropped blot of Fig. 5D

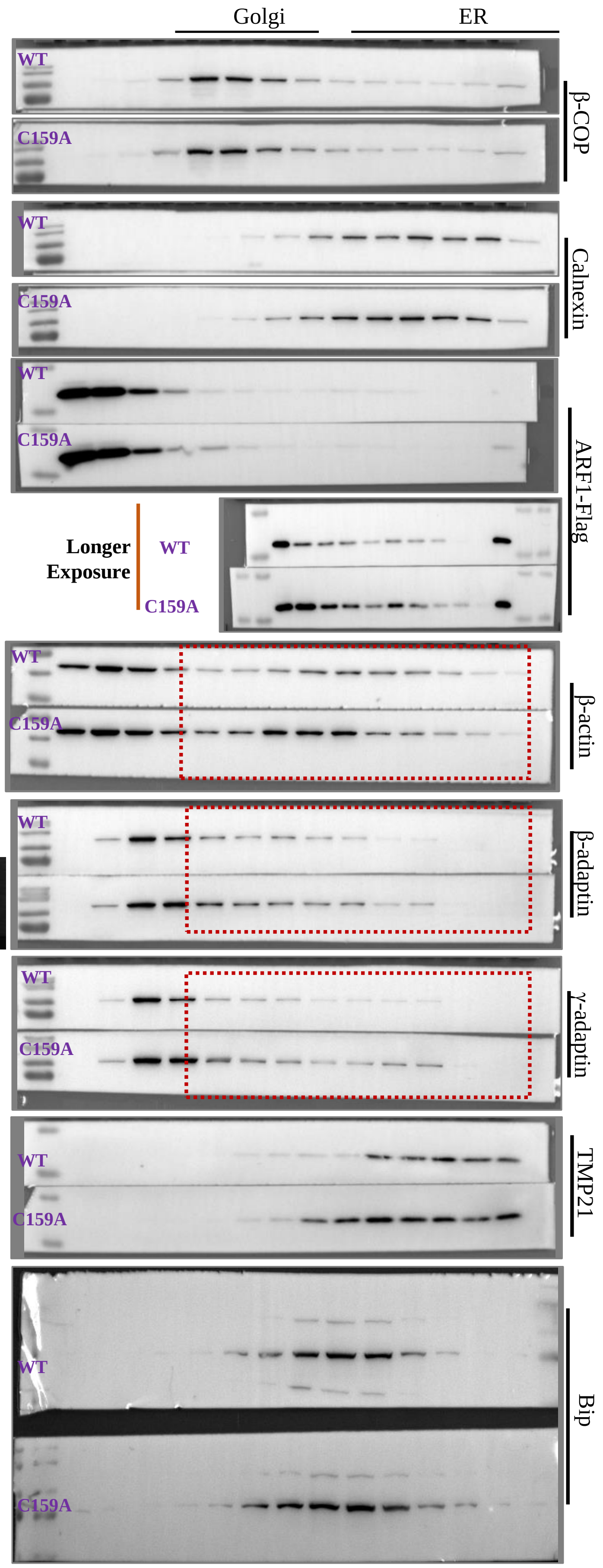

Supplement: Binder 2_Supplemental data [file mmc4.pdf]
