# Supplementary material for: The Activation of ARF1 Is Dynamically Regulated by its Palmitoylation
Source: Mol Cell Proteomics. 2026 May 14;25(6):101586. doi: 10.1016/j.mcpro.2026.101586 (PMC13273671; doi:10.1016/j.mcpro.2026.101586)

A0A0S2Z4Z8_SLPAPQDNDFLSR_2

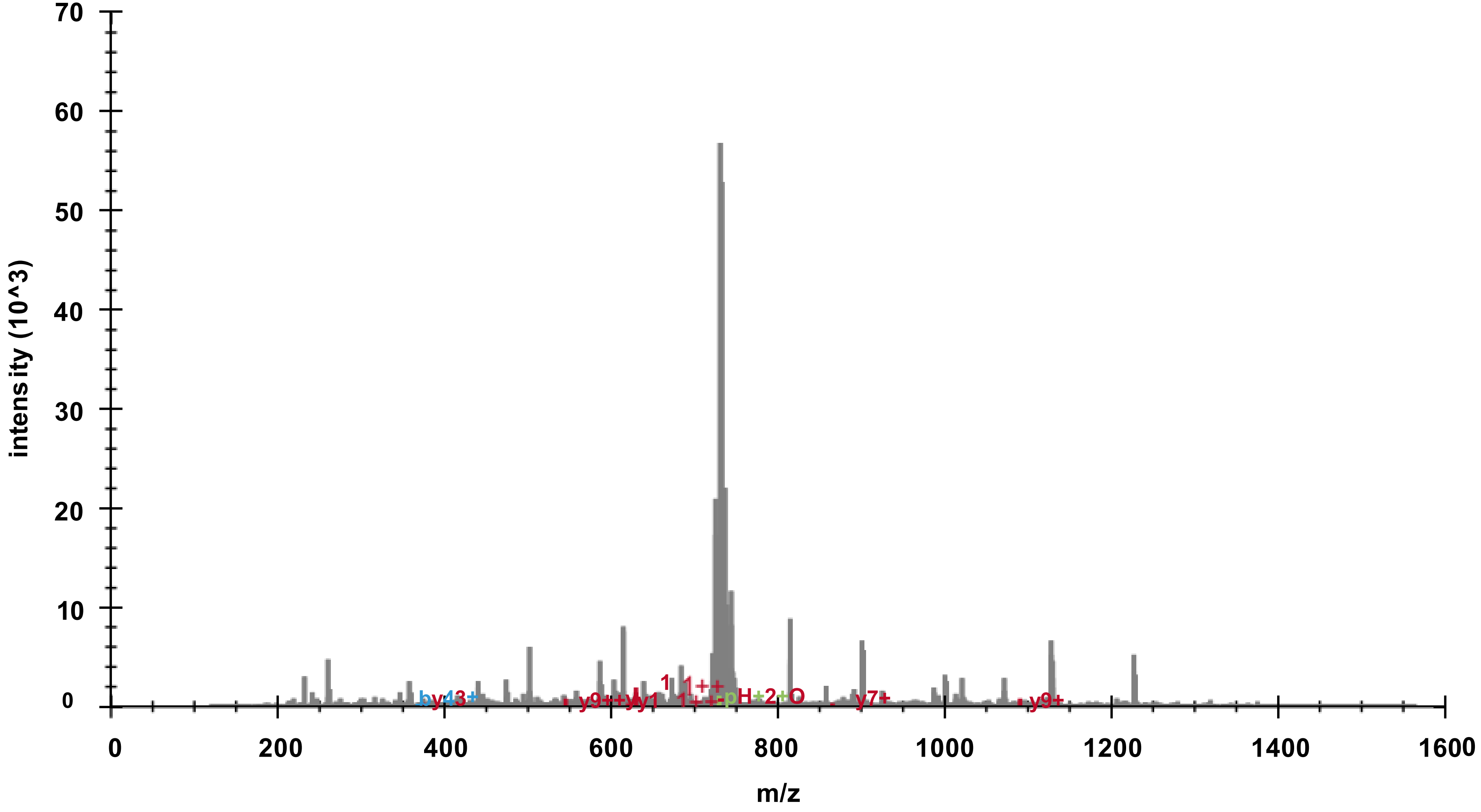

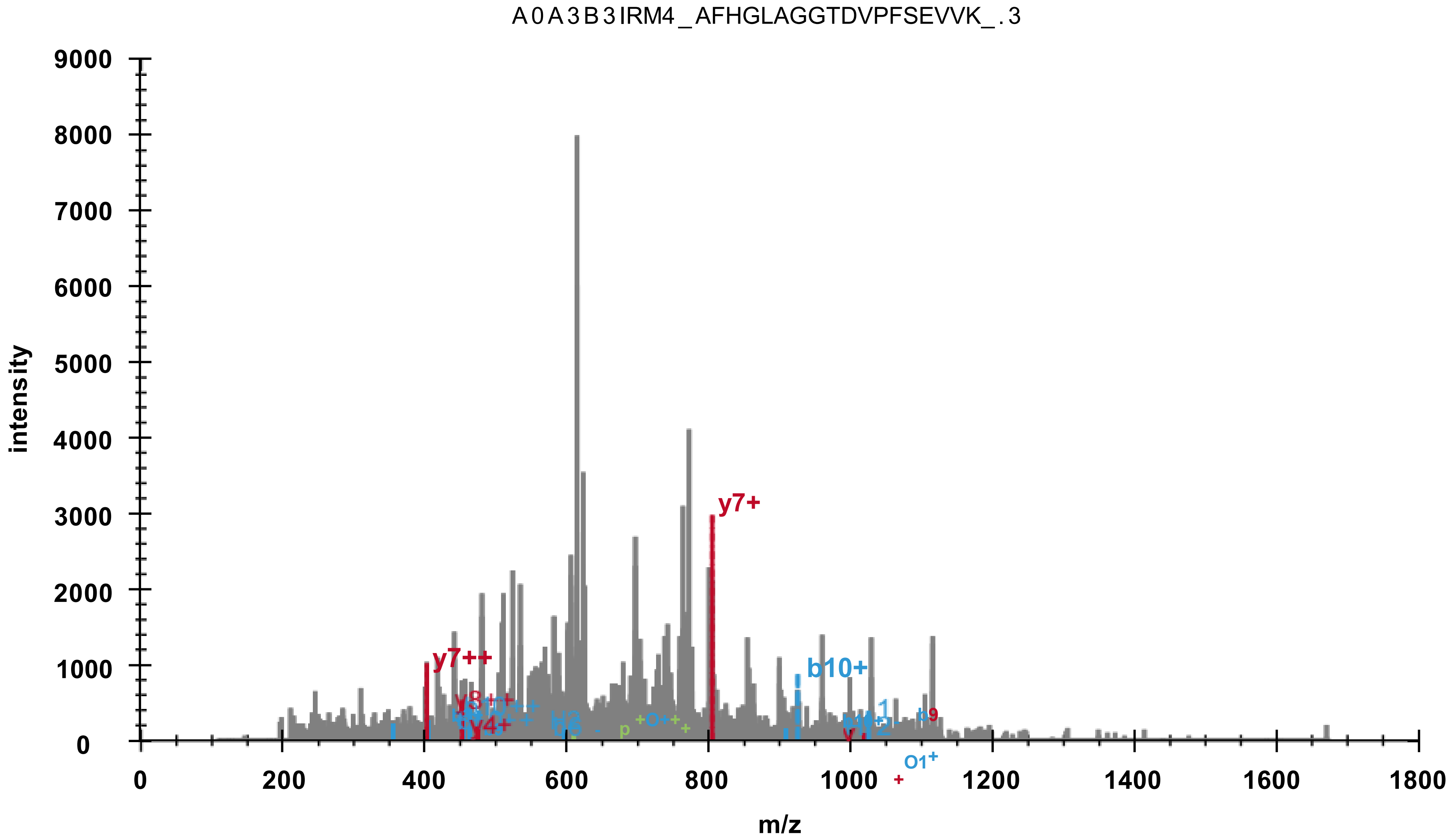
A0A3B3IRM4_AFHGLAGGTDVPFSEVVK_.3
intensity
m/z
y7+
y7++
b10+
y4+
0
1000
2000
3000
4000
5000
6000
7000
8000
9000
200
400
600
800
1000
1200
1400
1600
1800

A0A8V8TQP8_QDLPNAMNAAEITDKL__2

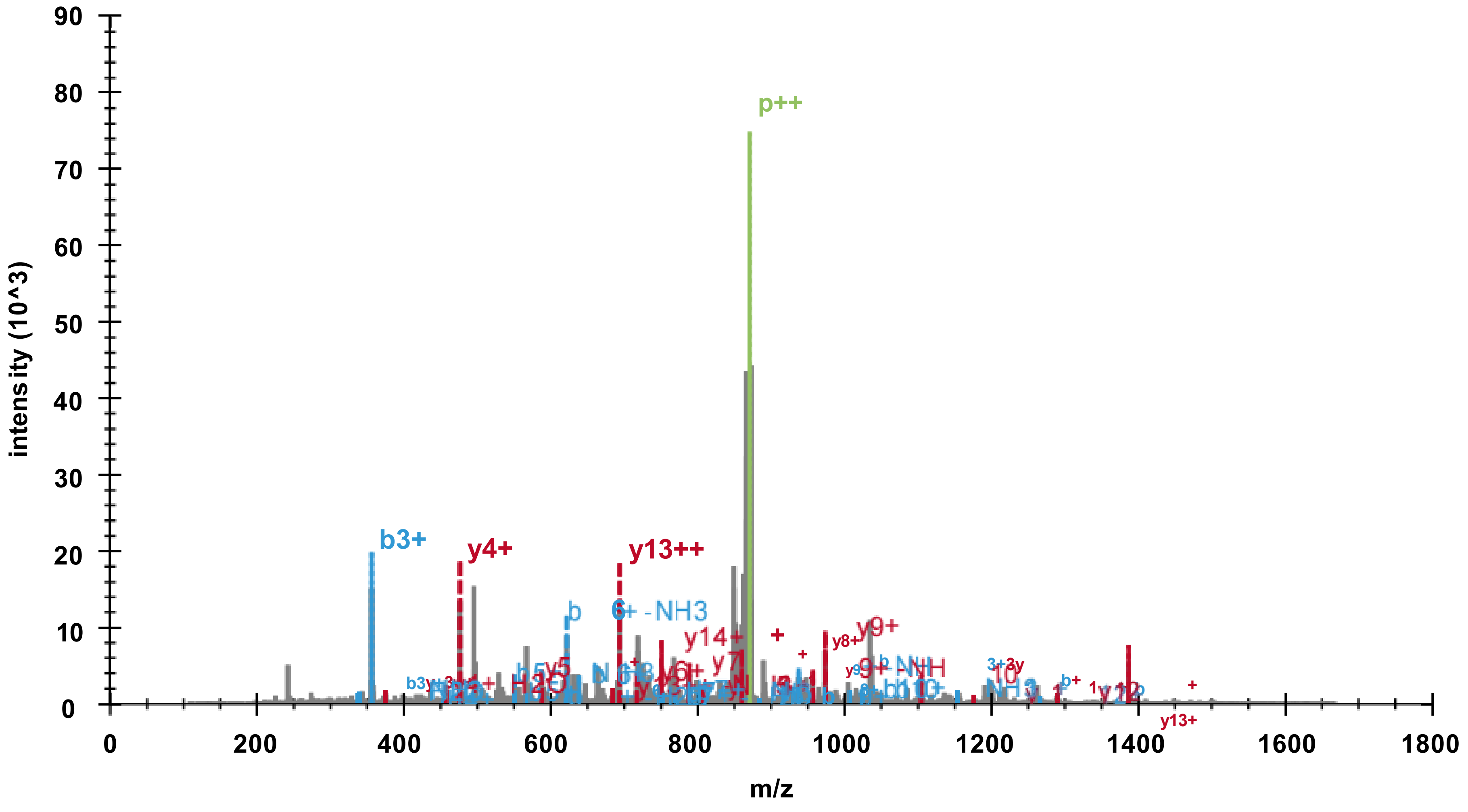

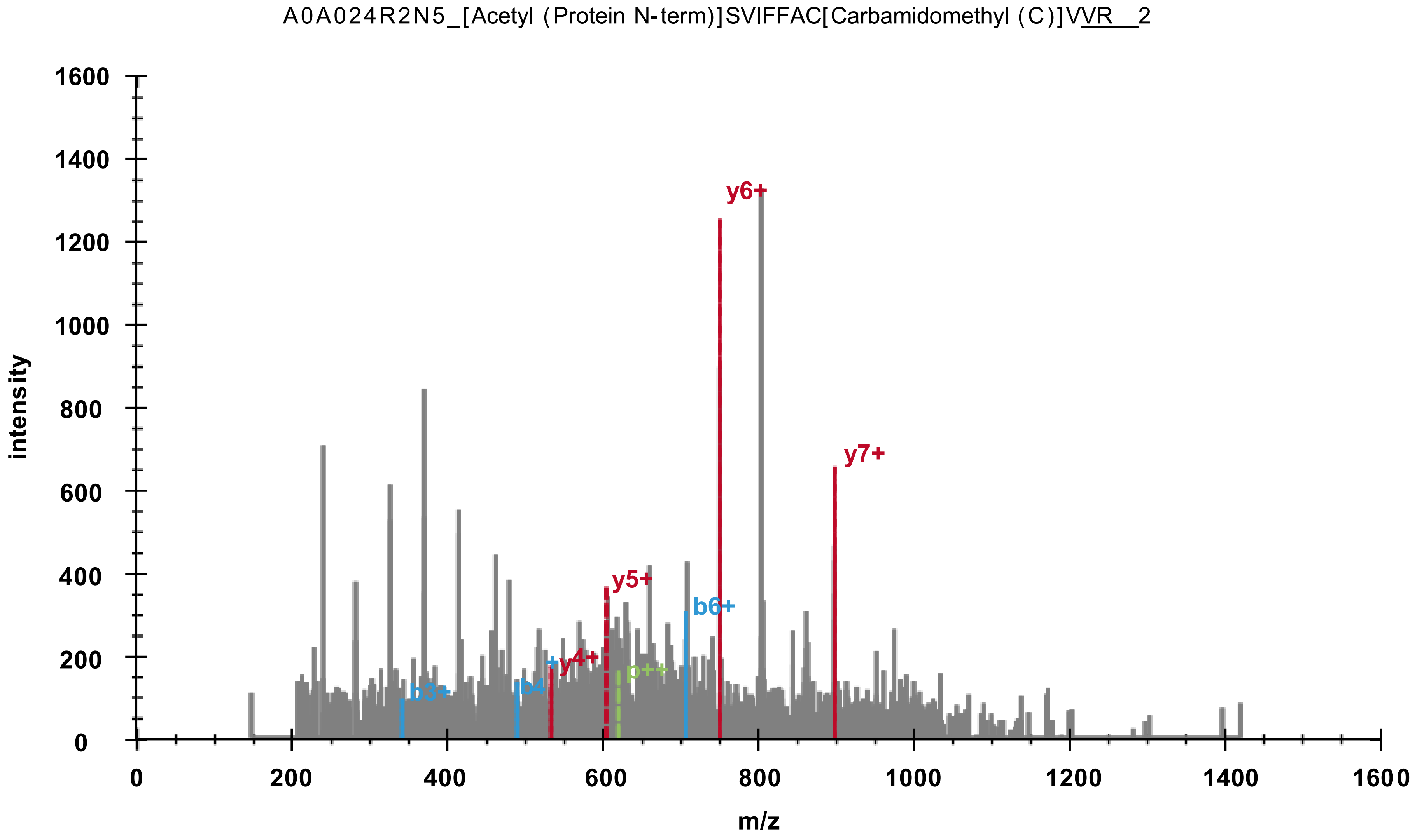
A0A024R2N5_[Acetyl (Protein N-term)]SVIFFAC[Carbamidomethyl (C)]VVR__2
intensity
m/z
y6+
y7+
y5+
b6+
y4+
p++
b3+
b4
0
200
400
600
800
1000
1200
1400
1600

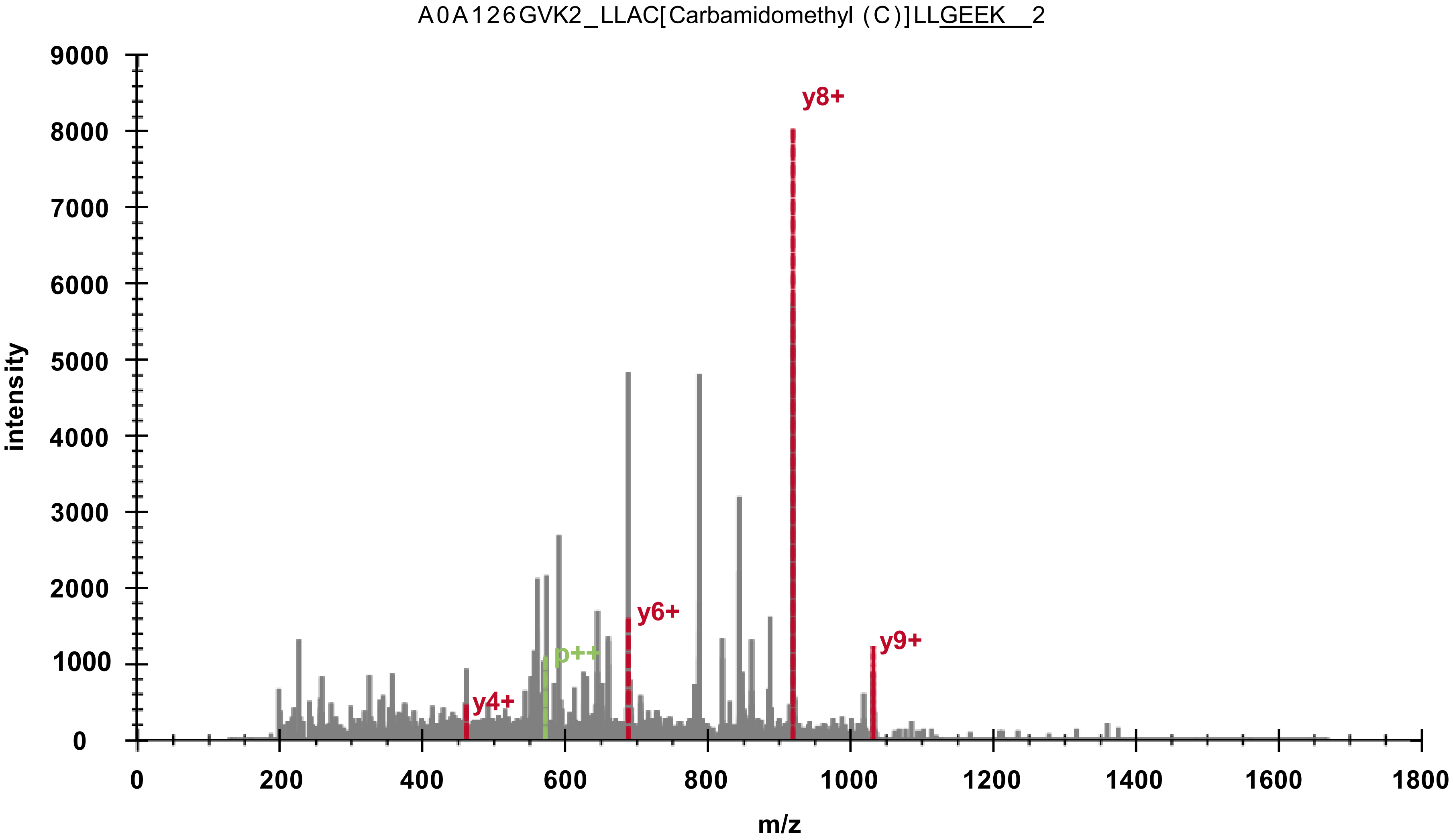
A0A126GVK2_LLAC[Carbamidomethyl (C)]LLGEEK__2
9000
8000
7000
6000
5000
4000
3000
2000
1000
0
intensity
y8+
y6+
p++
y9+
y4+
0
200
400
600
800
1000
1200
1400
1600
1800
m/z

A0A172WBW8_WSTAVEFC[Carbamidomethyl (C)]K__2

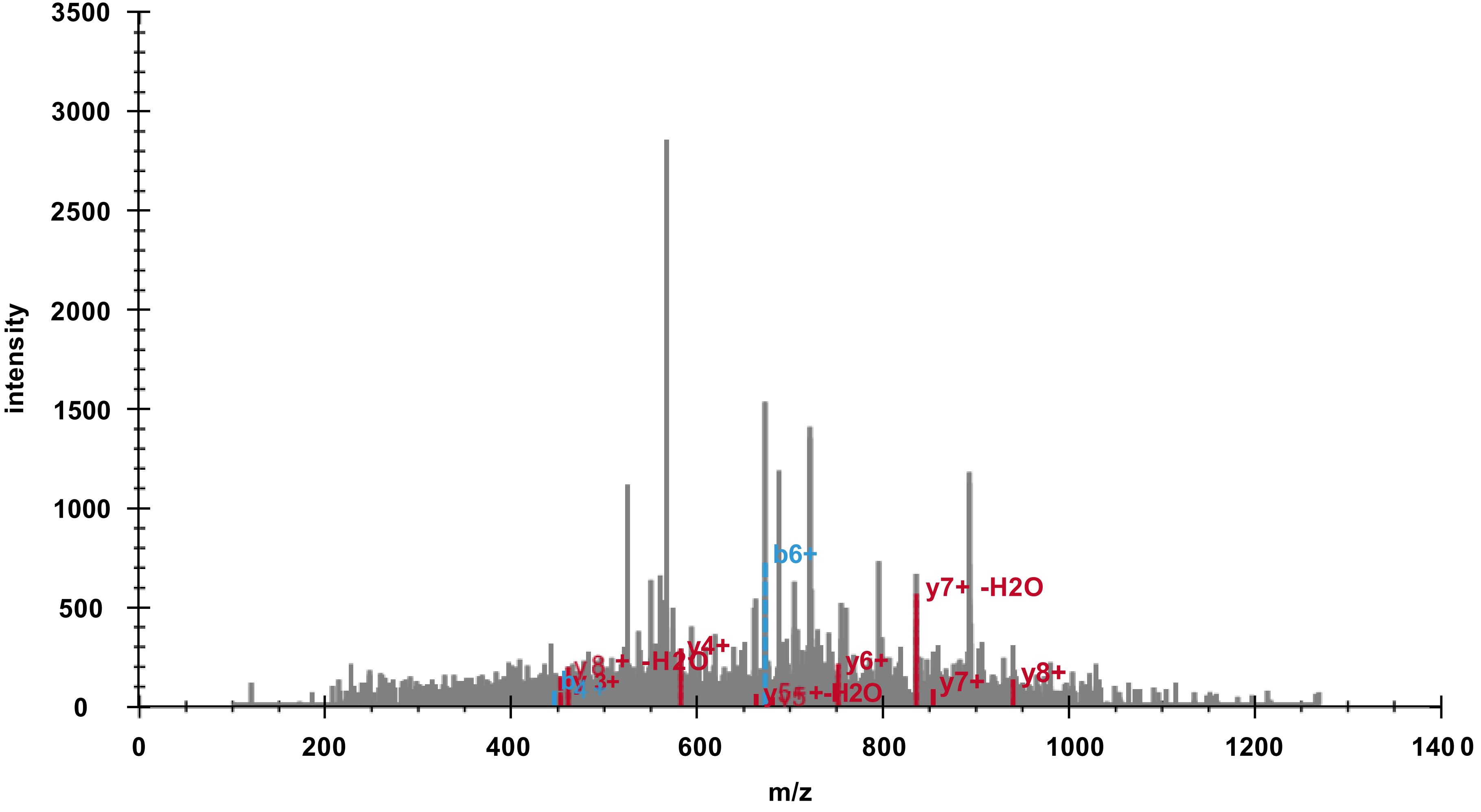

A0AAA9X0G7_[Acetyl (Protein N-term)]METGSDSDQLER__2

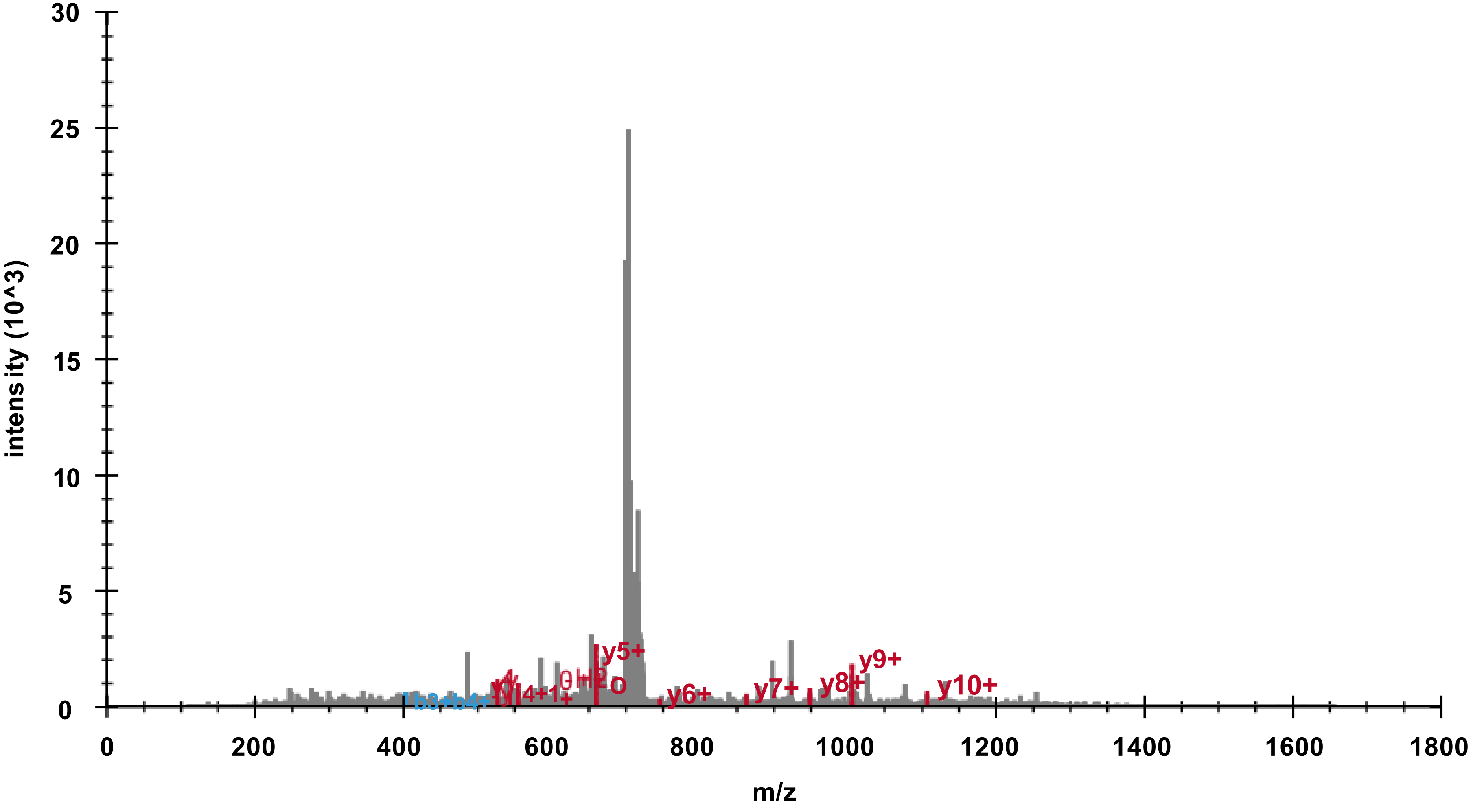

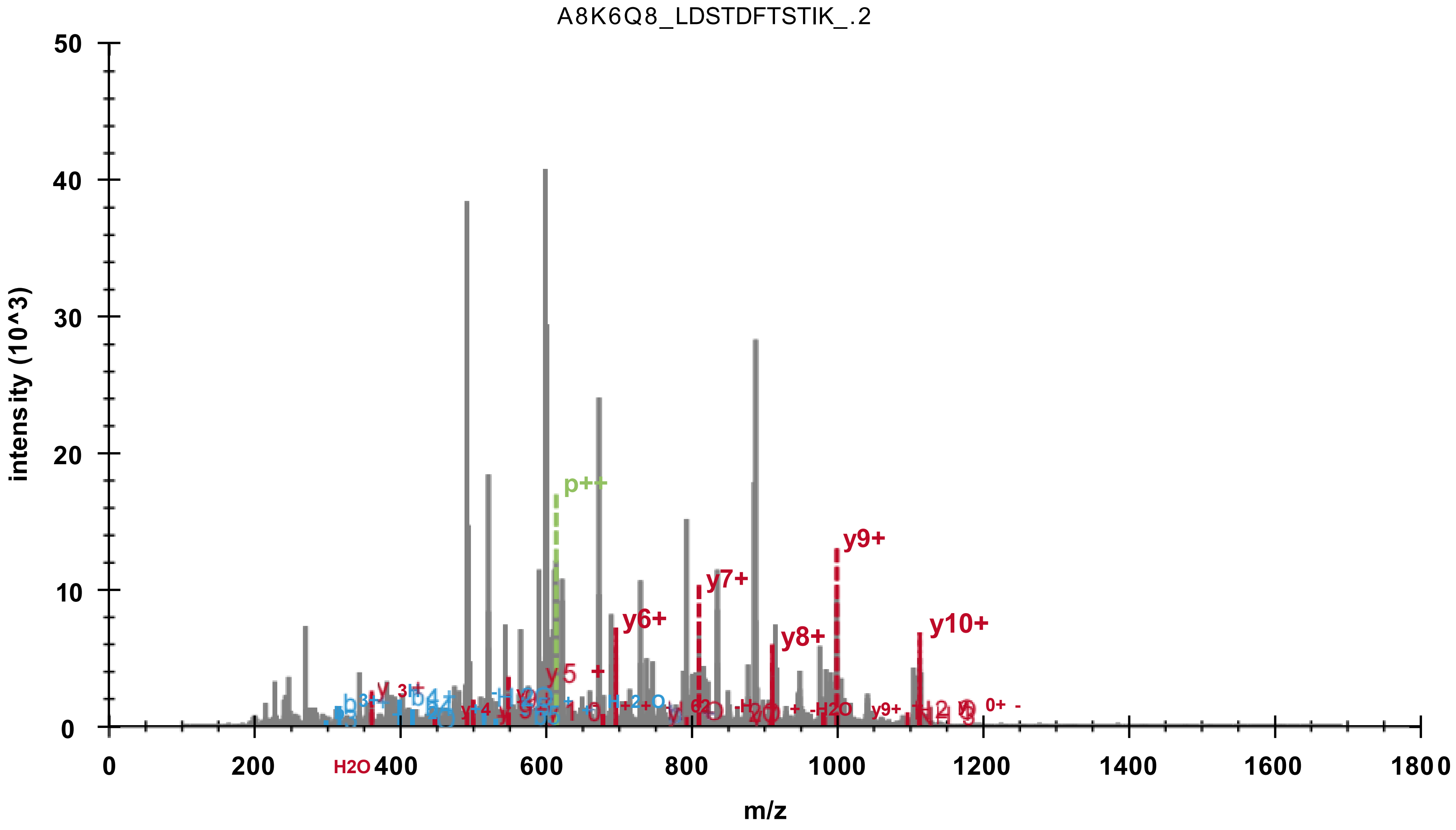

A8K6Q8_LDSTDFTSTIK_.2
intensity (10^3)
m/z
0
10
20
30
40
50
0
200
400
600
800
1000
1200
1400
1600
1800
p++
y9+
y7+
y6+
y8+
y10+

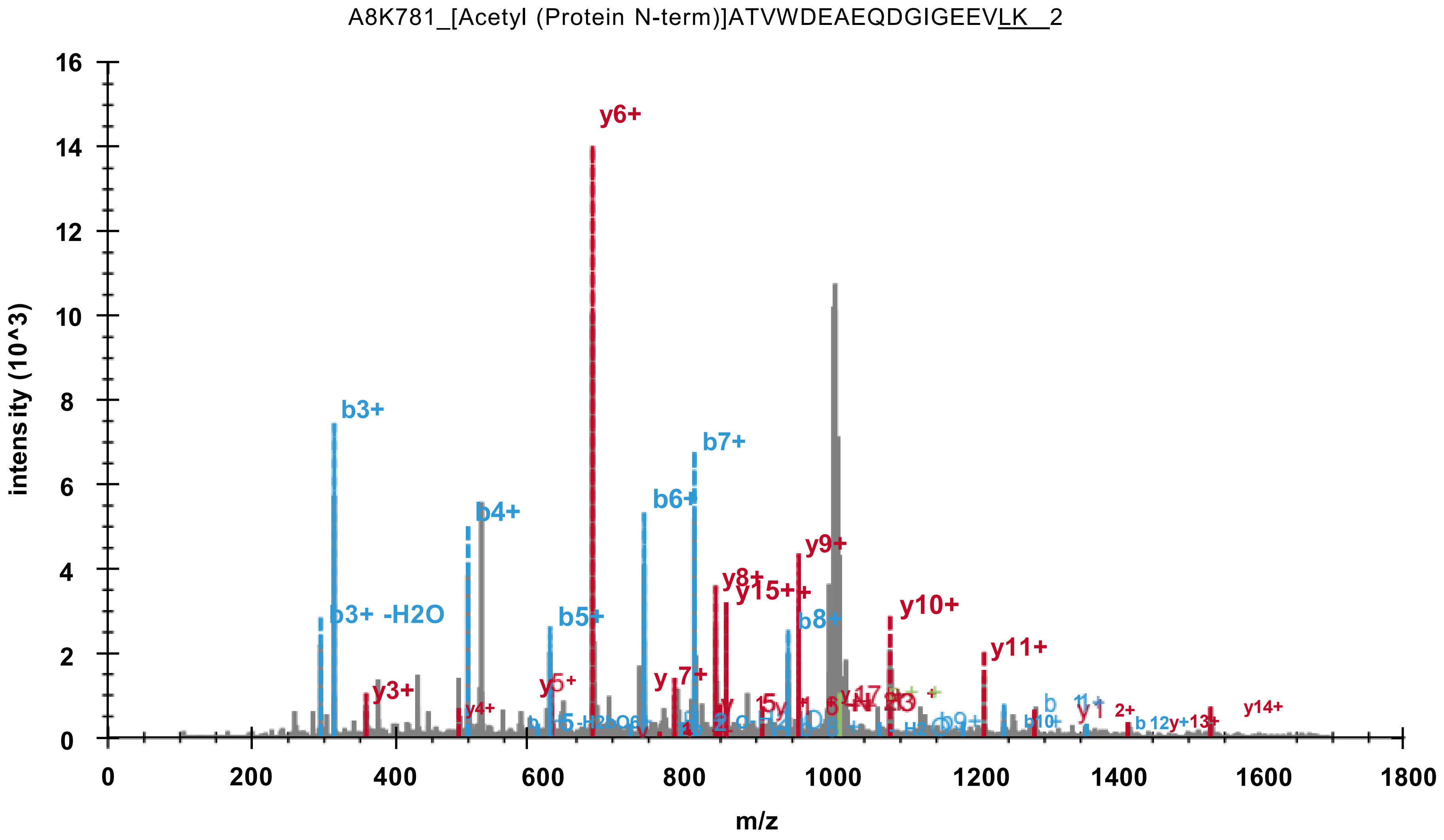
A8K781_[Acetyl (Protein N-term)]ATVWDEAEQDGIGEEVLK__2
intensity (10^3)
m/z
y6+
b3+
b4+
b7+
b6+
y9+
y8+
y15++
y10+
b3+ -H2O
b5+
b8+
y11+
y3+
y5+
y4+
y 7+
y14+
b10+
0
2
4
6
8
10
12
14
16
200
400
600
800
1000
1200
1400
1600
1800

B4DZ53_VNLTLGSIWDK_2

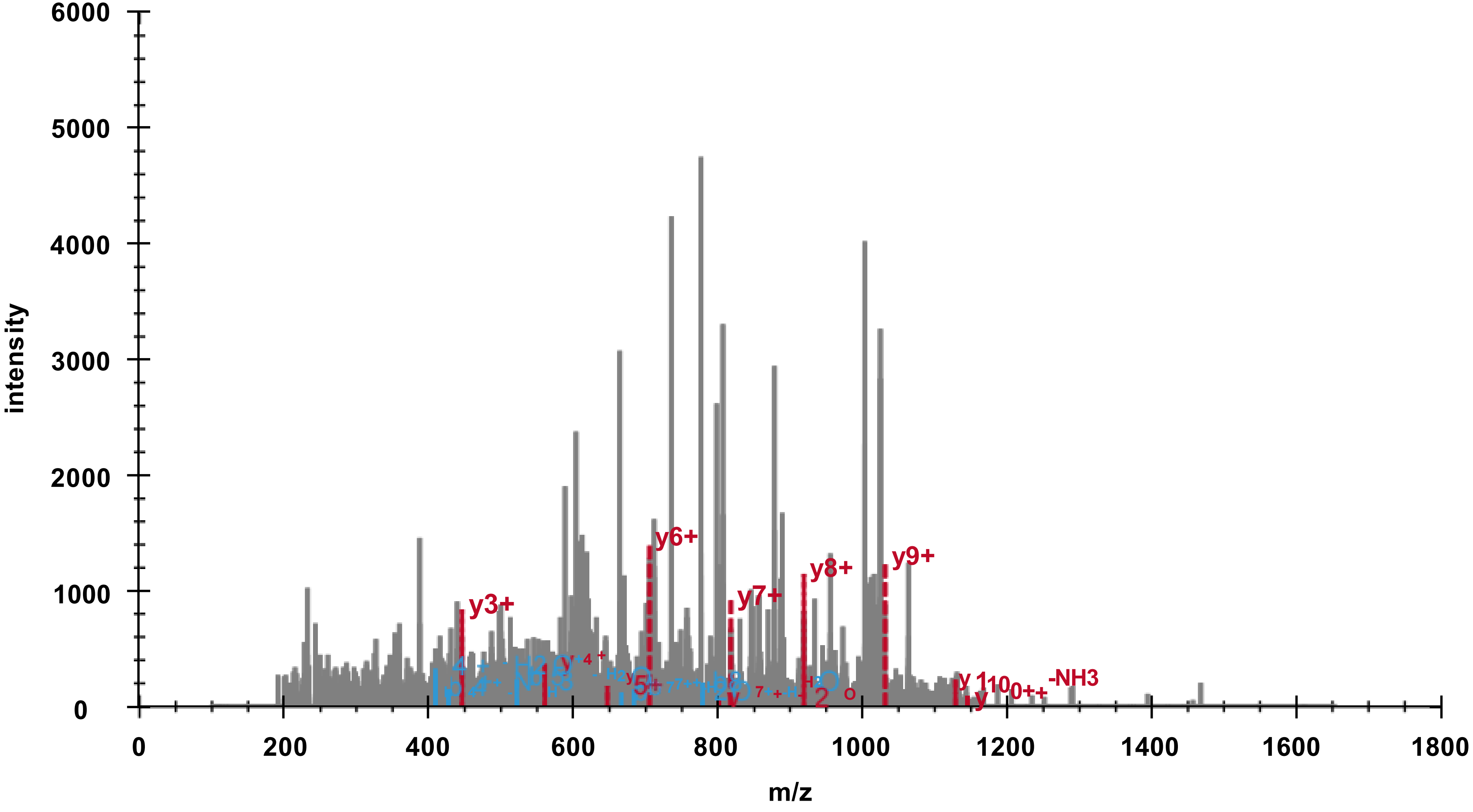

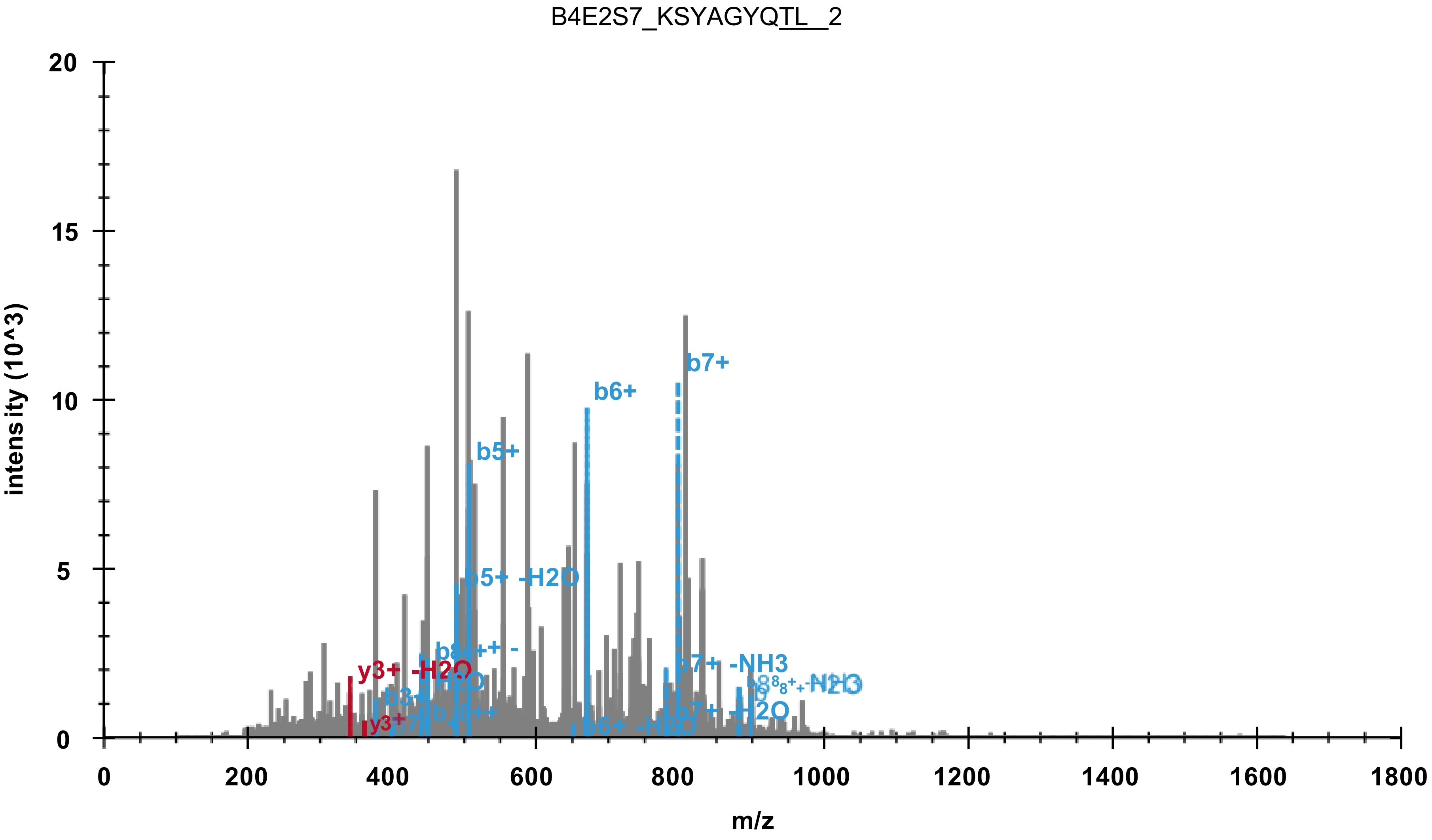

B4E2S7_KSYAGYQTL__2
intensity (10^3)
m/z
0
5
10
15
20
0
200
400
600
800
1000
1200
1400
1600
1800
b7+
b6+
b5+
b5+ -H2O
b7+ -NH3
y3+ -H2O
b3+
y3+
b6+ -H2O
b7+ -H2O

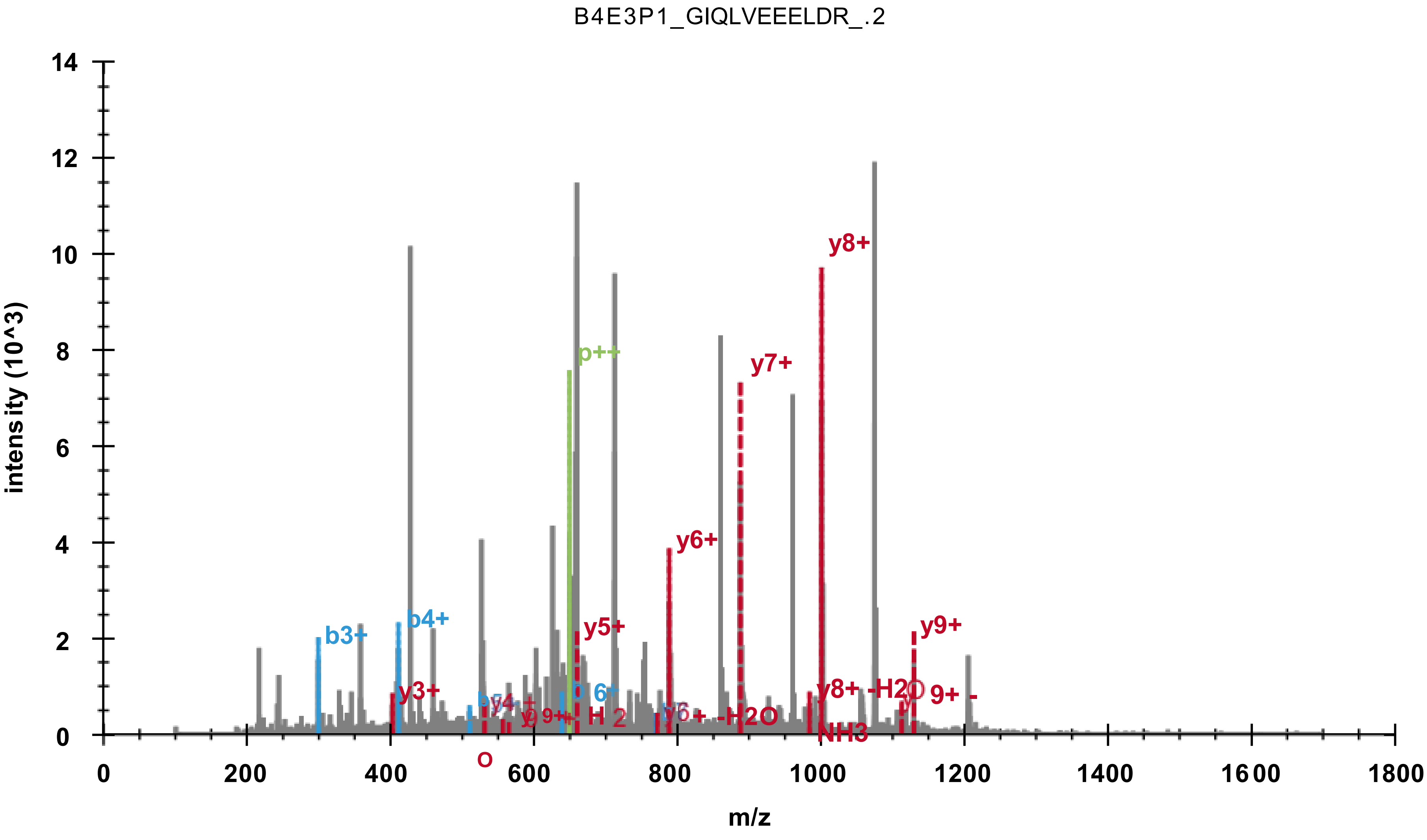
B4E3P1_GIQLVEEELDR_.2
intensity (10^3)
m/z
b3+
b4+
p++
y3+
y5+
y6+
y7+
y8+
y9+
b6+
y8+ -H2O
NH3
0
200
400
600
800
1000
1200
1400
1600
1800
0
2
4
6
8
10
12
14

B5BU78_YEDAVQFIR__2

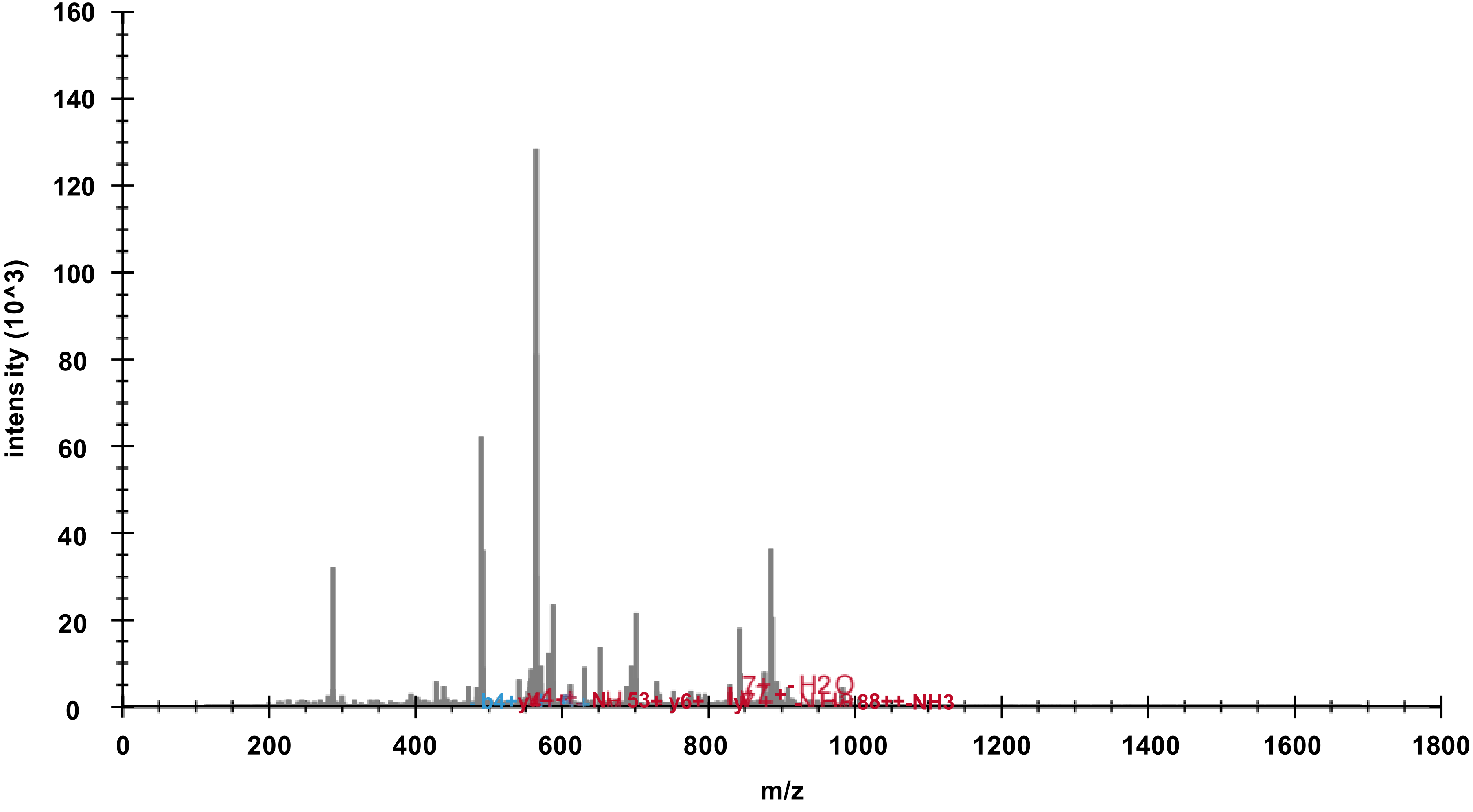

E9PKT4_TIDEHDAII_.2

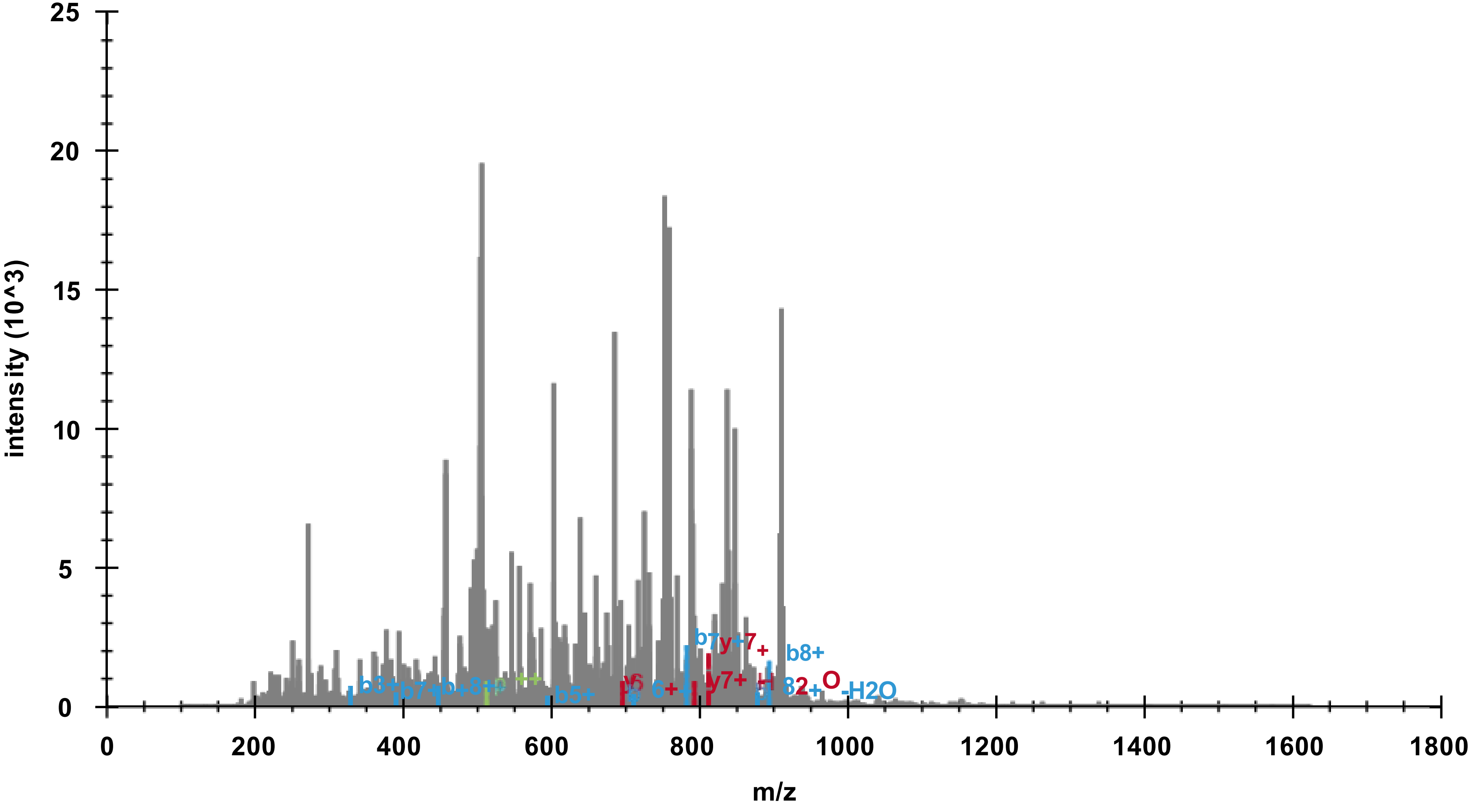

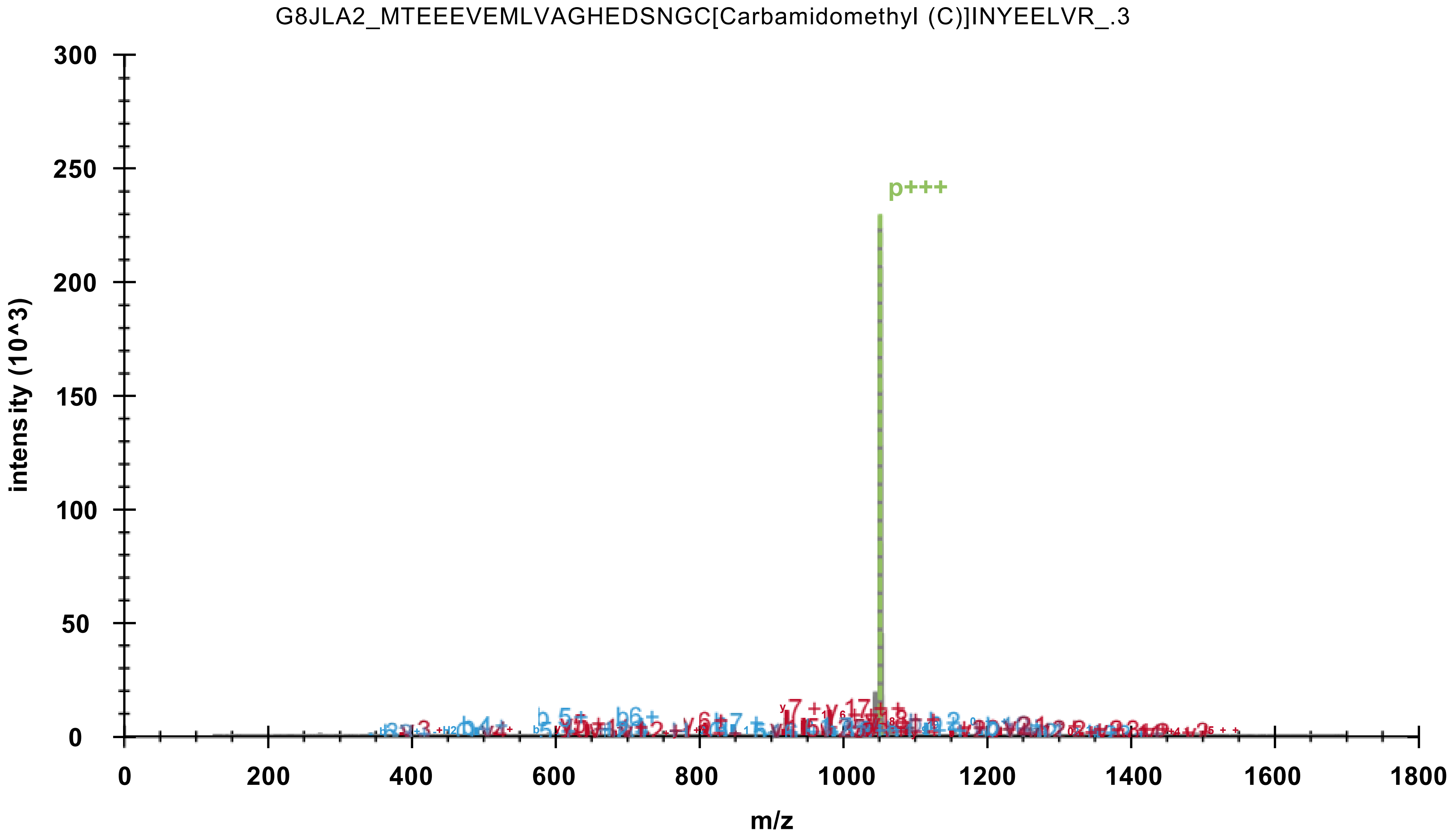
G8JLA2_MTEEEVEMLVAGHEDSNGC[Carbamidomethyl (C)]INYEELVR_.3
p+++
intensity (10^3)
300
250
200
150
100
50
0
0
200
400
600
800
1000
1200
1400
1600
1800
m/z

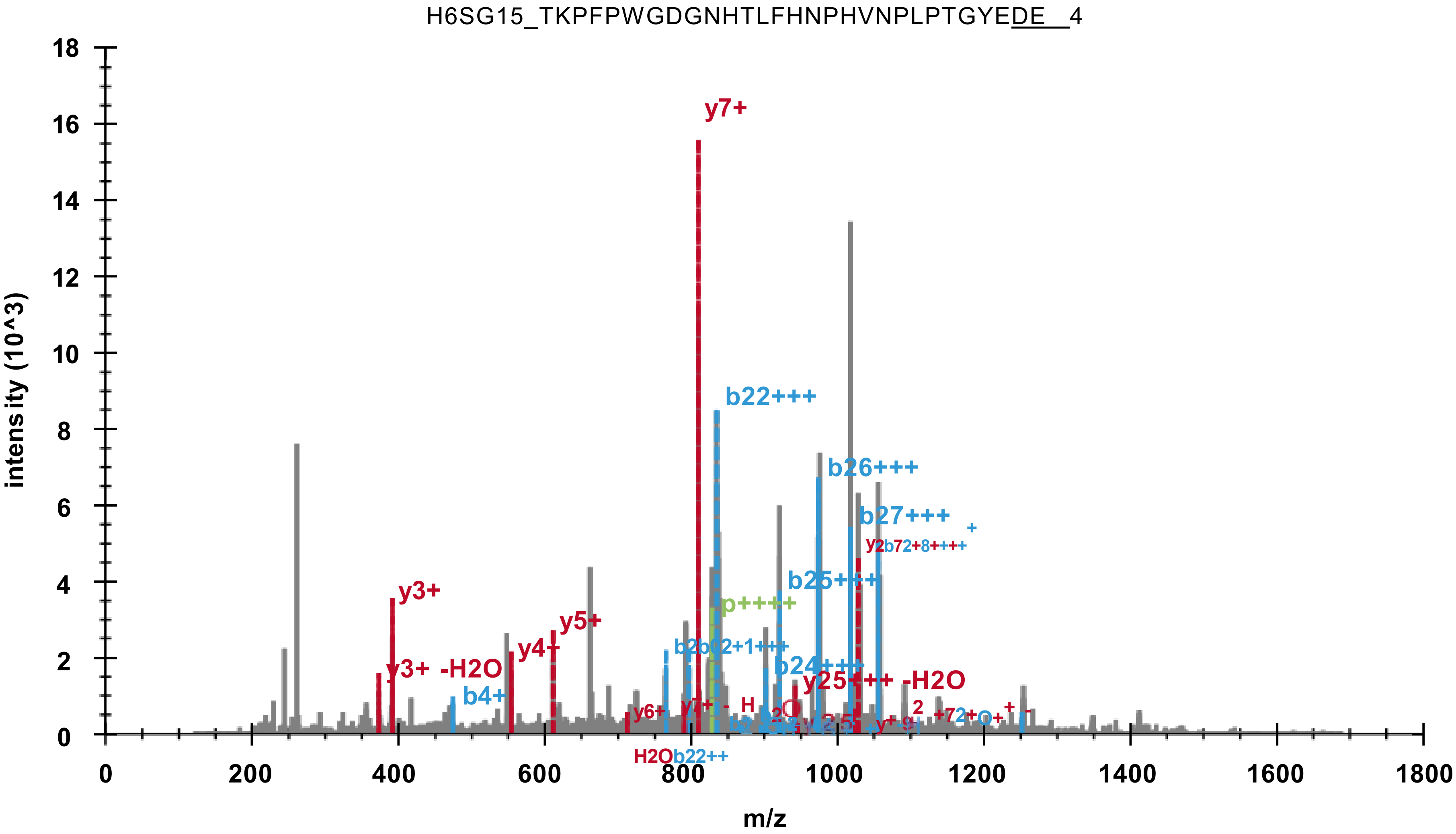

H6SG15_TKPFPWGDGNHTLFHNPHVNPLPTGYEDE__4
intensity (10^3)
m/z
y7+
b22+++
b26+++
b27+++
b25+++
p++++
y3+
y5+
y4+
y3+ -H2O
b4+
b24+++
y25+++ -H2O
y6+
H2Ob22++
0
2
4
6
8
10
12
14
16
18
200
400
600
800
1000
1200
1400
1600
1800

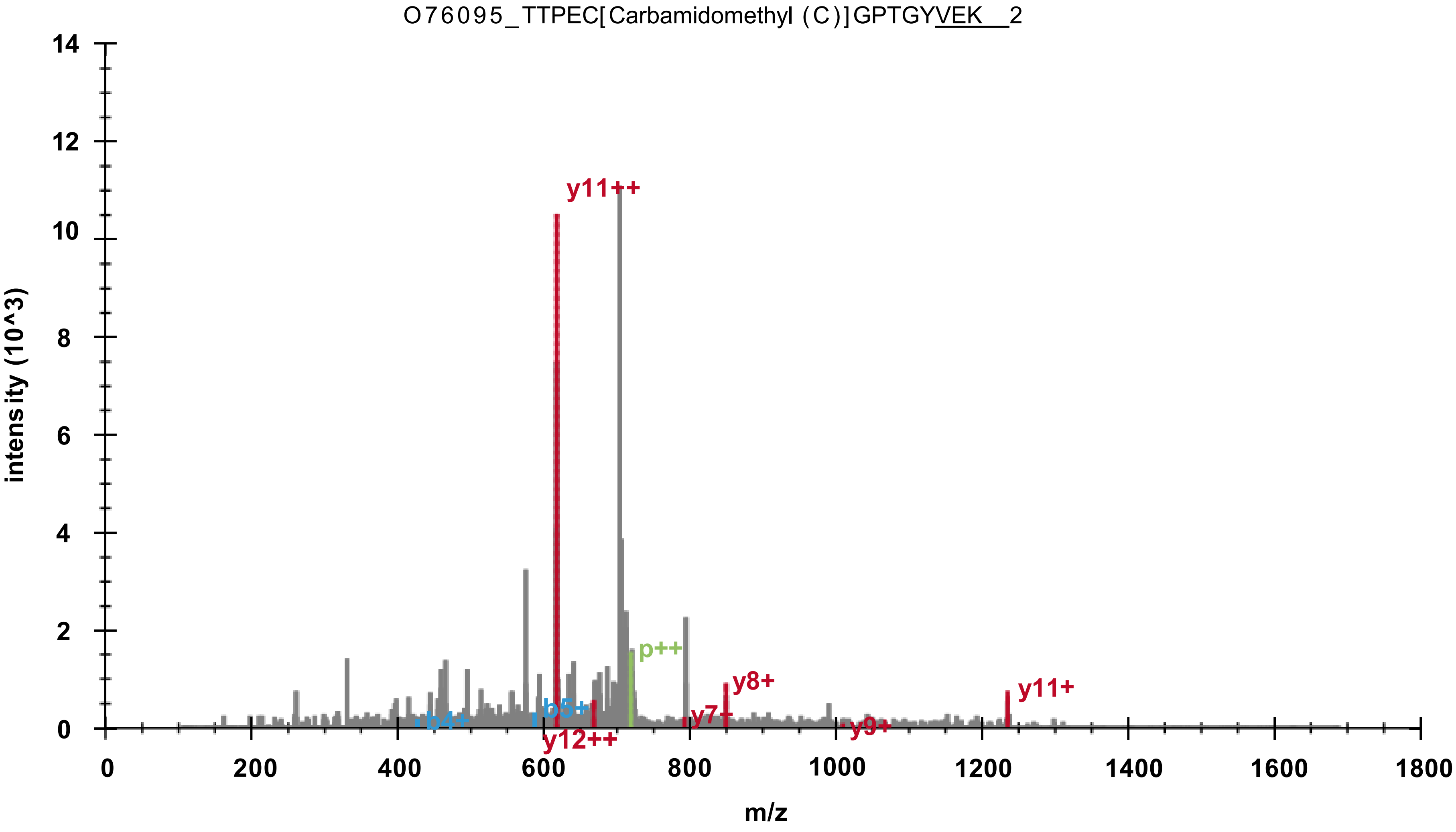

O76095_TTPEC[Carbamidomethyl (C)]GPTGYVEK__2
14
12
10
8
6
4
2
0
intensity (10^3)
y11++
p++
y8+
y11+
b5+
b4+
y7+
y9+
y12++
0
200
400
600
800
1000
1200
1400
1600
1800
m/z

P60709_AGFAGDDAPR_.2

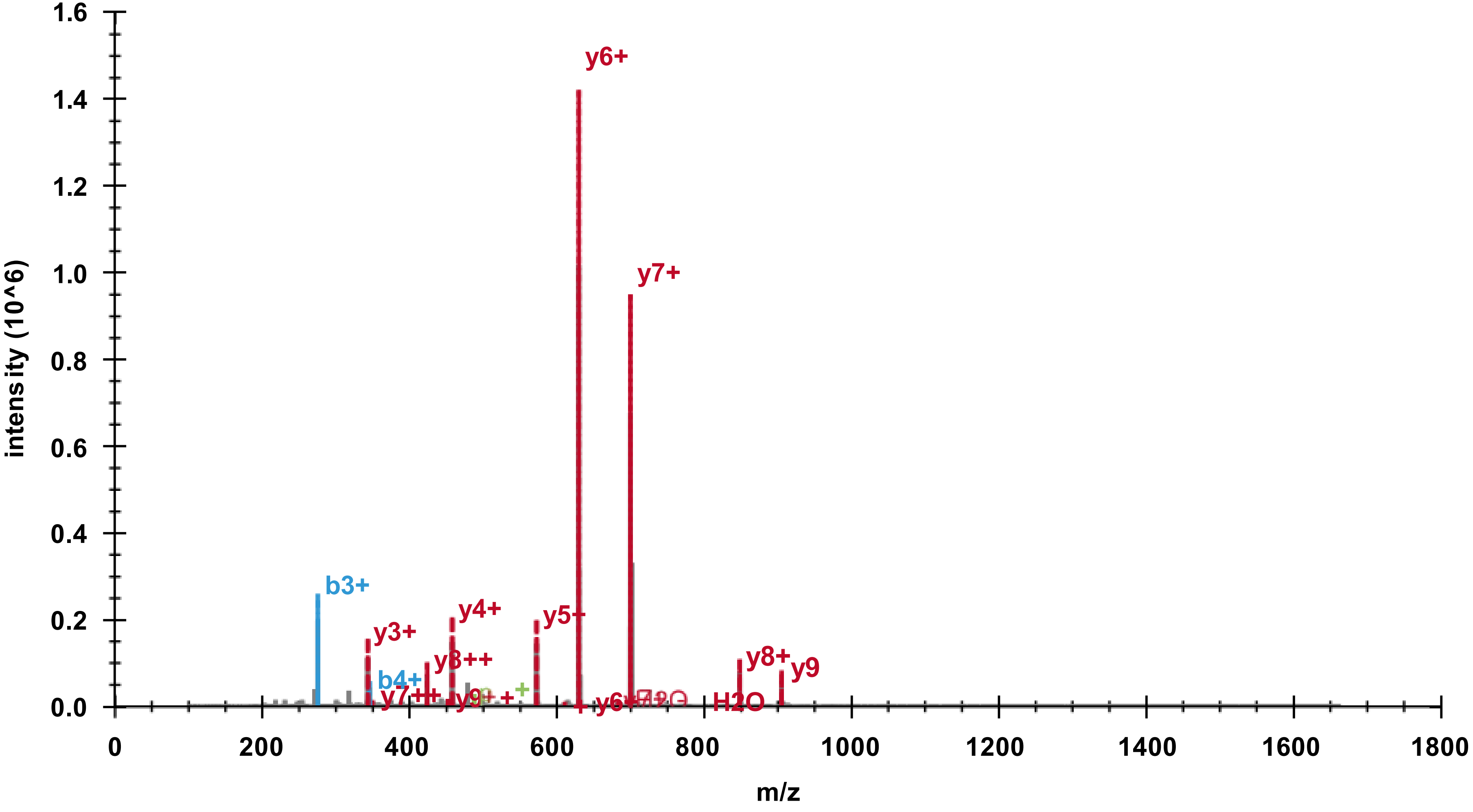

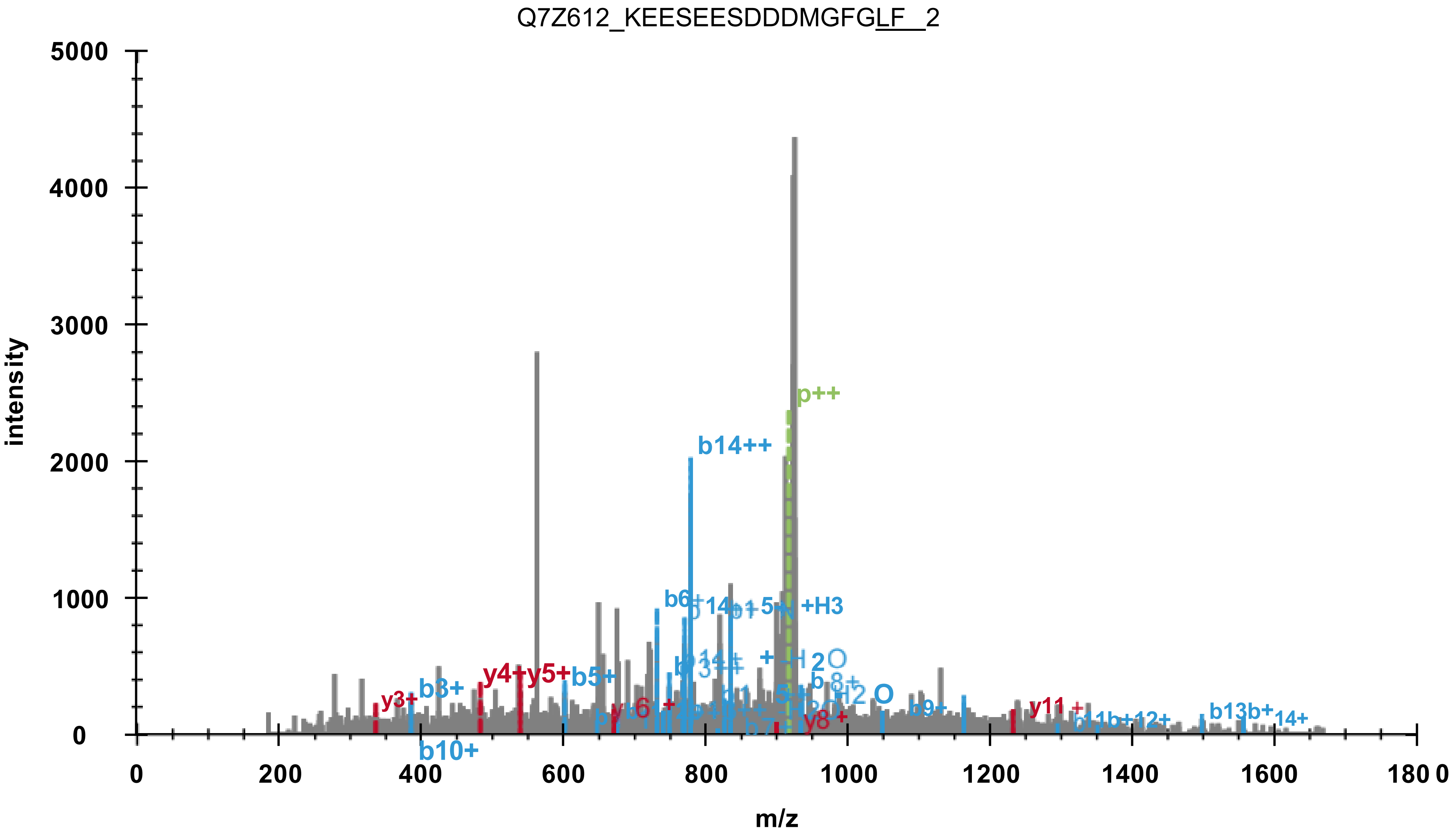

Q7Z612_KEESEESDDDMGFGLF__2
intensity
m/z
0
1000
2000
3000
4000
5000
200
400
600
800
1000
1200
1400
1600
1800
b14++
p++
y3+
b3+
y4+
y5+
b5+
b6+
b9+
y11
b10+
b13
y8

Q9NYG5_MAFNGC[Carbamidomethyl (C)]C[Carbamidomethyl (C)]PDC[Carbamidomethyl (C)]K__2

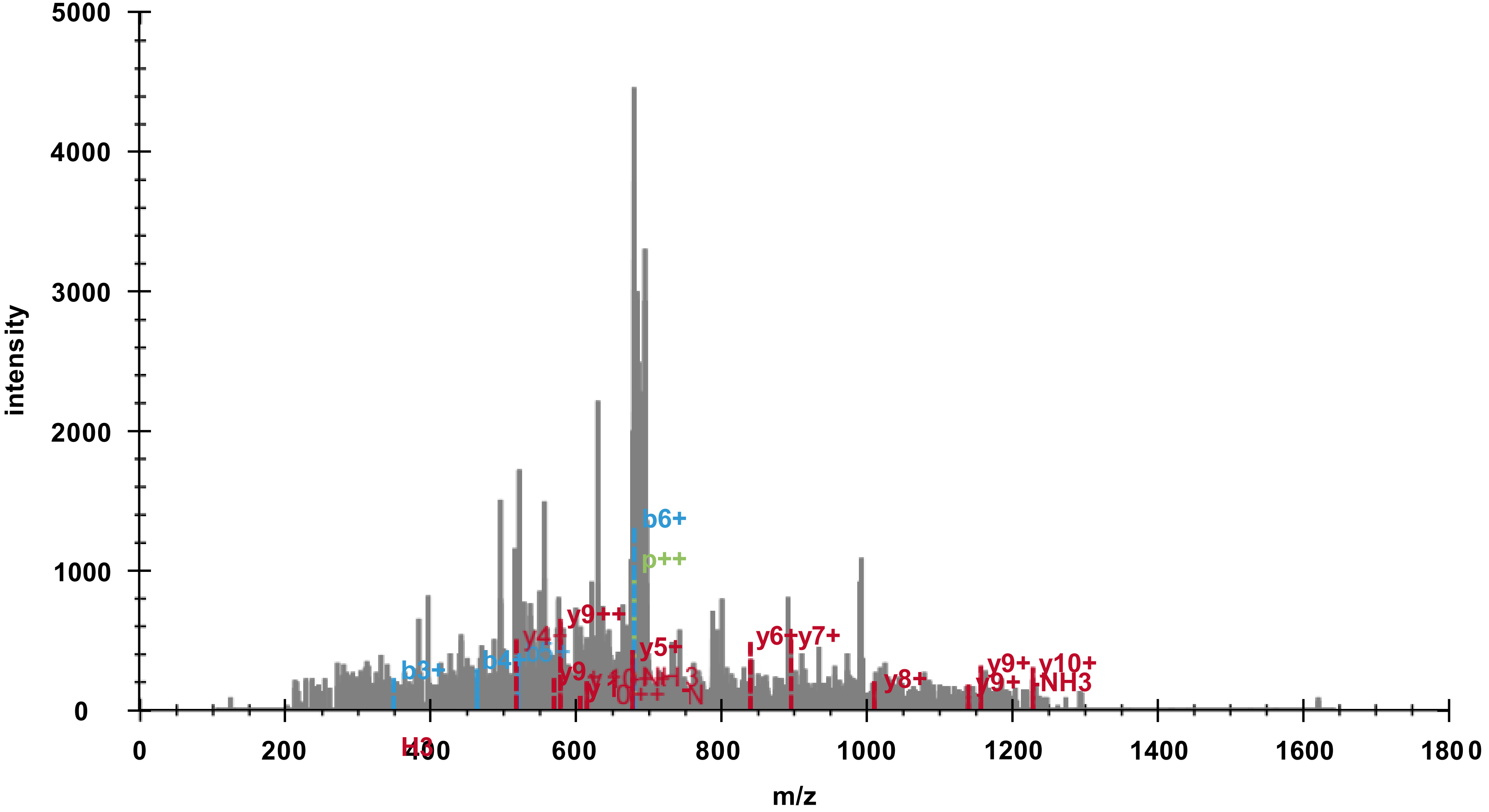

Supplement: Supplemental data S2_spectra for SUP [file mmc6.pdf]
